# Integrated optimization of electroplating wastewater treatment: A comparative assessment of physicochemical treatment configurations and process enhancement using response surface methodology

**DOI:** 10.1371/journal.pone.0345965

**Published:** 2026-08-21

**Authors:** Soumaya Ibrahimi, Aicha Gasmi, Karim Kriaa, Farid Fadhillah, Majeed Ali Habeeb, Djamel Ghernaout, Noureddine Elboughdiri, Ahmed Hannachi

**Affiliations:** 1 Department of Chemical Engineering, Laboratory of Engineering Processes and Industrial Systems, National School of Engineering of Gabes, University of Gabes, Gabes, Tunisia; 2 College of Engineering, Imam Mohammad Ibn Saud Islamic University (IMSIU), Riyadh, Saudi Arabia; 3 University of Babylon, College of Education for Pure Sciences, Department of Physics, Babil, Iraq; 4 Department of Chemical Engineering, College of Engineering, University of Ha’il, Ha’il, Saudi Arabia; Indian Institute of Technology Delhi, INDIA

## Abstract

This study assesses physicochemical treatment methods for industrial electroplating wastewater (EPW). Monitoring at a full-scale Tunisian facility revealed considerable instability in conventional systems. Residual nickel concentrations ranged from 0.02 ± 0.01 to 60 ± 0.4 mg/L. Chloride levels consistently exceeded regulatory thresholds, ranging from 442 ± 9–1418 ± 10 mg/L. Over 24 weeks, three treatment configurations were evaluated: ferric chloride-based coagulation (Configuration 1), polyaluminum chloride coagulation (Configuration 2), and lime slurry-based chemical attack (Configuration 3). Configuration 3 showed the most consistent performance. It achieved 99% chromium removal, 97% nickel removal, and 50% chloride reduction, while reducing chemical operating costs by 69% (0. 134 €· m ³ compared to 0. 432 €·m^−3^). A novel optimization strategy is presented that explicitly incorporates variability in influent nickel concentration ([Ni]ᵢ, 33–92 mg/L) as an independent factor. This is applied within a Response Surface Methodology–Central Composite Design, alongside pH (6.3–9.7) and flocculant dosage (2.2–9.4 mg/L). Fluctuations in wastewater composition are treated as a controlled design variable rather than experimental noise. This approach produces a robust predictive model ([Ni]_f_ = f([Ni]ᵢ, pH, [A-PAM]) with high accuracy (*R*² = 0.9917). The resulting equation enables real- time adaptive chemical dosing. Operators can calculate optimal pH and flocculant requirements based on measured influent concentrations. Statistical analysis identified significant pH-metal loading interactions (*F* = 5.70, *p* = 0.044). This confirms that optimal parameters systematically shift with influent composition. Under model-predicted conditions, residual nickel consistently remained below 2 mg/L, despite substantial influent variability. This ensures regulatory compliance. The integrated approach shows that variability- responsive optimization enhances treatment efficacy, operational stability, and economic efficiency.

## 1. Introduction

Electroplating is essential in modern manufacturing, adding surface layers that help prevent rust, improve strength, and enhance the appearance of products across industries such as automotive, electronics, and aerospace, as well as everyday items [1]. However, these processes produce wastewater with high levels of heavy metals (HMs)—such as chromium, nickel, copper, zinc, and lead—along with saline water, changes in acidity, and numerous organic pollutants from additional chemicals, soaps, and cleaners [2,3]. Wastewater from electroplating can also contain new types of contaminants, such as N-nitrosamines [4], which are worrisome because they can cause cancer and persist in water. If this wastewater is not properly treated, it can harm both the environment and public health due to the toxic and long-lasting nature of heavy metals and these new contaminants [5,6]. Although a full study would examine all types of pollutants in this wastewater, this research primarily focuses on removing heavy metals first, because regulators monitor them most closely and they cause most of the toxic effects. The main reason is that removing heavy metals directly addresses the biggest environmental issues. It is expected that some of the new contaminant sources will also be partially removed along with organic matter during chemical treatment, as indicated by lower chemical oxygen demand (COD) [5,6].

Among heavy metal (HM) contaminants, hexavalent chromium (Cr(VI)) and divalent nickel (Ni(II)) are of particular concern because of their extensive use in electroplating and well-documented toxicological effects [7]. Cr(VI) is associated with carcinogenicity and acute ecotoxicity [2], whereas nickel is linked to carcinogenic, allergenic, and chronic ecological effects [8].

Consistent removal of these contaminants by conventional physicochemical treatments remains technically challenging for two primary reasons. First, chloride-based coagulants such as ferric chloride (FeCl_3_), commonly used for heavy metal precipitation, introduce additional chloride into treated effluents. This can result in chloride concentrations exceeding discharge standards and limit opportunities for water reuse [2,9]. Second, strong metal–organic complexes formed by plating additives can inhibit standard precipitation mechanisms, allowing residual dissolved metals to persist even when chemical dosing and pH are appropriately controlled [2,10].

Consequently, discharge regulations have become increasingly stringent worldwide, necessitating treatment systems that maintain stable compliance under variable operating conditions [11,12]. Within this regulatory framework, Tunisia has adopted the NT 106.002 standard (INNORPI, Tunisian Institute of Standardization) as a mandatory reference for industrial effluent quality control. The standard sets maximum permissible limits for physicochemical parameters and is particularly applicable to metal-finishing processes, including electroplating [11]. Specifically, NT 106.002 establishes the following limits for the primary pollutants addressed in this study: for discharge into the Public Hydraulic Domain (PHD), the maximum allowable concentrations are 0.5 mg/L for Cr(III), 0.2 mg/L for Ni(II), and 600 mg/L for Cl^−^. For discharge into the public sewerage network (ONAS), the respective limits are 2 mg/L, 1 mg/L, and 700 mg/L.

Multiple strategies are used to treat EPW, including chemical precipitation [13], coagulation-flocculation [9], electrochemical treatment [14,15], adsorption [16,17], electrodialysis [18], and membrane filtration [19]. Advanced sequential treatment approaches have been developed to address the complexity of EPW pollutants [20–23]. Despite these advancements, each technology has practical limitations that restrict its standalone industrial applicability [24,25]. Electrochemical methods, although effective, require high energy input and significant capital investment [14]. Adsorption processes require expensive regeneration or disposal of saturated media [16]. Membrane filtration and electrodialysis are prone to fouling and incur substantial operational costs, especially at elevated pollutant concentrations [18,19]. As a result, chemical treatment, particularly precipitation and coagulation-flocculation, remains fundamental to EPW management at the industrial scale due to its demonstrated effectiveness at high pollutant concentrations, operational simplicity, and comparatively low cost [2]. Nevertheless, the performance of chemical treatment is highly sensitive to operating conditions. It is often unstable in industrial environments, primarily due to fluctuations in wastewater composition arising from production cycles, bath renewal, and rinsing practices [1].

Despite extensive research, significant knowledge gaps remain regarding the long-term performance, operational robustness, and economic sustainability of physicochemical (PC) treatment systems under real-world industrial conditions. Most studies are limited to laboratory-scale experiments or short-term trials using synthetic or compositionally stable wastewaters [7,10,14], which do not capture the temporal and compositional variability of actual industrial effluents [1]. For example, Bhagavath et al. [26] achieved satisfactory nickel removal with synthetic solutions but observed substantial performance declines when treating real effluents, underscoring the limitations of laboratory-based findings. At full scale, Gao et al. [1] reported persistent treatment instability in an eight-stage automated system, attributing recurrent non-compliance to improper dosing and insufficient adaptation to influent variability. This evidence shows that operational complexity alone does not ensure treatment robustness. Furthermore, process optimization has traditionally relied on univariate methods or statistical designs that assume constant influent characteristics, neither of which accurately reflects industrial conditions, and both of which limit system adaptability [27].

In this context, Response Surface Methodology (RSM) is a powerful yet underutilized approach for systematically optimizing treatment processes for electroplating wastewater (EPW) [28,29]. However, most RSM-based studies do not explicitly incorporate variability in influent metal concentration as a design parameter, instead treating it as experimental noise or artificially stabilizing it. Overcoming this limitation is critical to developing adaptive, robust treatment strategies that maintain high performance under real-time fluctuations in the influent.

This study presents an integrated experimental and analytical framework to evaluate, optimize, and economically assess alternative PC treatment configurations for industrial EPW. The primary objectives are as follows: (i) to assess the long-term performance of a full-scale conventional treatment system under real-world operating conditions; (ii) to compare three configurations usinPCg FeCl_3_, polyaluminum chloride (PAC), and lime slurry (LS) in terms of efficiency, stability, and economic performance; (iii) to conduct a comparative economic analysis focused on chemical consumption costs; and (iv) to implement an RSM-based optimization strategy that explicitly incorporates variability in influent nickel concentration as an independent design variable, along with pH and flocculant dosage. This approach is applied to EPW treatment for the first time.

Explicitly incorporating influent variability into the optimization framework moves beyond conventional static treatment design and enables a variability-aware, predictive control strategy. This approach supports the development of a quantitative dosing model that links influent metal concentration to optimal operating conditions, providing a practical tool for real-time process adjustment. The study’s primary contribution is transforming influent variability from a limiting constraint into a central optimization parameter, offering a robust pathway toward more resilient, cost-effective, and environmentally sustainable EPW treatment under variable industrial conditions.

## 2. Materials and methodology

### 2.1 Conventional physicochemical (PC) treatment process and electroplating wastewater (EPW) segregation

EPW samples analyzed in this study were collected from the industrial facility under investigation in Tunisia. The samples were segregated at the source by chemical type to prevent incompatible reactions and improve treatment efficiency. Specifically, chromic acidic effluents (E1) and alkaline effluents (E2) were managed separately because of their distinct chemical properties and treatment requirements. Chromic acidic effluents (E1) primarily originated from surface treatment and rinsing operations, whereas alkaline effluents (E2) were generated during degreasing and alkaline cleaning stages.

The industrial treatment process uses a conventional PC scheme widely implemented in electroplating facilities. This approach consists of the following sequential unit operations:

**Dechromatization:** E1 effluents initially undergo a chromium reduction process, in which highly toxic chromiuhexavalentm (Cr(VI)) is converted to the less toxic trivalent form (Cr(III)) by adding sodium bisulfite (NaHSO_3_) under strongly acidic conditions (pH 1.8–2.0). At this stage, the resulting Cr(III) ions remain in solution and are subsequently removed by hydroxide precipitation during the downstream neutralization step.
**Coagulation:** The dechromatized E1 effluents are combined with the alkaline E2 streams to produce a mixed effluent that undergoes coagulation. In this step, a chemical coagulant is introduced under rapid agitation to destabilize colloidal particles and suspended matter through charge neutralization and sweep floc formation. The effectiveness of destabilization is monitored using zeta potential measurements, which quantitatively assess the electrokinetic stability of the colloidal suspension. Optimal coagulation is achieved when the zeta potential approaches neutrality (approximately 0 mV), indicating that electrostatic repulsive forces have been sufficiently reduced to permit interparticle aggregation [30]. This process facilitates the formation of primary microflocs that act as nucleation sites for subsequent metal hydroxide precipitation, while also reducing turbidity and enhancing the settling characteristics of the treated effluent.
**Neutralization (chemical precipitation):** After coagulation, the pH of the mixed effluent is adjusted to 6.5–9.0 by adding an alkaline reagent, which supplies hydroxide ions to precipitate Cr(III) and Ni(II) as insoluble metal hydroxides. The speciation and solubility of both metals depend strongly on pH. For Cr(III), minimum solubility occurs between pH 7.5 and 9.0. Above pH 10, amphoteric dissolution becomes significant due to the formation of the soluble chromate anion [Cr(OH)_4_]^−^, which can cause andredissolution deterioration of effluent quality [31]. For Ni(II), minimum solubility occurs in the pH range 8.5–9.5. Above pH 10–11, partial formation occurs through redissolution of soluble hydroxo-complexes [Ni(OH)_3_]^−^ and [Ni(OH)_4_]^2−^ [32]. Therefore, maintaining an operational pH of 8.5–9.0 is the optimal compromise for effective simultaneous precipitation of both metals in the bimetallic Cr–Ni system [33].
**Flocculation:** After precipitation, an anionic polyacrylamide (A-PAM) flocculant is added under slow agitation. This promotes aggregation of primary O(NiH)_2_ and Cr(OH)_3_ microflocs into larger, settleable aggregates via polymer bridging.
**Solid–liquid separation and sludge dewatering:** The flocculated metal hydroxide sludge is separated from the clarified effluent by gravitational settling. The settled sludge is then dewatered using a filter press and sent to an approved disposal or valorization facility.
**Final pH adjustment:** Before discharge, the clarified and filtered effluent undergoes a final pH correction to ensure compliance with applicable discharge standards.

The effluent from mixing the dechromatized E1 and alkaline E2 streams, before coagulation and pH adjustment, serves as the initial reference wastewater (E3) used throughout this study.

### 2.2 Performance monitoring of the final treated electroplating wastewater (f-EPW) after actual process

This assessment aims to characterize the temporal variability of treated EPW under real-world process conditions and to evaluate how fluctuations in influent quality affect overall treatment performance. Wastewater quality and operating conditions of the reference treatment process were comprehensively characterized by collecting samples at key points along the treatment line, including the treatment stage (E3) and the final clarified effluent (f-EPW).

Weekly sampling campaigns were conducted over one full operational year, from January 2, 2024, to March 20, 2025, under the precipitation-based treatment process. This approach captured temporal variability associated with production schedules, operational fluctuations, and seasonal effects. After collection, samples were preserved at 4°C and transported to the laboratory for analysis. All PC analyses were completed within 48 hours to minimize degradation and ensure data reliability.

The monitored parameters included pH, electrical conductivity (ECw), total suspended solids (TSSs), chemical oxygen demand (COD), biochemical oxygen demand (BOD₅), chloride concentration, and HM content (i.e., Cr³⁺ and Ni²⁺).

Table 1 presents a comparative analysis of E3 and f-EPW, highlighting both the effectiveness and critical limitations of the current treatment system. The process achieves complete pH remediation, demonstrating effective pH neutralization. However, organic matter reduction is inconsistent, with COD removal efficiencies ranging from 49% to 90% and BOD₅ reductions from 20% to 86%. While minimum f-EPW values meet regulatory standards, substantial fluctuations and peak concentrations exceed NT 106.002 thresholds. The TSS of the initial effluent E3 is inherently negligible (2 ± 0.3 mg/L), indicating that turbidity, governed by the suspended particulate load, is also negligible and does not serve as a meaningful performance indicator or limiting parameter for this specific industrial wastewater matrix. The observed TSS variability in f-EPW (2 ± 1–118 ± 2 mg/L) is attributable to operational inconsistencies in the settling stage rather than to coagulation deficiency, confirming that suspended solids removal is not the primary challenge in this treatment system. In contrast, the most significant deficiencies are associated with HMs and chloride concentrations, which have been identified as the primary limiting factors for regulatory compliance and environmental impact. These parameters, therefore, constitute the central focus of the comparative performance assessment in this study.

**Table 1:** Temporal variations in key physicochemical (PC) parameters of electroplating wastewater (EPW) and comparisons with regulatory discharge limits. (COD: Chemical Oxygen Demand; EC_w_: Electrical Conductivity of Water; f-EPW: final treated electroplating wastewater; TSS: Total Suspended Solids; E3: Initial Reference Wastewater).

Parameter	E3	f-EPW	Discharge limits NT 106.002	
			PHD^a^	ONAS^b^
pH	2.1 ± 0.5	6.5 ± 0.1-9 ± 0.1	6.5-8.5	6.5-9
TSS (mg/L)	2 ± 0.3	2 ± 1-118 ± 2	30	400
EC_w_ (μS/cm)	7923 ± 22-261 ± 6	2720 ± 10-6100 ± 14	–	–
COD (mg/L)	299 ± 1-464 ± 2	30 ± 3-235 ± 8	90	1000
BOD_5_ (mg/L)	74 ± 1-226 ± 0.9	10 ± 3-180 ± 14	30	400
Cl^−^ (mg/L)	1171 ± 2-1815 ± 8	442 ± 9-1418 ± 11.2	600	700
Cr^3+^ (mg/L)	70 ± 1-132 ± 3	0.01 ± 0.002-6.03 ± 0.09	0.5	2
Ni (mg/L)	33 ± 1-92 ± 1	0.02 ± 0.01-60 ± 0.4	0.2	1

^a^NT 106.002 for discharge into Public Hydraulic Domain (PHD).

^b^NT 106.002 for discharge into National Office of Sanitation (ONAS) public sewerage network.

**Fig 1:**
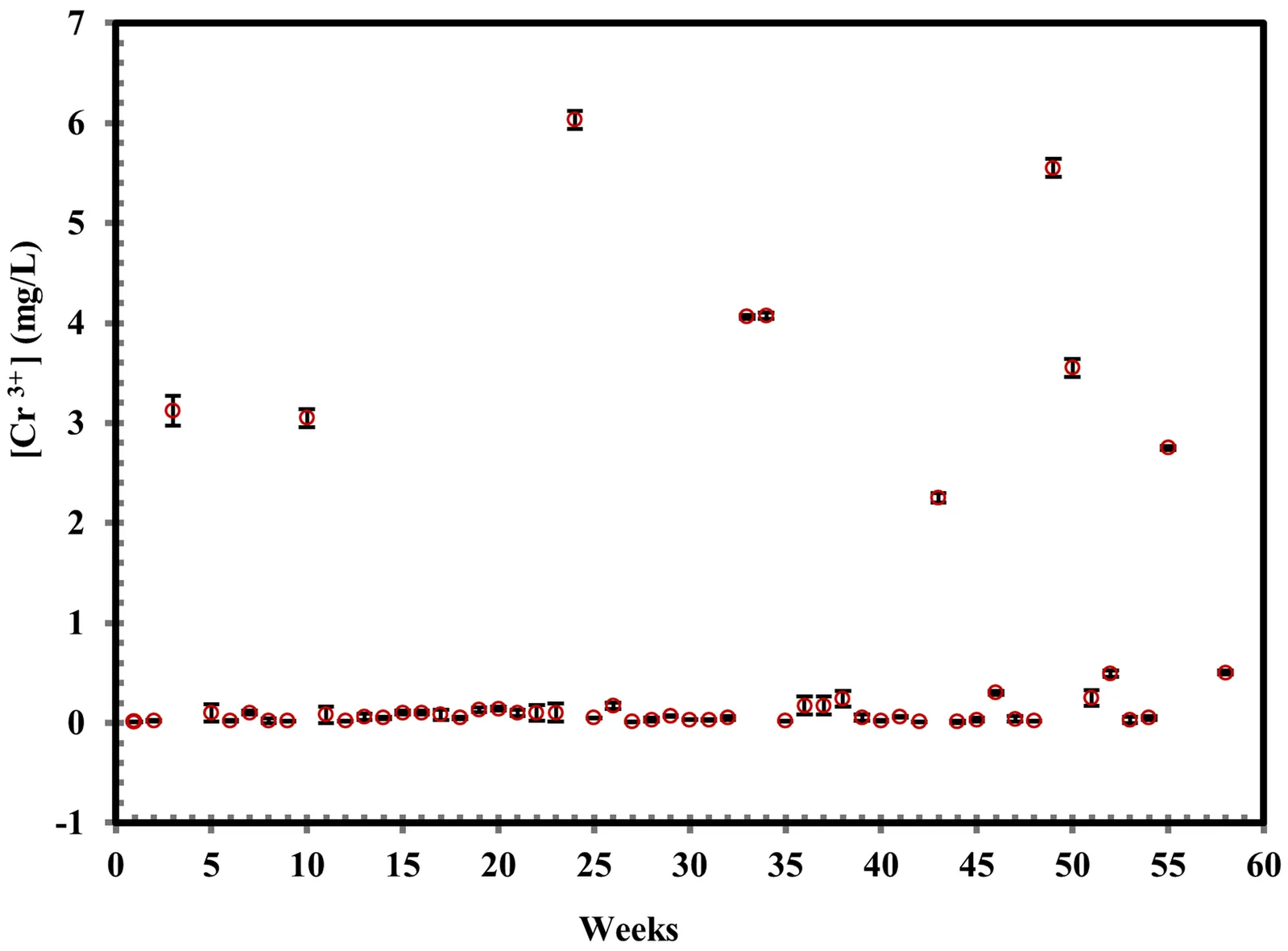
Temporal variation in Cr^3+^ concentration in final treated electroplating wastewater (f-EPW).

The temporal evolution of trivalent chromium (Cr(III)) concentrations (Fig 1) indicates that Cr(III) levels remained relatively low during most of the operational period; however, sporadic peaks reaching up to 6.03 ± 0.09 mg/L were recorded, occasionally exceeding the discharge limit of 2 mg/L. These episodic exceedances suggest that the conventional process is sensitive to sudden changes in influent composition and operational disturbances.

**Fig 2:**
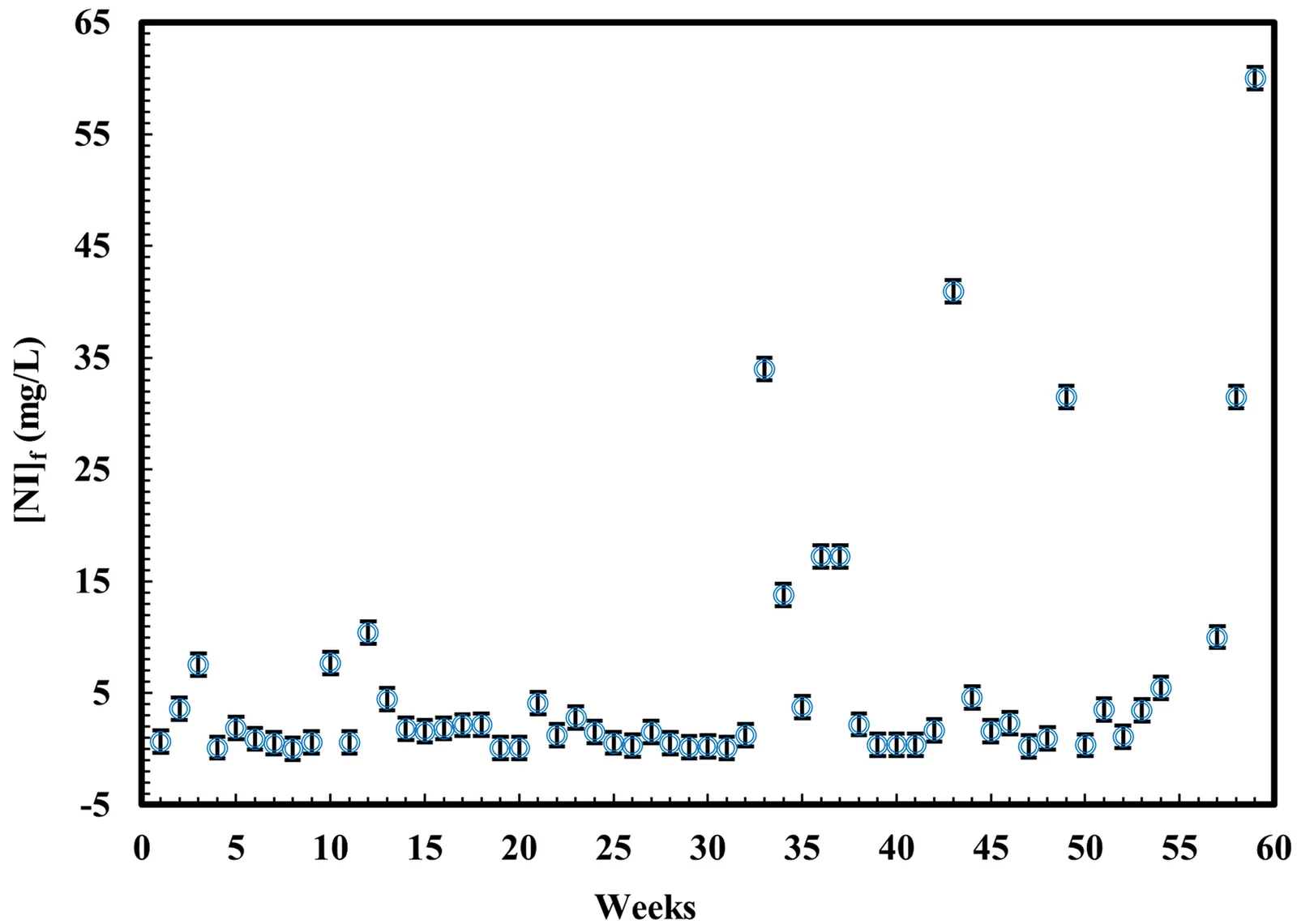
Temporal variations in nickel (Ni) concentration in the final treated electroplating wastewater (f-EPW).

Fig 2 shows that nickel removal performance was highly unstable. Residual nickel concentrations in the treated effluent varied substantially, from 0.02 ± 0.01 to 60 ± 0.4 mg/L. Multiple episodes of elevated concentrations were recorded, suggesting that the conventional process does not consistently achieve regulatory compliance when the influent nickel loading increases. This significant variability indicates that nickel removal efficiency is highly sensitive to changes in influent characteristics.

**Fig 3:**
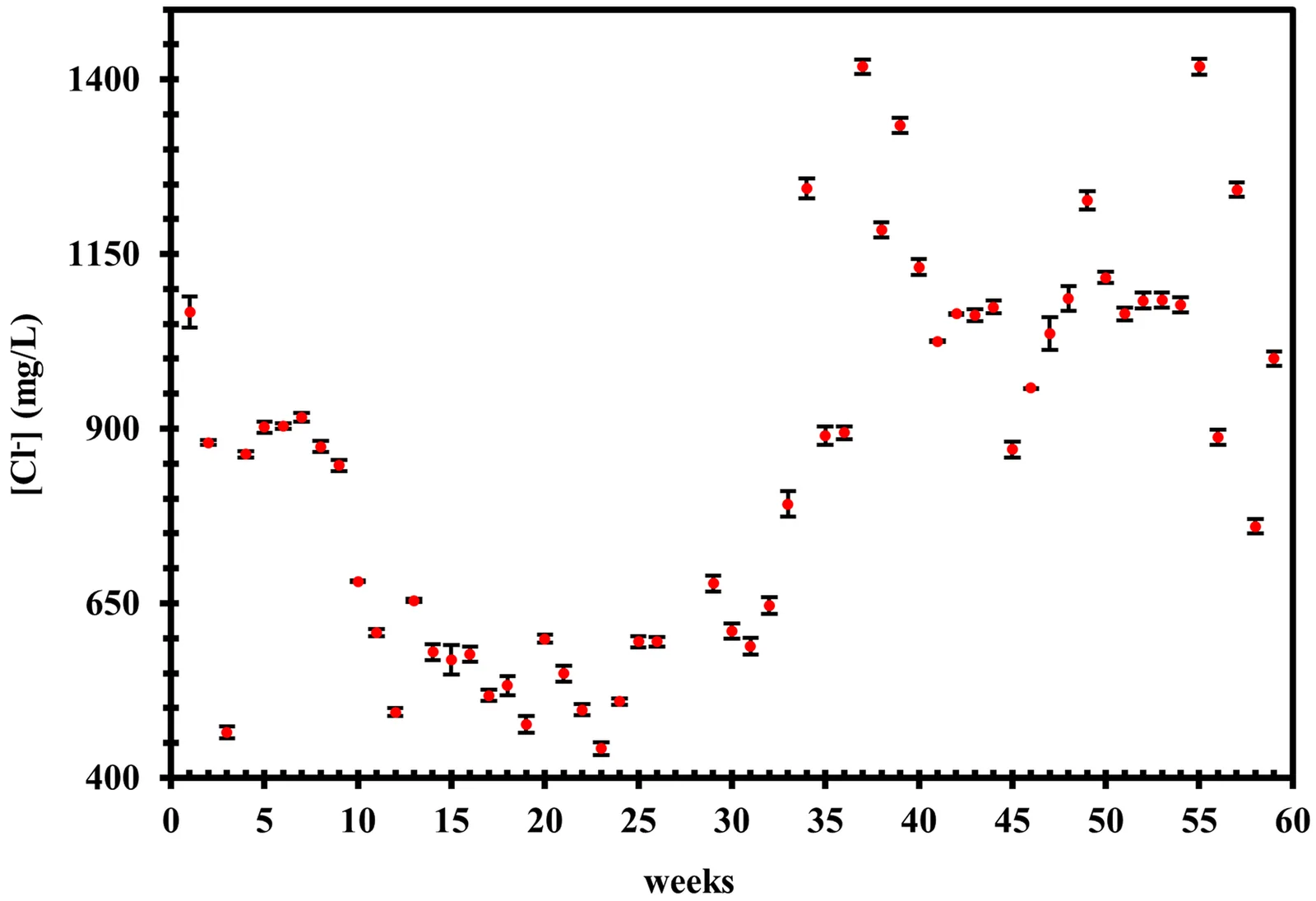
Temporal variations in chloride ion (Cl^−^) concentration in the final treated electroplating wastewater (f-EPW).

Chloride concentration is the primary constraint in conventional treatment. Fig 3 shows that chloride levels in the treated effluent ranged from 442 ± 9–1418 ± 10 mg/L during the monitoring period, frequently exceeding the Tunisian discharge limit of 700 mg/L. This persistent chloride enrichment is primarily due to the extensive use of FeCl_3_ as the principal coagulant [9]. Upon dissolution, FeCl_3_ releases chloride ions (Cl^−^) and to form ferric hydroxhydrolyzeside precipitates, which are essential for coagulation. However, excessive dosing increases chloride loading and generates acidity, consuming alkalinity and lowering pH. These effects lead to operational instability and require additional chemical inputs for pH adjustment, increasing chemical consumption and operating costs (OCs). Furthermore, elevated chloride concentrations restrict the reuse potential of the treated effluent and hinder regulatory compliance.

Long-term monitoring results indicate that, although the conventional treatment process performs adequately under favorable operating conditions, its efficiency is significantly constrained by pronounced temporal variability in EPW composition. These results underscore the limitations of the current treatment strategy and justify developing and evaluating alternative or optimized processes that minimize chloride accumulation and maintain stable, efficient heavy metal removal. This rationale forms the basis for investigating the second treatment process, presented and discussed in the following section.

### 2.3 Methodological framework and treatment configurations

**Fig 4:**
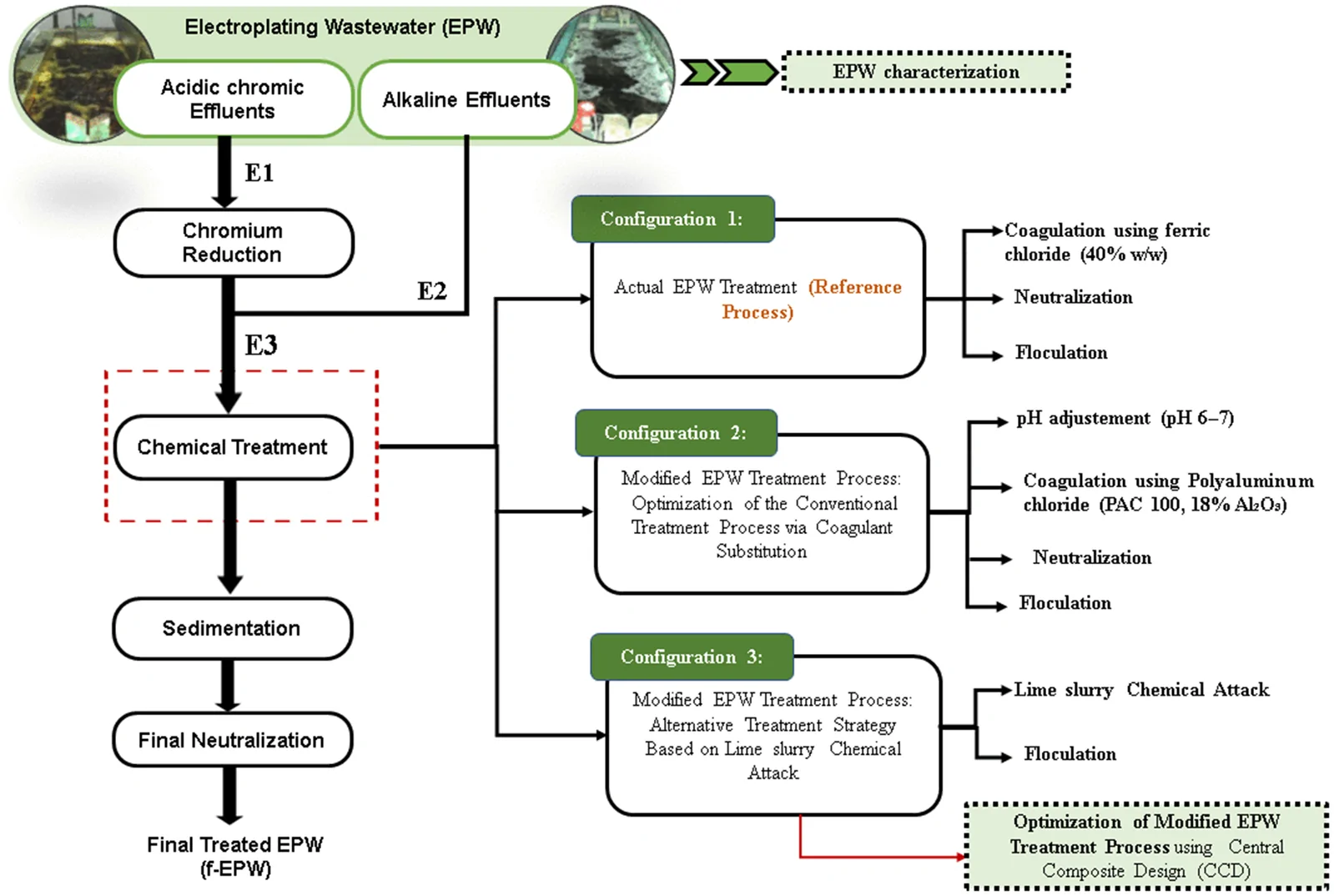
Conceptual framework illustrating the experimental methodology and the treatment configurations evaluated in this study.

Fig 4 illustrates the experimental framework, designed to progressively enhance EPW treatment performance by comparing three successive treatment configurations. Each configuration follows the global PC treatment scheme outlined in Section 2.1 and shown in Fig 4. The same influent reference (E3), representing the mixed alkaline and dechromatized effluent, was consistently used as the initial wastewater stream.

The three treatment processes differ only in the chemical precipitation step, while all upstream and downstream unit operations—including neutralization, solid–liquid separation, sludge dewatering, and final pH adjustment—remain identical. This design enables a direct, consistent comparison of the effects of precipitation chemistry on pollutant removal efficiency, treatment stability, and effluent quality.

- **Configuration 1: Reference conventional precipitation configuration**

In the conventional treatment sequence, ferric chloride is added first to destabilize suspended particles and promote metal complexation. This step is followed by pH adjustment (neutralization) to precipitate as insolubmetalsheavy le hydroxides, such as Ni(OH)_2_ and Cr(OH)_3_. Premature pH elevation can cause uncontrolled precipitation and hinder effective coagulant dispersion. This sequence was maintained because it reflects the treatment protocol currently used at the industrial facility examined in this study. Adjusting pH after coagulation ensures that metal hydroxide precipitation occurs under controlled conditions and does not interfere with coagulation chemistry.

- **Configuration 2: Enhanced precipitation process with optimized coagulation**

To address the limitations of the reference configuration while preserving the overall treatment structure, a modified process (Configuration 2) was developed. In this configuration, FeCl_3_ was replaced with PAC as the coagulant, and pH was adjusted before the precipitation step to optimize PAC hydrolysis speciation. This approach leverages PAC’s strong pH dependence, which is most effective within a narrow pH range. After coagulation, a final pH adjustment (neutralization to pH 8.0–8.6) was performed to complete metal hydroxide precipitation and ensure regulatory compliance. The primary objective of this modification was to reduce chloride input from chemical addition while improving the efficiency of pollutant destabilization and aggregation. The overall treatment layout remained consistent with Configuration 1.

- **Configuration 3: Chemical attack by lime slurry (LS)**

A second alternative configuration, referred to as Configuration 3, was implemented by replacing the conventional coagulation-based precipitation step with a lime slurry (LS)–based chemical process. In this approach, lime (Ca(OH)_2_) serves as both the coagulant and the pH-adjusting agent. Direct addition of LS raises the pH in the precipitation zone (pH 6.5–9), resulting in the simultaneous formation and aggregation of metal hydroxide flocs in a single step. This approach eliminates the need for sequential pH adjustment and coagulant addition, thereby simplifying the process and enhancing overall performance.

After implementation, Configuration 3 was selected for further optimization using RSM in combination with a Central Composite Design (CCD) to determine optimal operating conditions and maximize treatment performance.

Overall, this comparative methodological framework provides a systematic basis for evaluating precipitation strategies, thereby supporting the selection and optimization of the most efficient precipitation treatment configuration.

### 2.4 Analytical methods

The physicochemical (PC) parameters used to evaluate EPW performance in this study were systematically analyzed using standardized, validated methods.

pH was measured with a calibrated digital pH meter (AZ Instrument, model 86501) following APHA Method 4500-H ⁺ B [APHA, 2017]. Electrical conductivity (ECw, µS/cm), an indicator of wastewater ionic strength and total dissolved salts, was determined with a benchtop conductivity meter (Eutech Instruments, model CON2700) following APHA Method 2510B [APHA, 2017].

Total suspended solids (TSS) were quantified using the standard gravimetric method in accordance with APHA guidelines (APHA, 2017) [34]. Samples were filtered through pre-weighed membrane filters, then dried at 105°C and reweighed to determine suspended particulate concentrations.

Chemical oxygen demand (COD) was measured using the dichromate reflux method (ISO 15705) [35], which quantifies the total organic matter oxidizable by dichromate in wastewater. Biochemical oxygen demand over five days (BOD₅) was determined by the standard dilution and incubation procedure (APHA, 2017) [34], providing an estimate of the biodegradable organic load.

Chloride ions (Cl^−^, mg/L) were usingtitration argentometric by analyzed Mohr’s method (APHA et al., 2017) [34]. Heavy metals (HMs), including chromium (Cr(III)), nickel (Ni), copper (Cu), zinc (Zn), iron (Fe), and lead (Pb), were quantified by inductively coupled plasma atomic emission spectroscopy (ICP-AES, Activa-HORIBA JOBIN YVON).

This analytical technique enabled accurate determination of trace metal concentrations, which serve as critical indicators for assessing EPW contamination and treatment efficiency.

### 2.5 Operating Cost (OC) analysis

Using the comparative methodological framework, an OC analysis was conducted to evaluate the economic performance of the three PC treatment configurations (Processes 1, 2, and 3). To ensure consistency and comparability, the assessment focused exclusively on the chemical operating cost (*C*_chem_, €·m^−3^), which reflects the consumption of chemical reagents directly attributable to each configuration.

All three configurations operated under identical hydraulic and operational conditions, including treatment capacity, mixing intensity, and hydraulic residence time. Consequently, the electrical energy demand for pumping and agitation was considered equivalent across configurations and excluded from the comparative analysis. Under these controlled conditions, the primary economic differentiation among the processes stems from the type and dosage of chemical reagents used.

Emphasizing *C*_chem_ provides a rigorous and meaningful basis for comparing the relative economic performance of the three treatment configurations without introducing bias related to infrastructure or energy consumption [1].

*C*_chem_ was calculated using Equation (1).


$$\begin{array}{c} C_{chem}=\sum_{i}{\left(D_{i}xP_{i}\right)}_{i} \end{array}$$ (1)

In this context, *i* denotes the chemical reagents in each configuration: FeCl_3_, sulfuric acid (H_2_SO_4_), antifoaming agent (AF), and flocculant for Configuration 1; PAC 100, H_2_SO_4_, AF, and for Configuration 2flocculant; and LS, AF, and flocculant for Configuration 3. *D*_i_ (kg·m^−3^) is the dosage of reagent *i*, and *P*_i_ (€/kg) is its unit price.

### 2.6 Experimental setup and operational parameters for optimizing the treatment process (Configuration 3)

**Fig 5:**
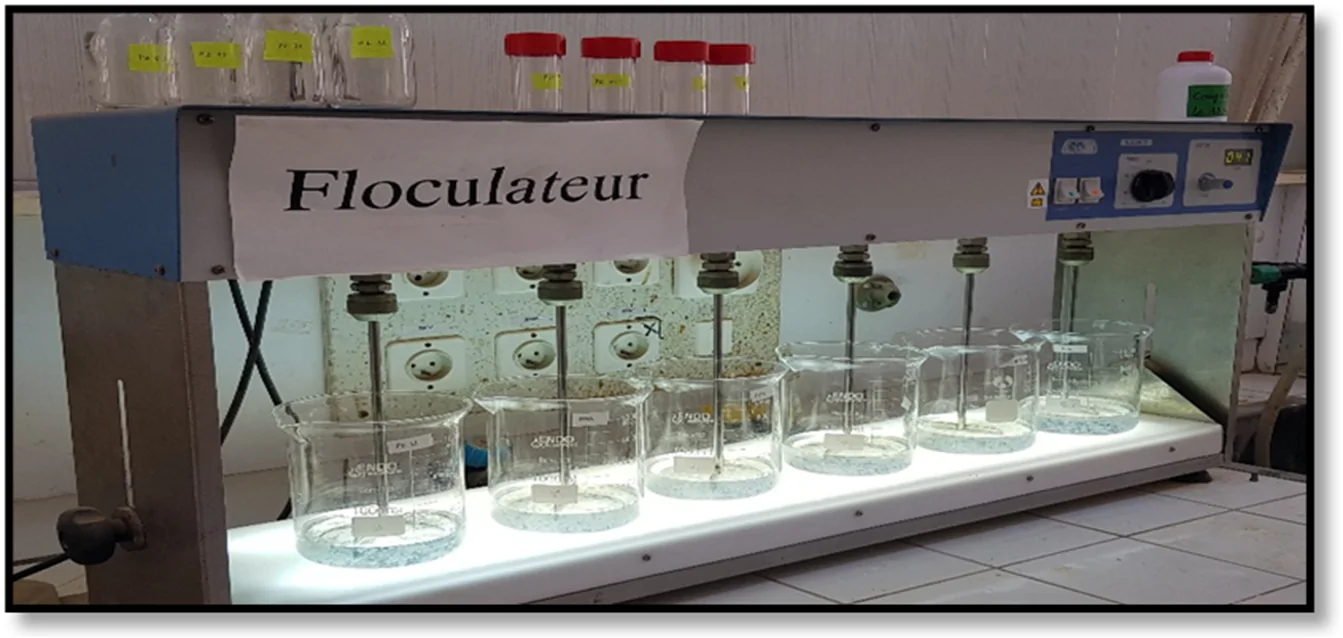
Experimental jar-test apparatus and instrumentation.

Configuration 3 was optimized using a standard jar-test apparatus with six programmable paddle mixers and parallel 1-L beakers (Fig 5). Three primary operational parameters were systematically evaluated: initial nickel concentration ([Ni]ᵢ) in the E3 effluent, initial pH (adjusted with LS), and flocculant dosage (anionic polyacrylamide, [A-PAM]). LS was selected as the neutralizing agent for its demonstrated effectiveness in heavy-metal precipitation and cost-effectiveness in industrial-scale EPW treatment [36,37].

For each experimental run, 300 mL of E3 effluent was dispensed into six parallel jars, and predetermined doses of LS and flocculant were added according to the experimental design matrix. The treatment sequence comprised two main stages. First, neutralization was conducted at 64 rpm for 5 minutes to ensure pH adjustment, complete reagent dispersion, and floc formation. During this stage, the relatively high rotational speed generated turbulent flow, which maximized diffusion-driven transport of coagulant species toward metal-bearing colloidal and complexed phases, thereby increasing the collision frequency between destabilized particles and facilitating initial microfloc formation [38]. This was followed by slow flocculation at 41 rpm for 15 minutes, during which the reduced mixing intensity shifted the hydrodynamic regime toward laminar conditions favorable for orthokinetic aggregation. This allowed growing flocs to collide and consolidate without being subjected to disruptive shear forces that could cause floc breakage [38]. The second stage involved quiescent settling for 30 minutes, during which the absence of mechanical energy input enabled gravitational separation of mature floc structures while preserving their physical integrity. This progressive reduction in mixing intensity reflects a deliberate balance between maximizing mass transfer efficiency during the early coagulation stage and preserving floc structural stability during subsequent growth and separation stages, both of which directly influence the final treated effluent quality.

The operational parameters, specifically mixing speeds and durations, were aligned with the current industrial treatment process to enable direct comparison and practical application. After sedimentation, supernatant samples were collected from each jar to measure residual pollutant concentrations, including nickel, chromium, COD, and other specified parameters. All experiments were conducted at ambient temperature (20–25°C) and performed in triplicate to ensure statistical reliability.

### 2.7 Response Surface Methodology (RSM) model

#### 2.7.1 Preliminary trials: pH adjustment and metal hydroxide precipitation behavior.

Preliminary experiments investigated pH-adjustment behavior and the characteristics of metal hydroxide precipitation in EPW ([Ni]ᵢ = 92 mg/L). The presence of chromium significantly affects nickel precipitation behavior in the bimetallic system.

**Fig 6:**
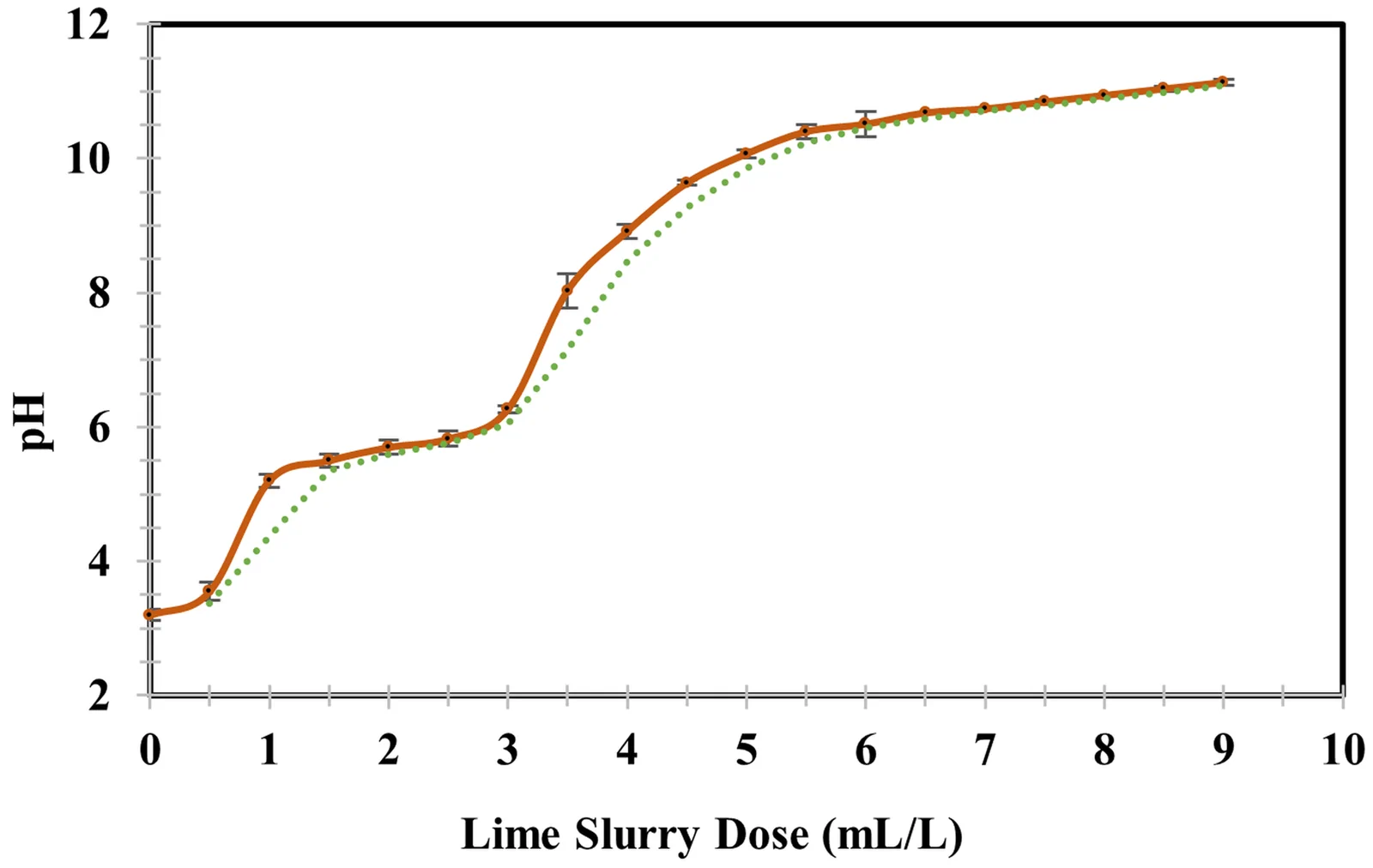
Variation in pH with Lime Slurry (LS) dosage during preliminary neutralization experiments with electroplating wastewater (EPW).

Although previous studies report individual precipitation optima at pH 6.4 for Cr(III) and pH 8.5 for Ni(II) [36], the titration curve (Fig 6) shows that complete neutralization and alkaline conditions (pH 9–10) occur at LS dosages above 5 mL/L (400 mg/L, based on an LS concentration of 80 g/L). This pH range enables simultaneous precipitation of both metal hydroxides, as indicated by the pH-stabilization plateau. The steep transition zone between 3–5 mL/L (240–400 mg/L), corresponding to pH 6–9, marks the equivalence point, where buffering capacity is minimal and pH control is critical. Therefore, maintaining pH values above 9 with an adequate lime dosage is essential to maximize metal removal efficiency in the bimetallic Cr-Ni system.

#### 2.7.2 Experimental design.

A CCD was implemented within the RSM framework to systematically evaluate how key operational parameters affect nickel removal efficiency from EPW. CCD is a well-established second-order experimental design that estimates quadratic and interaction effects while minimizing the number of experimental runs, making it particularly suitable for process optimization studies. In this study, experimental design, data analysis, and model development were performed using Design-Expert® software.

Based on comparative experimental results, the most effective of the three wastewater treatment processes previously investigated was selected for further optimization.

After determining the optimal treatment process, three independent variables were selected for optimization based on preliminary experiments and supporting literature. These variables, [Ni]ᵢ, solution pH, and flocculant dosage ([A-PAM]), were identified as the primary factors influencing nickel hydroxide precipitation, floc formation, settling behavior, and overall purification performance [26,37].

A-PAM was selected as the for itsflocculant strong affinity for Ni(OH)_2_ precipitates and its demonstrated effectiveness in treating EPW [39]. Flocculant dosage was systematically varied using a CCD to assess its effects on nickel removal efficiency and floc settling performance.

The experimental ranges for the selected variables were established using preliminary results and datasets from the electroplating plant. [Ni]ᵢ, representing the nickel concentration measured immediately after the dechromatization step, ranged from 33 to 92 mg/L. This interval was chosen to reflect the annual variability of post-dechromatization nickel concentrations (E3) documented in the industrial monitoring database. Accordingly, the selected interval reflects realistic operating conditions and enhances the practical relevance of the experimental design. The pH range and [A-PAM] concentrations were determined based on preliminary tests and literature data on PAM application in EPW treatment [26,39]. These ranges were chosen to include both suboptimal and near-optimal operating conditions, facilitating the identification of significant linear, quadratic, and interaction effects. The primary response variable was the residual nickel concentration in the treated effluent. Table 2 summarizes the experimental domain and the corresponding coded levels of the independent variables used in the CCD.

**Table 2:** Experimental ranges and levels of independent variables used in the Response Surface Methodology (RSM). (A-PAM: anionic polyacrylamide; [Ni]_i_: nickel concentration prior to treatment.).

Variable	-*α*	−1	0	+1	+*α*
A: [Ni]_i_ (mg/L)	33	45	62.5	80	92
B: pH	6.32	7	8	9	9.68
C: [A-PAM] (mg/L)	2.6	4	6	8	9.36

## 3. Results and discussion

### 3.1 Comparative evaluation of treatment configurations across successive operating periods and assessment of process stability

#### 3.1.1 Quality of final treated electroplating wastewater (f-EPW) and pollutant removal efficiency.

**Fig 7:**
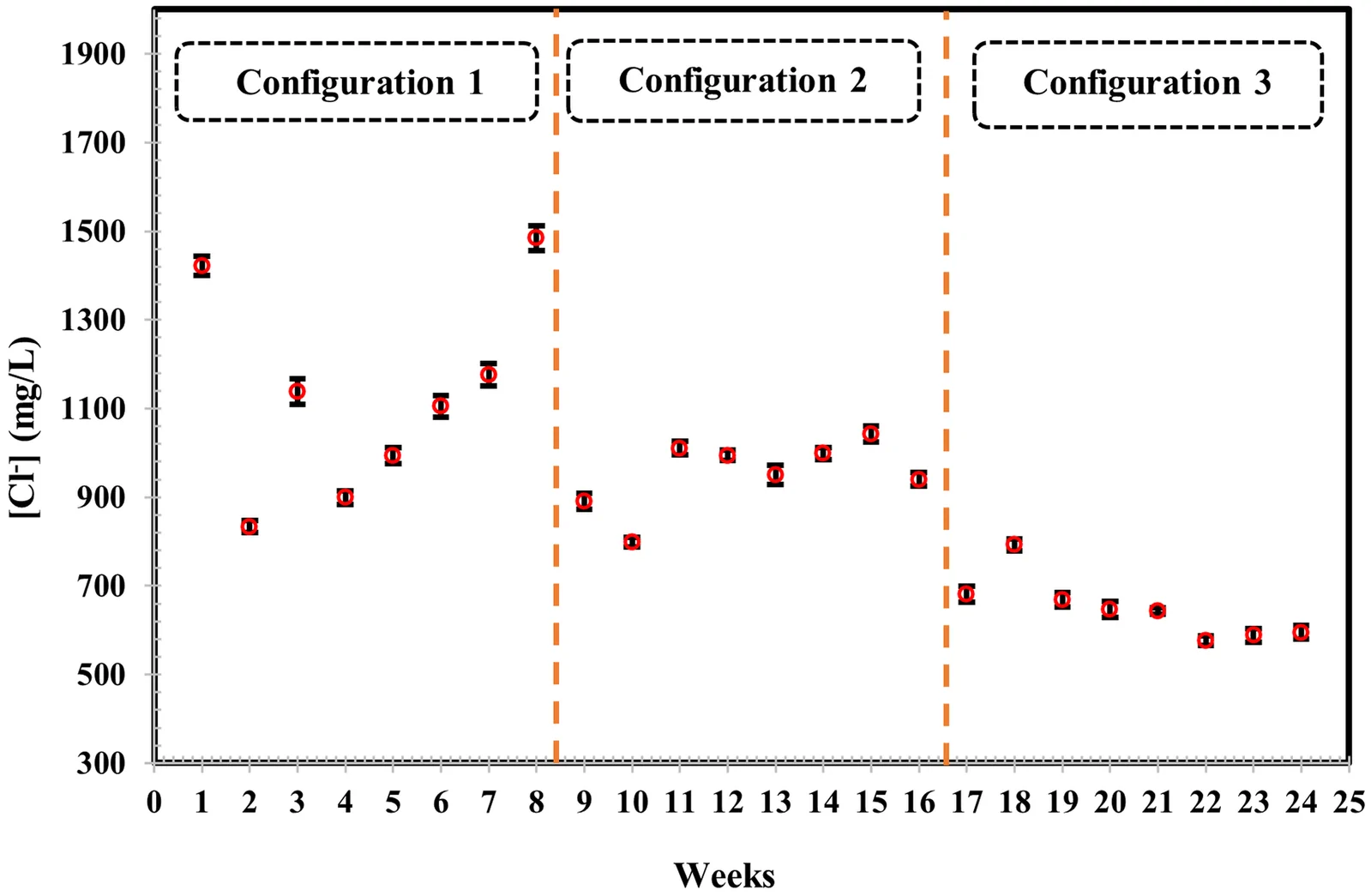
Chloride concentration in the final treated electroplating wastewater (f-EPW) indicates process stability over 24 weeks.

**Fig 8:**
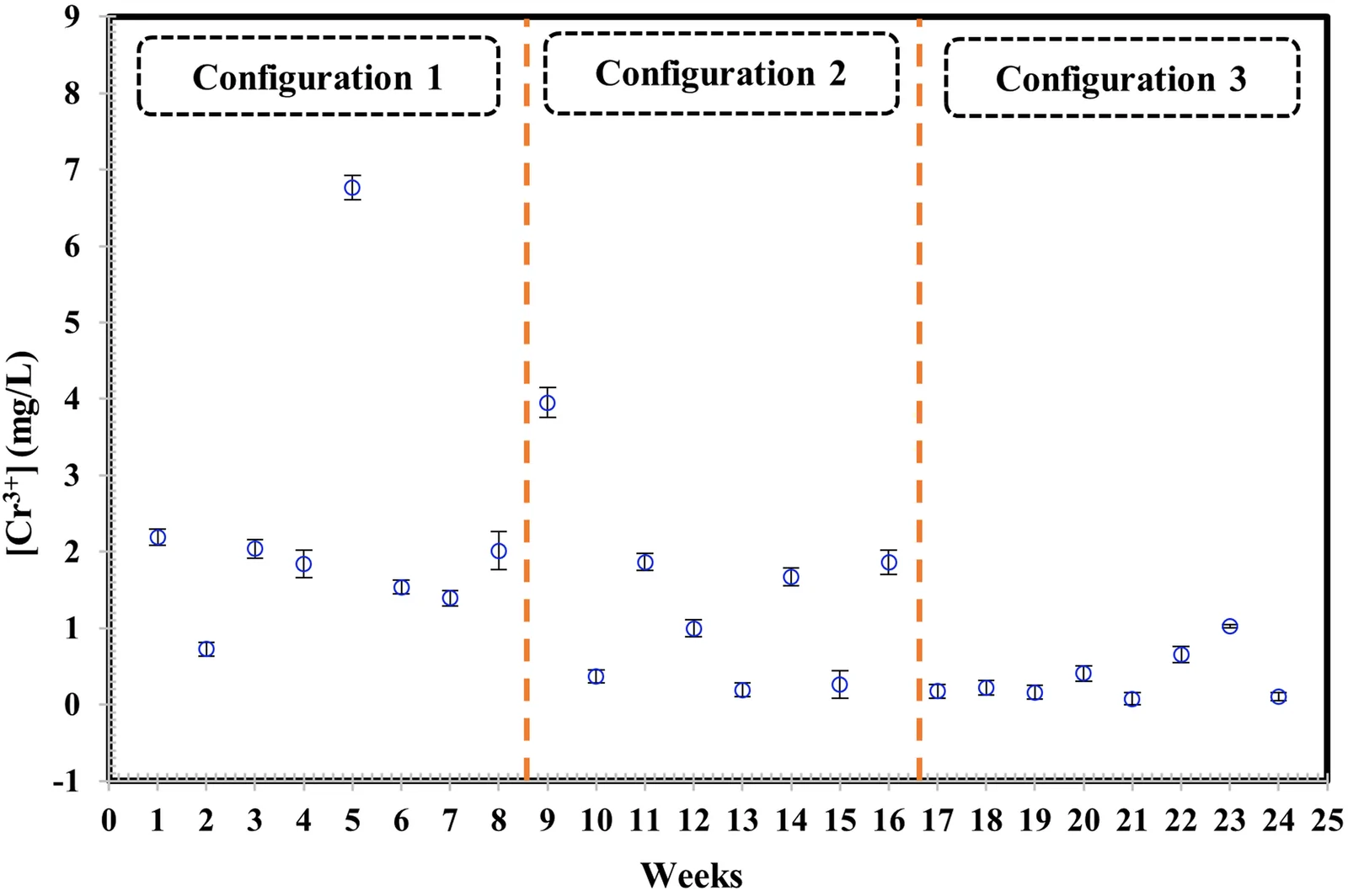
Chromium concentration in the final treated electroplating wastewater (f-EPW) indicates process stability over 24 weeks.

**Fig 9:**
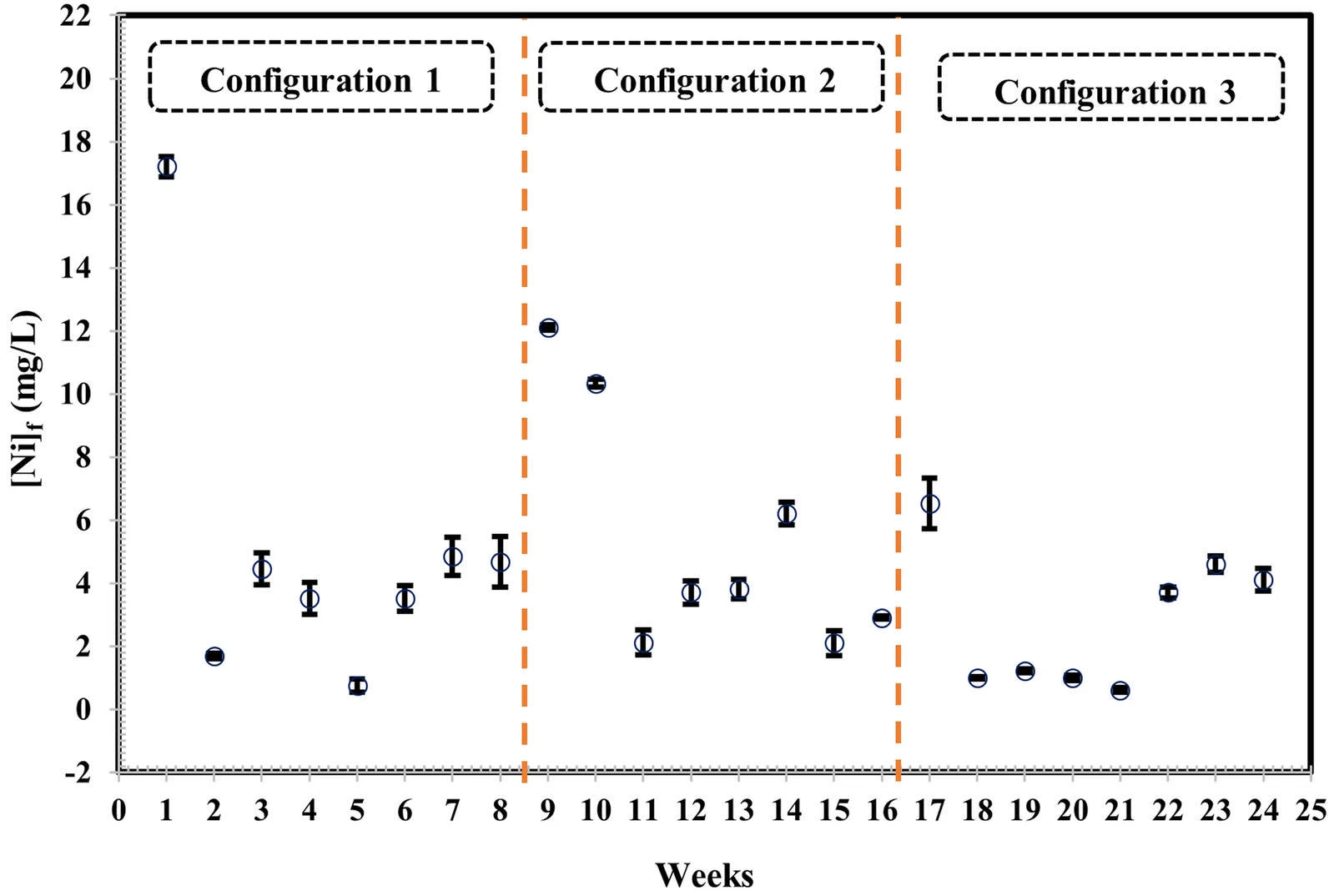
Nickel concentration in the final treated electroplating wastewater (f-EPW) indicates process stability over 24 weeks.

Long-term stability is a key performance criterion for industrial wastewater treatment processes because these systems must operate reliably despite temporal fluctuations in the influent composition [1]. Figs 7–9 present the 24-week monitoring results for chloride, chromium, and nickel concentrations in the treated effluents of the three configurations, with post-dechromatization wastewater (E3) serving as the reference influent.

Before interpreting the experimental results, it is necessary to consider the chemical speciation of metals in the E3 effluent matrix. This matrix is significantly more complex than a simple metal-hydroxide system because it contains strong chelating agents commonly found in electroplating bath formulations, including ethylenediaminetetraacetic acid (EDTA), gluconates, citrates, and various surfactants. These agents form thermodynamically stable organometallic complexes with nickel and chromium. A substantial portion of these metals remains in solution and is not readily available for hydroxide precipitation, which accounts for discrepancies between theoretical and experimentally observed residual metal concentrations. The coagulants used in this study influence the dissociation of these complexes in distinct ways. LS acts through both competitive calcium-ion binding to EDTA and sustained alkalinity supply, thereby shifting equilibria toward precipitation. In contrast, ferric chloride releases chelated metals via the competitive affinity of Fe³⁺ for EDTA. Still, this process results in the formation of soluble iron-EDTA complexes that are resistant to further precipitation [40].

Chloride management varied considerably among the three configurations, underscoring the direct impact of coagulant selection on effluent chloride loading. In Configuration 1, effluent chloride concentrations ranged from 800 to 1500 mg/L and showed high temporal variability throughout the monitoring period, consistently exceeding the NT 106.002 discharge limits. This performance is primasuboptimalrily attributed to the use of FeCl_3_ as a coagulant, which, upon dissolution and hydrolysis, releases additional Cl^−^ into the treated effluent, thereby increasing the chloride load rather than mitigating it [41,42].

Configuration 2 showed a marked improvement over Configuration 1, with effluent chloride concentrations stabilizing between 800 and 1100 mg/L and exhibiting greater temporal consistency. Replacing FeCl_3_ with polyaluminum chloride (PAC) eliminated the secondary chloride input associated with iron-based coagulation, thereby reducing the overall effluent chloride load. Although PAC contains chloride in its chemical structure, its contribution per unit dose is significantly lower than that of FeCl_3_, which explains the partial improvement observed [43]. However, actual Cl^−^ removal through incorporation into precipitated solids remains negligible, as chloride ions do not form insoluble hydroxide or carbonate phases under the prevailing treatment conditions.

The most substantial improvement occurred with Configuration 3, where effluent chloride concentrations progressively decreased and stabilized at 550–750 mg/L during the final monitoring phase, particularly in weeks 17–24. This represents an approximate 50% reduction compared to the effluent levels of Configuration 1. However, this reduction does not indicate true Cl^−^ precipitation or removal from the aqueous phase, as chloride ions remain soluble under all physicochemical treatment conditions and are not subject to hydroxide co- precipitation [41]. Instead, the observed reduction is primarily due to the complete elimination of FeCl_3_ as a secondary chloride source. At the coagulant dosage used in Configuration 1 (0. 864 kg/m ³), FeCl_3_ hydrolysis releases an estimated 530 mg Cl^−^/L into the treated effluent. This substantial secondary chloride load is entirely avoided in Configuration 3 by substituting FeCl_3_ with chloride- free lime slurry (Ca(OH)_2_).

Although chloride removal showed clear efficiency gains across the tested configurations, chromium removal was more variable in stability than in average efficiency. Configurations 1 and 2 exhibited substantial variability, with concentration peaks of 2 to 2.5 mg/L during weeks 1–2 for Configuration 1 and fluctuating values that occasionally exceeded 2 mg/L for Configuration 2, particularly during weeks 9–11. In contrast, Configuration 3 consistently maintained low chromium concentrations, predominantly below 1 mg/L during weeks 17–24, with only occasional measurements approaching 1 mg/L. The sequential acidification and neutralization strategy offers enhanced control over chromium speciation and precipitation kinetics [44].

Among the three target pollutants, nickel removal posed the greatest challenge due to the high residual concentration after dechromatization. Configurations 1 and 2 showed pronounced variability, similar to their chromium removal patterns, with effluent concentrations ranging from approximately 2 mg/L to peaks of 12 mg/L in Configuration 2 at week 11 and 17 mg/L in Configuration 1 during week 1. This instability highlights the limited buffering capacity of the conventional coagulation system.

Consistent with trends observed for chloride and chromium, process configuration 3 showed improved performance in reducing the final residual nickel concentration ([Ni]_f_), with effluent concentrations typically ranging from 1 to 5 mg/L. Occasional increases reached approximately 6.5 mg/L, particularly in week 17. During the stable operating period from weeks 19–24, concentrations often approached or fell below 1 mg/L, supporting the effectiveness of lime-based precipitation with optimized pH control [44]. However, residual fluctuations suggest that this configuration remains somewhat sensitive to influent variability, though less so than conventional methods.

The consistent superiority of Configuration 3 across all monitored parameters stems from several synergistic factors. Lower standard deviations for chromium and nickel indicate enhanced operational resilience, attributable to the buffering capacity of excess lime, accelerated hydroxide precipitation kinetics, and the formation of more stable metal hydroxide phases rather than the metal–coagulant complexes observed in conventional systems [45]. These factors yield higher average removal efficiencies and significantly reduced temporal variability.

Despite these advantages, all three configurations share a fundamental limitation: reliance on fixed chemical dosing strategies. Variability in nickel concentrations, driven by changes in electroplating operations, rinsing practices, and dechromatization efficiency, cannot be effectively managed under static operating conditions [1]. As a result, periods of under- or over-dosing are unavoidable, negatively affecting both treatment consistency and chemical consumption efficiency. This limitation is especially pronounced during the initial weeks of operation for each configuration, when adaptation to influent characteristics requires gradual optimization.

In summary, Configuration 3 demonstrates the highest effectiveness among current treatment strategies, achieving approximately 50% chloride removal, over 99% chromium removal with exceptional stability during the final monitoring period, and approximately 95–97% nickel removal, all with enhanced stability under realistic industrial conditions. To ensure consistently robust performance under highly variable influent conditions, this configuration should be integrated with advanced control strategies, such as real-time metal monitoring and adaptive dosing systems [46]. This process intensification would enable dynamic adjustment of lime dosage and pH set-points, thereby transforming Configuration 3 from a superior static process into a fully adaptive, industrially resilient treatment solution.

#### 3.1.2 Economic feasibility and cost-effectiveness analysis.

The economic feasibility of industrial wastewater treatment processes is a critical criterion for technology selection because OCs directly affect long-term sustainability. OCs typically include energy consumption, chemical reagents, equipment maintenance, and sludge management [1].

In this comparative assessment, all three configurations operated under identical hydraulic conditions and used the same pumping, mixing, and solid–liquid separation equipment. Consequently, energy consumption and routine maintenance were treated as equivalent, making chemical consumption the primary cost-differentiating factor. As summarized in Table 3, the economic comparison focused on the specific chemical operating cost (*C*_chem_, €·m^−3^) per cubic of treated wastmeterewater, which included all reagents such as coagulants, pH adjustment chemicals, antifoaming agents (AF), and polymer flocculant.

**Table 3:** Calculation of chemical operating cost (*C*_chem_). (AF: antifoaming: anionPAM agent, A-ic polyacrylamide, PAC: polyaluminum chloride, LS: lime slurry, *D*_i_: dosage of reagent *i*, *P*_i_: unit price of reagent *i*).

		*P*_i_ (€·kg^−^1)	*D*_i_ (kg·m^−3^)	*C*_chem_ (€·m^−3^)
Configuration 1	FeCl_3_	0.41	0.864	0.432
	H_2_SO_4_	0.21	0.309	
	AF	16.16	3 E-07	
	A-PAM	1.07	0.016	
Configuration 2	PAC100	0.28	0.62	0.303
	H_2_SO_4_	0.21	0.309	
	AF	16.16	3 E-07	
	A-PAM	1.1	0.02	
Configuration 3	LS	0.2	0.59	0.134
	AF	16.16	3 E-07	
	A-PAM	1.07	0,016	

Configuration 1, which used FeCl_3_ coagulation, had the highest operating cost of 0.432 €·m^−3^. This outcome was primarily due to the relatively high unit cost of FeCl_3_ and the substantial dosages required to achieve acceptable treatment performance. Although this conventional approach is widely used in industrial applications, it becomes economically unfavorable for high-strength electroplating wastewater (EPW), where chemical costs account for the majority of operating expenses.

Configuration 2, which uses PAC as the primary coagulant, reduced chemical costs to 0.303 €·m^−3^, representing approximately 30% savings compared to Configuration 1. This reduction is attributed to PAC’s higher coagulation efficiency, which enables lower reagent dosages while maintaining comparable performance. Consequently, Configuration 2 offers a cost-effective intermediate solution that requires minimal process modifications.

Process Configuration 3, which uses lime-driven chemical attack, achieved the lowest cost at 0.134 €·m^−3^, corresponding to approximately 69% and 56% reductions compared to Configurations 1 and 2, respectively. This economic advantage stems from lime’s multifunctional role, which enables pH adjustment, metal precipitation, and coagulation, thereby eliminating the need for multiple chemical inputs. Furthermore, the wastewater’s intrinsic acidity was sufficient for the chemical attack stage, eliminating the need for supplementary acid. These findings indicate that optimized reaction pathways can provide both enhanced treatment efficiency and significant cost savings.

In addition to direct chemical costs, Configuration 3 offers further economic advantages in sludge management. The literature indicates that coagulation-flocculation processes generate sludges with higher volumes, poorer dewaterability, and lower quality than those from lime precipitation [41].

Sludges from coagulant-based processes contain substantial residual polymers and metal-organic complexes, increasing volume and complicating disposal [42]. In contrast, lime precipitation primarily produces crystalline metal hydroxide sludges that settle and dewater more readily, yielding lower dewatered cake volumes and lower transportation and disposal costs [41,44].

Furthermore, lime sludge (LS) has greater potential for recycling because metal hydroxides can be efficiently recovered via acidification or thermal treatment [20,38]. Empirical studies confirm that lime precipitation yields more stable sludge with reduced heavy metal (HM) leaching potential, mitigating environmental risks and lowering regulatory compliance costs [44,45].

The nickel-rich sludge produced by Configuration 3 has significant valorization potential, as the recovered nickel can serve as a catalyst in hydrogen production [47]. Moreover, eliminating chloride-based coagulants reduces equipment corrosion, lowering maintenance costs and extending service life [48].

Configuration 3 is the most cost-effective treatment option, delivering the lowest operating costs and stable, superior performance. The substantial savings, approaching 70% compared with conventional coagulation, make a strong economic case for upgrading electroplating facilities’ processes. These findings demonstrate that regulatory compliance, environmental performance, and economic competitiveness can be achieved simultaneously through well-designed treatment strategies, thereby supporting the transition to more sustainable industrial wastewater management practices.

### 3.2 Optimization of treatment process configuration 3

#### 3.2.1 Application of response surface methodology (RSM) and model development.

Configuration 3 was optimized using RSM with a CCD to systematically evaluate how key operational parameters interact to influence nickel removal efficiency. The experimental design matrix, shown in Table 4, includes three independent variables: initial nickel concentration ([Ni]ᵢ) in the E3 effluent, solution pH, and anionic polyacrylamide concentration ([A-PAM]).

**Table 4:** Experimental design matrix and corresponding responses ([Ni]_i_: nickel concentration before treatment; [A-PAM]: anionic polyacrylamide dosage; [Ni]_f_: nickel concentration after treatment).

Run	A: [Ni]_i_ (mg/L)	B: pH	C: [A-PAM] (mg/L)	A	B	C	[Ni]_f_ (mg/L)	
							Actual value	Predicted value
1	−1	−1	−1	45	7	4	8.61	9.51
2	1	−1	−1	80	7	4	12.53	12.14
3	−1	1	−1	45	9	4	0.60	0.12
4	1	1	−1	80	9	4	0.37	0.35
5	−1	−1	1	45	7	8	7.64	7.44
6	1	−1	1	80	7	8	9.50	9.76
7	−1	1	1	45	9	8	0.93	0.99
8	1	1	1	80	9	8	2.02	0.90
9	−1.68	0	0	33	8	6	0.56	0.22
10	1.68	0	0	92	8	6	1.70	2.35
11	0	−1.68	0	62.5	6.318	6	20.3	19.85
12	0	1.68	0	62.5	9.682	6	3.76	4.52
13	0	0	−1.68	62.5	8	2.6	2.01	1.90
14	0	0	1.68	62.5	8	9.36	0.20	0.62
15	0	0	0	62.5	8	6	0.93	0.92
16	0	0	0	62.5	8	6	0.93	0.92

Experimental data from CCD trials were analyzed using regression to develop a predictive model linking operational parameters to the final nickel concentration ([Ni]_f_) in the treated effluent. Statistical analysis and model development were performed using Design-Expert software. The relationship between the independent variables and the response is described by a second-order polynomial equation that shows significant curvature and interaction effects within the experimental range. Equations (2) and (3) present the model in coded and actual terms, respectively, enabling both comparative assessment of factors and practical application in process control.


$$\begin{array}{cc} & 𝐂𝐨𝐝𝐞𝐝\;𝐟𝐨𝐫𝐦:\;𝐘=\text{0}.\text{5859}+\text{1}.\text{07A}-\text{4}.\text{2B}-\text{0}.\text{3301C}-\text{1}.\text{34AB}+\text{0}.\text{1783AC}+\text{0}.\text{735BC}+{\text{0}.\text{6423A}}^{\text{2}}\\ & +{\text{3}.\text{98B}}^{\text{2}}+{\text{0}.\text{1205C}}^{\text{2}} \end{array}$$ (2)

$$\begin{array}{cc} & Actual\;form:\;{\left[Ni\right]}_{𝐟}=\text{293}.\text{49}+{\text{0}.\text{273}[\text{Ni}]}_{\text{i}}-\text{68}.\text{34pH}-\text{3}.\text{35}[\text{A}-\text{PAM}]-{\text{0}.\text{034}[\text{Ni}]}_{\text{i}}\cdot \text{pH}-\\ & {\text{0}.\text{002}[\text{Ni}]}_{\text{i}}\cdot [\text{A}-\text{PAM}]+\text{0}.\text{368pH}\cdot [\text{A}-\text{PAM}]+{\text{0}.\text{000422}[\text{Ni}]}_{\text{i}}^{\text{2}}+{\text{3}.\text{98pH}}^{\text{2}}+{\text{0}.\text{030}[\text{A}-\text{PAM}]}^{\text{2}} \end{array}$$ (3)

In this context, Y denotes the coded response, [Ni]_f_ is the final nickel concentration (mg/L), [Ni]ᵢ is the initial nickel concentration (mg/L), pH is the solution pH, and [A-PAM] is the anionic polyacrylamide dosage (mg/L). The coded equation enables direct comparison of factor effects by normalizing the coefficients to dimensionless units. In contrast, the actual equation provides practical predictions of effluent quality under defined operating conditions.

Coefficient analysis clarifies how factors influence nickel removal. The large negative pH coefficient (−4.20 in coded form) shows that increasing pH within the experimental range substantially reduces [Ni]_f_, consistent with the pronounced decrease in Ni(OH)_2_ solubility from pH 7–9. Thermodynamically, each unit increase in pH decreases the equilibrium Ni² ⁺ concentration by about two orders of magnitude, a trend qualitatively captured by this coefficient. The positive quadratic pH coefficient (3.98**)** produces a response surface minimum and reflects the established re-dissolution of Ni(OH)_2_ at very high pH, attributed to the formation of soluble [Ni(OH)_3_]^−^ and [Ni(OH)_4_]^2−^ hydroxo-complexes [32]. The inflection point near pH 9–10, consistent with published solubility diagrams, indicates an optimal pH beyond which further increases in pH may reduce removal efficiency, likely due to hydroxide resolubilization under highly alkaline conditions or the formation of soluble hydroxo complexes [32]. The negative AB interaction coefficient (−1.34) suggests that the beneficial effect of pH on nickel removal diminishes at higher [Ni]ᵢ, in agreement with precipitation kinetics. At elevated [Ni²⁺], the molar stoichiometric demand for OH^−^ increases, necessitating higher pH to maintain the same driving force for nucleation and growth of Ni(OH)_2_ crystals, rather than merely reflecting saturation of precipitation capacity or kinetic limitations under increased metal loading. The positive linear [Ni]ᵢ coefficient (+1.07) indicates that higher influent concentrations yield higher residual concentrations, consistent with mass-balance constraints near the precipitation capacity. The positive BC interaction coefficient (+ 0.735) indicates that A- PAM flocculation is more effective when Ni(OH)_2_ precipitates are fully formed (pH ≥ 8.5), as the anionic polymer bridges positively surface-charged hydroxide colloids. At low pH, incomplete precipitation produces fewer bridgeable particles, thereby limiting the flocculant’ s contribution to nickel removal.

#### 3.2.2 Statistical analysis and model validation.

Analysis of variance (ANOVA) was conducted to assess model significance, adequacy, and predictive capability. The results, presented in Table 5, indicate strong model performance, with an *F*-value of 111.37 (*p*-value < 0.0001). This result indicates that the likelihood of obtaining such a large F-value due to random noise is less than 0.01%. Therefore, the developed model effectively captures the relationships between operational parameters and nickel removal performance rather than reflecting random experimental variation.

**Table 5:** Results of analysis of variance (ANOVA). Abbreviations: MS, mean square; DF, desirability function; *p*-value, probability value; *F*-value, Fisher value.

Source	DF	MS	*F*-value	*p*-value	
Model	9	56.75	111.37	< 0.0001	Significant
A	1	1.23	2.40	0.1596	Not Significant
B	1	149.11	292.64	< 0.0001	Significant
C	1	0.9219	1.81	0.2155	Not Significant
AB	1	2.90	5.70	0.0440	Significant
AC	1	0.0512	0.1005	0.7594	Not Significant
BC	1	4.32	8.48	0.0195	Significant
A^2^	1	0.2116	0.4153	0.5373	Not Significant
B^2^	1	200.68	393.85	< 0.0001	Significant
C^2^	1	0.1837	0.3604	0.5649	Not Significant

Analysis of individual term significance indicated that pH (B) had the greatest influence on nickel removal, as evidenced by an *F*-value of 292.64 (*p*-value < 0.0001). This pronounced effect underscores the pivotal role of pH in controlling the equilibria of nickel hydroxide precipitation, with solubility minima observed within defined pH intervals. The quadratic pH term (B²) was also highly significant (*F*-value = 393.85, *p*-value < 0.0001), confirming notable curvature in the pH-response relationship and suggesting an optimal pH for maximum nickel removal. The interaction terms AB ([Ni]ᵢ × pH, *F*-value = 5.70, *p*-value = 0.044) and BC (pH × flocculant dosage, *F*-value = 8.48, *p*-value = 0.020) were statistically significant, indicating that the effect of pH on nickel removal is modulated by both influent metal concentration and polymer dosing rate. These interactive effects demonstrate the complexity of the treatment system and support the use of RSM over traditional one-factor-at-a-time approaches.

[Ni]ᵢ (A) and flocculant dosage (C) did not show individual statistical significance as main effects (*p*-value > 0.05), indicating that their influence is primarily mediated through interactions with pH rather than acting as independent variables. This finding aligns with established precipitation chemistry, in which pH predominantly governs metal speciation and hydroxide formation [2], while influent concentration and flocculant dosage affect the extent and efficiency of these pH-dependent processes. The lack of significance for certain terms does not preclude their inclusion in the model, as they preserve the hierarchical structure and support the inclusion of significant interaction terms.

Model adequacy was evaluated using several statistical metrics (Table 6). The correlation coefficient (*R*² = 0.9917) indicates that 99.17% of the variability in nickel removal is explained by the model, demonstrating an excellent fit to the experimental data. The adjusted *R*² (0.9832) accounts for the number of model terms and confirms that the high *R*² is not inflated by excessive parameters. The predicted *R*² (0.9352) reflects the model’s predictive performance for new observations. Its close agreement with the adjusted *R*² (difference less than 0.2) suggests the absence of overfitting and supports the model’s robustness for predictive applications beyond the experimental dataset. The adequate precision ratio of 37.08, which substantially exceeds the threshold of 4, indicates a strong signal-to-noise ratio and confirms that the model can reliably distinguish true effects from experimental error across the design space.

**Table 6:** Statistical summary of the developed model (*R*²: correlation coefficient; *R*²_adj_: adjusted *R*²; *R*²_pred_: predicted *R*²).

Response	*R* ^2^	*R*_adj_^2^	*R*_pred_^2^	Adequate precision
[Ni]_f_ (mg/L)	0.9917	0.9832	0.9352	37.0842

**Fig 10:**
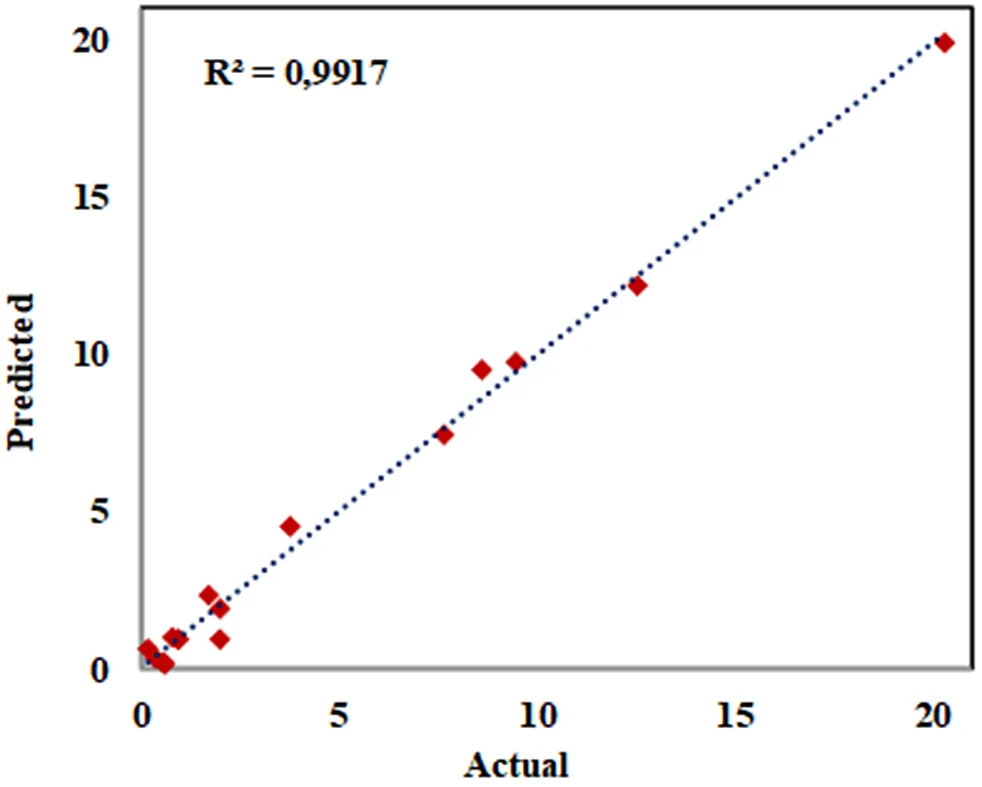
Comparison of predicted and experimental final nickel concentrations [Ni]_f_ (mg/L).

Fig 10 shows a strong correlation between predicted and experimental values, with data points clustering tightly around the diagonal line of perfect prediction, visually confirming the model’s accuracy and the absence of systematic bias.

The rationale for this study stems from the need to address challenges posed by variable influent characteristics in nickel removal processes. Comprehensive statistical analysis shows that the developed RSM model is a reliable tool for improving the performance of Configuration 3. The model shows how different operating conditions affect nickel removal. It provides a clear way to identify the best treatment options that balance removal rates, chemical use, and process stability, even as incoming water changes. This approach addresses earlier problems by using fixed settings to help users make data-driven choices and adapt to real-time changes in wastewater.

#### 3.2.3 Interactive effects visualization.

**Fig 11:**
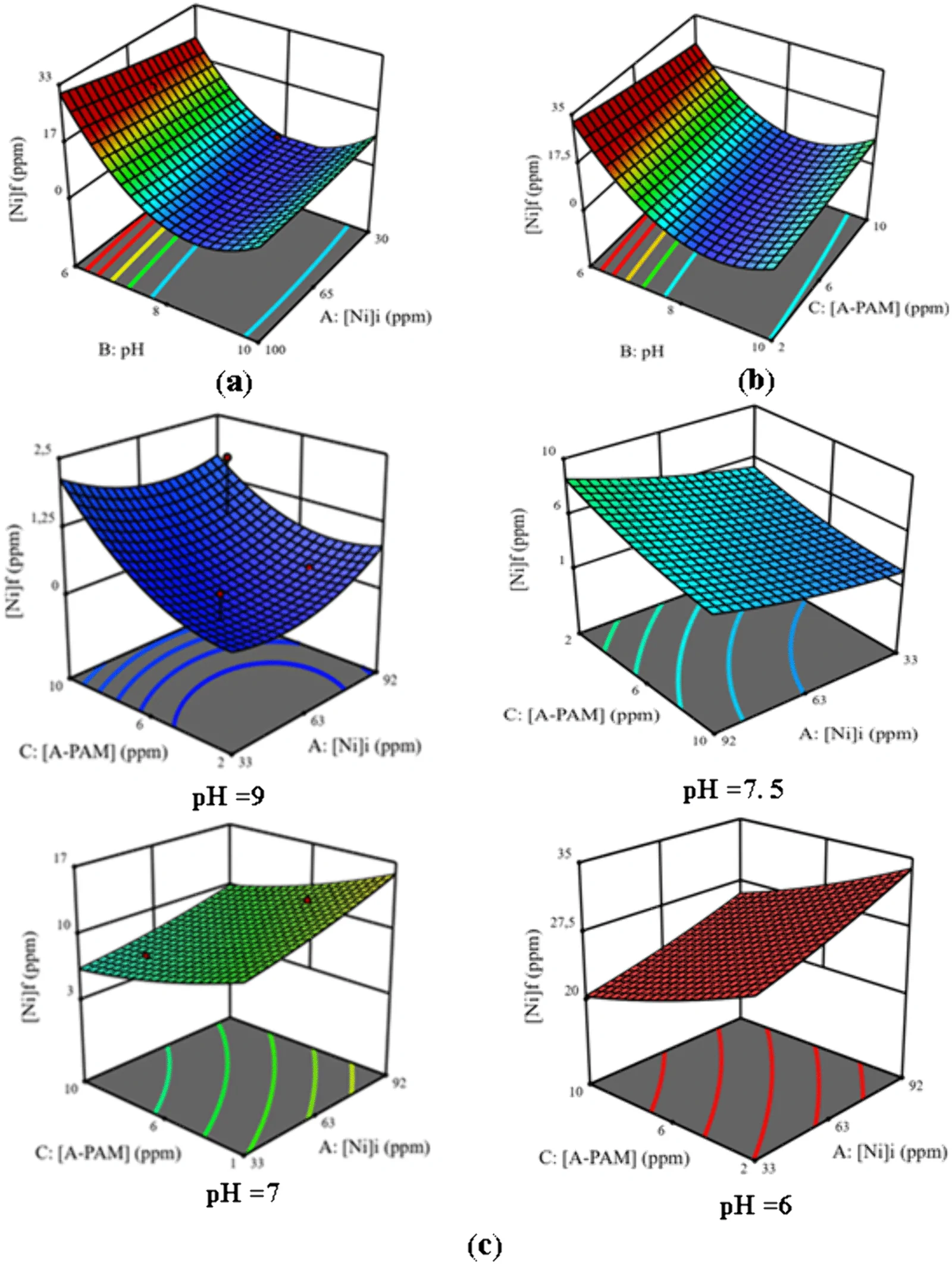
Three-dimensional (3D) response surface plots showing the optimization of nickel removal: (a) the effects of initial nickel concentration ([Ni]_i_) and pH at [A-PAM] = 6 mg/L; (b) the effects of pH and [A-PAM] at [Ni]_i_ = 80 mg/L; and (c) the effects of [Ni]_i_ and [A-PAM] at pH values of 6, 7, 7.5, and 9.

Three-dimensional (3D) response surface plots (Fig 11) illustrate the complex relationships among operational parameters and nickel removal efficiency. These plots reveal interaction effects and delineate optimal operating regions within the experimental domain.

Fig 11a presents the response surface for [Ni]_f_ as a function of [Ni]ᵢ and pH at a constant [A-PAM] of 6 mg/L. The surface shows pronounced second-order polynomial curvature, with a distinct valley corresponding to optimal conditions. pH exerts a dominant influence (*F*-value: 292.64), producing a steep gradient from pH 6–8–9, confirming its controlling role in nickel hydroxide precipitation kinetics [1].

At pH values below 7, insufficient alkalinity leads to elevated residual nickel concentrations across all metal loadings, indicating incomplete precipitation. Optimal conditions occur at pH 8–9, where Ni(OH)_2_ solubility is, and prminimizedecipitation becomes thermodynamically favorable [13,26].

The surface topology reveals a significant pH/[Ni]ᵢ interaction, as indicated by the negative AB coefficient (−1.34). At moderate loadings (10−30 mg/L), performance changes gradually with pH, indicating lower sensitivity to pH optimization. Conversely, at elevated concentrations (60−100 mg/L), steeper pH-dependent gradients indicate that precise pH control is critical for acceptable effluent quality. Contour projections indicate that achieving [Ni]_f_ < 5 mg/L requires pH > 8 for influents at 80−100 mg/L, whereas pH 7–7.5 suffices for [Ni]ᵢ < 50 mg/L, guiding adaptive process control strategies.

Fig 11b shows the response surface for [Ni]_f_ as a function of pH and [A-PAM] at a constant [Ni]ᵢ (80 mg/L). The surface shows pH dominance, with [A-PAM] effects becoming significant only under favorable precipitation conditions. At pH ≤ 7, the surface remains elevated ([Ni]_f_ > 15 mg/L) regardless of flocculant dosage, confirming that insufficient alkalinity prevents effective treatment and that increased polymer addition cannot compensate for thermodynamically unfavorable conditions [26].

As pH increases to 7.5–8, the response surface declines and shows greater curvature along the [A-PAM] axis, underscoring the growing importance of optimizing flocculant use. The optimal [A-PAM] concentration is 6–8 mg/L, at which polymer-enhanced flocculation promotes the aggregation and settling of colloidal hydroxide precipitates. Dosing above 10 mg/L yields diminishing returns and may impair performance by causing charge reversal or destabilizing flocs.

At pH values of 8.5 or higher, the response surface reaches its minimum ([Ni]_f_ < 2–3 mg/L) and exhibits relatively gentle gradients, indicating operational robustness and reduced sensitivity to variations in [A-PAM]. This resilience offers practical benefits by accommodating influent variability without compromising effluent quality. However, the positive B² coefficient (3.98) suggests caution at pH values above 9.5 to prevent potential hydroxide resolubilization under highly alkaline conditions.

Fig 11c shows response surface slices at fixed pH values (6, 7, 7.5, and 9), illustrating how the [Ni]ᵢ/[A-PAM] interaction evolves with pH. At pH 6, the nearly planar surface topology ([Ni]_f_ = 20–35 mg/L) suggests minimal interaction between variables, as both factors exert limited influence under conditions where pH-controlled precipitation is fundamentally constrained.

At pH 7, the response surface begins to curve at [Ni]_f_ = 6–15 mg/L, indicating increased sensitivity to both parameters as hydroxide precipitation becomes thermodynamically favorable.

At pH 7.5, increased curvature indicates a pronounced [Ni]ᵢ/[A-PAM] interaction, and the optimal flocculant dosage shifts slightly higher at elevated metal loadings to accommodate increased solids content and maintain effective flocculation [44].

At pH 9, the system maintains consistently low residual nickel concentrations ([Ni]_f_ < 3 mg/L) across the entire [Ni]ᵢ/[A-PAM] domain, indicating that optimal pH provides the thermodynamic basis for effective treatment. Comprehensive analysis shows that maintaining pH between 8.5 and 9.0, with [A-PAM] dosed at 6–7 mg/L for an influent of 80 mg/L, consistently achieves removal efficiencies greater than 97.5%, thereby ensuring regulatory compliance.

These findings indicate that pH setpoints and flocculant dosing rates should be dynamically adjusted in response to variations in influent concentration to sustain optimal performance in treating post-dechromatization electroplating wastewater (EPW) [2].

As illustrated in Fig 11c, the response surface slices show that increasing pH shifts the system from load-sensitive to load-robust, enabling stable nickel removal across a broad range of influent variability when pH is at least 7.5.

#### 3.2.4 Model validation under industrial conditions.

The predictive reliability of the developed RSM-CCD model was evaluated by validating it against real EPW samples. This approach assessed the model’s accuracy and robustness under industrial conditions, which are characterized by natural fluctuations in influent composition.

The influent nickel concentration was initially determined through laboratory analysis. Using this measured input, the established predictive model was used to calculate the optimal operating conditions, specifically pH and [A-PAM]. Treatment was then applied under these model-recommended conditions, and the resulting effluent nickel concentration was measured to verify the model’s predictions.

The results in Table 7 show strong agreement between predicted and experimentally measured effluent nickel concentrations. For an influent concentration of 62 mg/L, the model predicted a residual nickel concentration of 0.822 mg/L, while the measured value was 0.91 mg/L, yielding a prediction error of 9.67%. This deviation is within acceptable limits for industrial wastewater treatment applications, confirming the model’s predictive accuracy.

**Table 7:** Validation of the optimization model under real-world industrial conditions. (A-PAM: anionic polyacrylamide; [Ni]_f_: final nickel concentration).

Conditions	Response: [Ni]_f_ (mg/L)		Error (%)
	Predicted value	Actual value	
[Ni]_i_ = 62 mg/L pH = 7.96 [A-PAM] = 7.94 mg/L Desirability = 0.158	0.822	0.91	9.67

These findings demonstrate that the developed optimization model performs reliably when applied to industrial wastewater samples. The model’s ability to accurately predict treatment outcomes under real-world operating conditions supports its practical applicability for full-scale implementation and process control.

#### 3.2.5 Experimental verification of the quadratic pH effect and its thermodynamic correlation.

**Fig 12:**
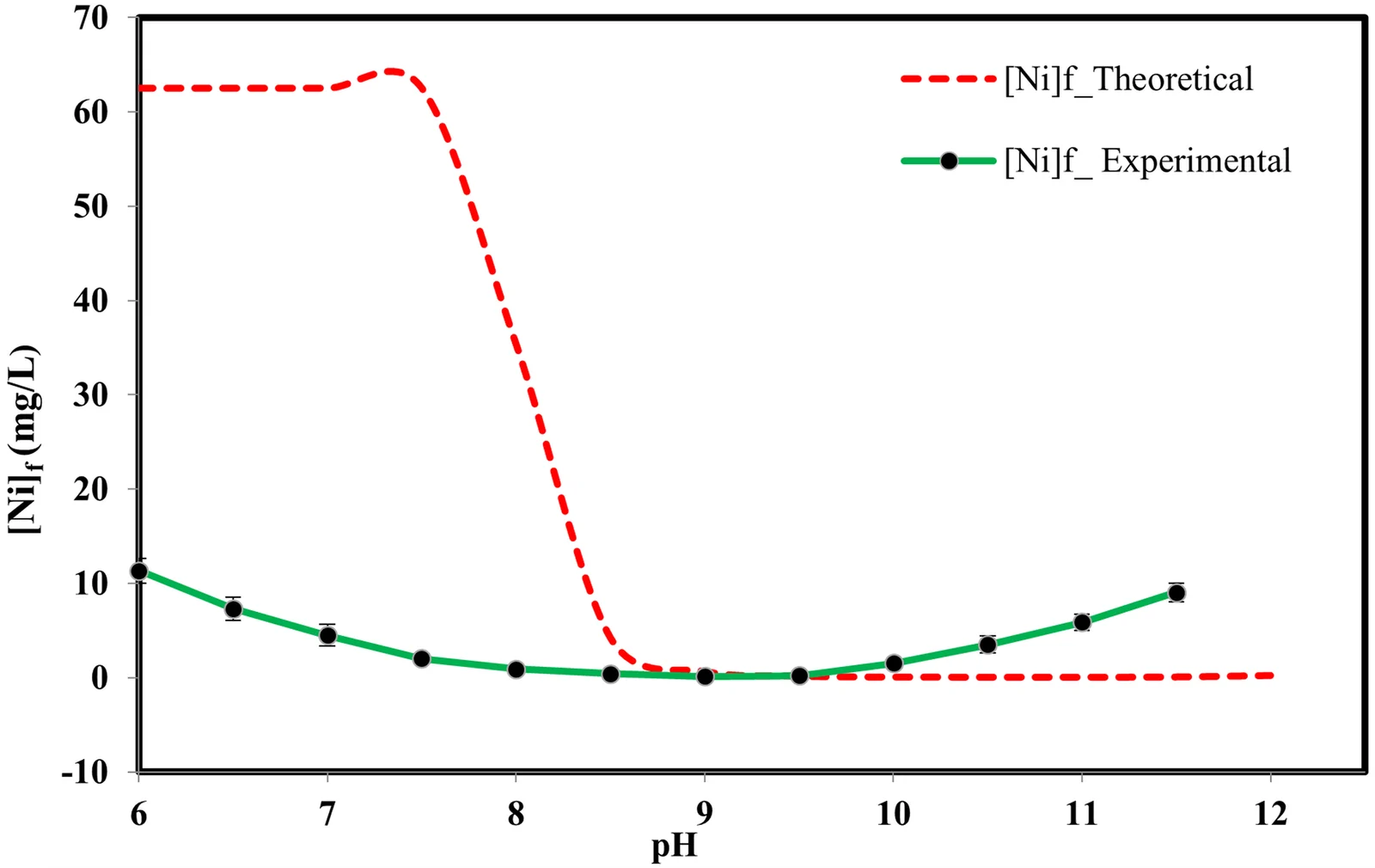
Experimental [Ni]_f_ as a function of pH, compared with the theoretical Ni(OH)_2_ solubility curve (*K*_sp_ = 5.5 × 10^−16^ [49]). Conditions: authentic induE3strial effluent; [Ni]_i_ = 62 mg/L; [A-PAM] = 7.94 mg/L.

To experimentally validate the significant quadratic effect of pH on nickel removal identified by the response surface methodology (RSM) model (B² = +3.98, F = 393.85, *p* < 0.0001), a dedicated laboratory-scale experiment was conducted using authentic industrial E3 effluent. The pH was varied incrementally from 6.0 to 12.0. All other parameters were held constant ([Ni]_i_ = 62 mg/L, [A-PAM] = 7.94 mg/L, temperature = 20–25°C, rapid mixing at 64 rpm for 5 min, followed by slow mixing at 41 rpm for 15 min, and sedimentation for 30 min). The experimental results, illustrated in Fig 12, reveal a characteristic U-shaped solubility profile consistent with the thermodynamic behavior of Ni(OH)_2_. These results are interpreted across three distinct zones as follows.

**Zone 1: pH 6.0–7.5 (Incomplete precipitation):** Residual nickel concentrations remained elevated throughout this range, indicating thermodynamically unfavorable conditions for Ni(OH)_2_ nucleation. At these pH values, the ionic product (*Q* = [Ni²⁺][OH^−^]²) does not exceed the solubility product of Ni(OH)_2_ [49], thereby inhibiting bulk precipitation.

**Zone 2 — pH 8.0–9.5 (Minimum solubility region — optimal zone):** Residual Ni reached its experimental minimum within this range, falling well below the NT 106.002 ONAS discharge limit of 1 mg/L. Notably, the experimentally measured residual concentrations were lower than those predicted by the theoretical solubility curve from the complete Ni(II) speciation model [49] (Equation (4)):


$$\begin{array}{c} \left[\text{Ni}]=[{\text{Ni}}^{\text{2}+}]\times (\text{1}+\beta_{\text{1}}[{\text{OH}}^{-}]+{\beta_{\text{2}}[{\text{OH}}^{-}]}^{\text{2}}+{\beta_{\text{3}}[{\text{OH}}^{-}]}^{\text{3}}+{\beta_{\text{4}}[{\text{OH}}^{-}]}^{\text{4}}\right) \end{array}$$ (4)

In this context, [Ni²⁺] is defined as dividedKsp by [OH^−^]², and *β*₁–*β*_4_ denote the cumulative formation constants of Ni(II) hydroxo-complexes [49]. The observed sub-theoretical performance is attributed to additional removal mechanisms in the bimetallic Cr–Ni system. These include: (i) heterogeneous co-precipitation, in which Ni² ⁺ adsorbs onto the surfaces of freshly formed Cr(OH)_3_ flocs, which are most abundant at pH 7.5–9.0, thereby providing additional removal capacity beyond that predicted by Ni(OH)_2_ solubility equilibrium alone [50]; and (ii) enmeshment of residual colloidal Ni(OH)_2_ microparticles within A-PAM polymer bridging networks, which capture fine suspended precipitate that would otherwise evade sedimentation. The RSM-predicted optimum (pH 8.0–9.0) is approximately 0.5 pH units below the theoretical thermodynamic minimum (pH 9.0–9.5 under ideal conditions of pure water at 25°C). This systematic offset aligns with three concurrent real-matrix effects: (i) metal–organic complexation by EDTA-type ligands and gluconates present in the E3 effluent, which maintain a fraction of Ni²⁺ in the dissolved complexed phase and shift the effective precipitation optimum toward lower pH values, where Ca² ⁺ released by lime competitively displaces Ni²⁺ from its organic complexes [33]; (ii) co-precipitation enhancement by Cr(OH)_3_ surfaces, which is atmaximized pH 7.5–9.0 [50]; and (iii) kinetic limitations inherent to jar-test timescales (30 minutes total contact), during which diffusion-controlled nucleation and crystal growth of Ni(OH)_2_ are incomplete relative to thermodynamic equilibrium predictions.

**Zone 3 — pH 9.5–12.0 (Re-dissolution region):** At pH values above 9.5, residual Ni concentrations increased progressively, directly confirming the re-dissolution phenomenon indicated by the positive quadratic response surface methodology (RSM) coefficient B² = +3.98. This trend is attributed to the sequential formation of soluble anionic hydroxo-complexes under high-alkalinity conditions [49] (Equations (5) and (6)):


$$\begin{array}{c} {\text{Ni}(\text{OH})}_{\text{2}}(\text{s})+{\text{OH}}^{-}\to {\left[{\text{Ni}(\text{OH})}_{\text{3}}\right]}^{-}\;({\text{log}\beta }_{\text{3}}=\text{11}.\text{33}) \end{array}$$ (5)

$$\begin{array}{c} {\text{Ni}(\text{OH})}_{\text{2}}(\text{s})+{\text{2OH}}^{-}\to {\left[{\text{Ni}(\text{OH})}_{\text{4}}\right]}^{\text{2}-}\;({\text{log}\beta }_{\text{4}}=\text{13}.\text{60}) \end{array}$$ (6)

When pH exceeds 10, effluent nickel concentrations exceed the NT 106.002 ONAS discharge limit of 1 mg/L, indicating that excessive alkalization is as operationally detrimental as insufficient alkalization and must be rigorously controlled. The positive quadratic coefficient B² reflects two distinct, experimentally validated physicochemical phenomena: the thermodynamic driving force for Ni(OH)_2_ precipitation, maximized between pH 8.5 and 9.0, and the hydroxo-complex re-dissolution penalty, which becomes significant above pH 9.5. The strong concordance between the RSM-predicted optimum (pH 8.0–9.0) and the experimentally determined minimum solubility region demonstrates that the RSM model effectively captures the thermodynamic and kinetic constraints governing Ni(OH)_2_ precipitation in the complex bimetallic Cr–Ni industrial matrix, despite its empirical foundation.

### 3.3 Innovations and comparative performance of electroplating wastewater (EPW) treatment systems in the context of existing literature

As shown in Table 8, the comparative assessment places the present study within the broader landscape of EPW treatment technologies, highlighting critical trade-offs among performance, economic viability, and operational complexity. A distinguishing feature of this work is its full-scale industrial validation over 24 weeks under real-world process conditions, in contrast to predominantly laboratory-scale investigations [7,10,14], where treatment durations rarely exceed several days under controlled conditions. While laboratory studies consistently report removal efficiencies of 95–99%, the present study’s extended dataset provides empirical evidence that optimized lime-based precipitation maintains high removal efficiencies (99% for Cr, 97% for Ni) despite compositional fluctuations, demonstrating operational resilience that laboratory investigations cannot adequately assess.

**Table 8:** Comparison of electroplating wastewater (EPW) treatment methods reported in the literature and those employed in the current full-scale study.

Ref.	Scale	Treatment	Key Pollutants — Influent→ Effluent (mg/L) (Removal)	Cost (€·m^−3^)	Remarks
**This study**	Full-scale + Lab	PC— Multi-stage 3 configurations: C1: FeCl, C2: PAC and C3: LS	Cr: 70–132 → 0.1–1.03 (99%) Ni: 33–92 → 0.61–4.6 (97%) Cl^−^: 1171–1418 → 588–792 (50%)	C1: 0.432 C2: 0.303 C3: 0.134	RSM–CCD model (*R*² = 0.9917); adaptive dosing; best cost-efficiency
[1]	Full-scale	PC — Multi-stage (PAC/PAMNaCl O, Na_2_S, FeSO_4_)	Cr: 4–332 → 0.53–7.61 (up to 99.8%) Ni: 20–499 → variable (40–90%) COD: 1000–2500 → 400–1000 (24–79%)	Chem: ~ 2.05/ Sludge: ~ 4.97	Inconsistent performance; very high
[26]	Lab + Industrial	PC — Coagulation with *A. squamosa* biomass	Ni: 200–1700 → ~87.6 (90–91%) Turbidity: → 60–64 NTU (>98%)	Low (expected)	Green coagulant; sludge reduced 4 × vs. alum
[10]	Lab	Subcritical hydrothermal + Redox + Adsorption (PVC co-treatment)	Ni² ⁺ : real EPW → ~0.03% residual (99.97%)	—	Exceptional removal; dual-waste valorization; not upscaled
[7]	Lab	Adsorption — UBSB biochar	Cr: 0.44 → ~0 (100%) Ni: 10.1 → variable (variable) Cu: 36.7 → ~0 (90–100%)	Low (expected)	Real EPW tested; long contact time; variable Ni/Zn
[12]	Lab + Pilot	Electrochemical — PE-SACC (sinusoïdal AC, Fe electrodes)	Cu: 50 → 0.12 (99.3%) Zn: 50 → 0.63 (99.1%) Ni: 50 → 0.08 (98.4%)	~0.019 (EEC: 0.105 kWh/m³)	−37% energy vs. DCC; −67% sludge; low COD removal
[14]	Lab	Electrochemical — EO (Ti/TiO_2_-GO-SnO_2_ electrode)	Cr(VI): 100 → 2.45 (97.6%) Zn: 100 → 4.34 (95.7%) COD: 800 → 101 (87.3%)	~1.12 (14.97 kWh/m³)	Novel electrode; high energy cost; continuous mode viable
[25]	Lab	Membrane hybrid — AGMD + SLM + ED	Ni: real EPW → concentrated/recovered AGMD (99.4%)· SLM (76%)· ED (81%)	—	Ni *recovery* focus; complex multi-stage; not yet upscaled

PC: PhysicochemicalConfiguration; C1, C2, C3: 1, 2, 3; FeCl_3_: Ferric chloride; PAC: Polyaluminum chloride; NaClO; Nahypochlorite: Sodium _2_S: Sodium sulfide; FeSO_4_: Ferrous sulfate; Cr: Chromium; Ni: Nickel; Cu: Copper; Zn: Zinc; Cl^−^: Chloride; Chemical ox: CODygen demand; NTU: Nephelometric turbidity unit; EPW: Electroplating wastewater; PVC: Polyvinyl chloride; UBSB: biochar; PE-SACC: Photovoltaic energy-sinusoidal alternating current coagulation; AC: Alternating current; DCC: Direct current control; EEC: Electrical energy consumption; EO: Electrooxidation; Ti/TiO_2_-GO-SnO_2_: Titanium/titanium dioxide-graphene oxide-tin dioxide composite electrode; SS: Stainless steel; AGMD: Air-gap membrane distillation; SLM: Supported liquid membrane; ED: Electrodialysis; RSM: Response surface methodology; CCD: Central composite design; *R*²: Correlation coefficient; Lab: Laboratory scale; LS: Lime slurry.

A comparison with Gao et al. [1], who operated a sophisticated eight-stage system at full scale, is particularly instructive. Despite automated controls and cascaded treatment stages, the facility experienced persistent instability, and treated wastewater frequently failed to meet standards because of improper dosing and unreliable monitoring. These findings indicate that increased system complexity does not ensure treatment stability. The streamlined approach of Configuration 3, which achieves superior performance through optimized chemistry rather than advanced processes, demonstrates that robust treatment fundamentally depends on controlling chemical mechanisms rather than on incorporating multiple unit operations.

The chemical cost of Configuration 3, at 0.134 €·m^−3^, represents an approximately 69% reduction compared to conventional FeCl_3_-based treatment—thereby positioning lime precipitation as one of the most cost-effective options reported. The cost advantage stems from lime’s multifunctional role, which enables pH adjustment, metal precipitation, and coagulation in a single step. This eliminates the need for multiple specialized reagents typically used in conventional systems.

A comparison with the operation described by Gao et al. [1], which reports total costs exceeding 7.80 €·m^−3^, highlights the economic burden associated with process complexity. Elevated costs are attributed to multiple chemical additions across eight stages and significant sludge management expenses (4.97 €·m^−3^). These expenses result from coagulant-polymer precipitation, which generates large volumes of sludge with poor dewatering characteristics. In contrast, lime-based systems produce crystalline metal hydroxide sludges with superior settling properties. This is supported by Bhagavath et al. (2021) [26], who observed a reduction in sludge volume when alum was replaced with lime combinations.

Advanced technologies offer notable benefits but also pose significant challenges. Tang et al.‘s [12] photovoltaic-powered electrocoagulation (EC) achieves high removal efficiency (>98%) at a low operational cost (~0.019 €·m^−3^). However, this method requires substantial capital investment, frequent electrode maintenance (cleaning every 7 days), and the regular addition of conductive salt.

Hybrid membrane processes involve sequential operations, extended treatment times (240 minutes), and complex protocols, making them suitable only for recovering high-value metals [25]. These comparisons indicate that, although advanced technologies demonstrate strong laboratory performance, their operational complexity and economic demands may limit their applicability in standard industrial settings.

A major limitation in existing research is reliance on synthetic effluents, which tend to overestimate industrial treatment performance. In contrast, this study evaluates authentic post-dechromatization wastewater, characterized by variable nickel concentrations (33–92 mg/L), residual chromium, competing metals, organic complexants, and elevated chloride levels, thereby offering a more realistic assessment. Bhagavath et al. [26] reported 90–91% nickel removal from synthetic solutions but observed a significant performance decline with real effluents containing over 1000 mg/L Ni, which required dilution. The identification of metal-ligand complexation, chloride interference, and fixed-dosing limitations reveals challenges common across multiple treatment modalities that are seldom addressed in laboratory studies. These findings emphasize that successful treatment depends on understanding complex wastewater matrices rather than solely on technology selection.

The comparative analysis confirms that the present approach is scientifically rigorous, economically competitive, and industrially viable. Configuration 3 demonstrates high efficiency, 24 weeks of operational stability, and exceptional cost-effectiveness, positioning it favorably against both conventional multi-stage systems and emerging technologies. Systematic optimization of existing chemical treatments, rather than relying on complex new technologies, shows that significant advances remain possible within proven frameworks. Continued progress will require thoughtful process design, full-scale validation, and adaptive optimization using real-time sensing and automated dosage adjustment, building incrementally on principles that have proven effective in industrial practice.

## 4. Environmental implications and research perspectives

Configuration 3 delivers significant environmental improvements that exceed regulatory requirements. Effluent nickel concentrations are consistently reduced from 33–92 mg/L to below 2 mg/L and, with response surface methodology (RSM)-optimized dosing, to below 1 mg/L. This reduction eliminates the acute ecotoxicological risk associated with nickel discharge into receiving water bodies, where bioaccumulation in benthic organisms and fish is a documented concern [51]. Chromium removal rates exceeding 99% mitigate the carcinogenic and mutagenic risks posed by residual Cr(VI) and Cr(III) in aquatic environments [52]. Replacing FeCl_3_ with chloride-free lime reduces secondary chloride loading by approximately 530 mg/L per treatment cycle. As a result, effluent chloride concentrations are maintained at 550–750 mg/L, enabling reuse of treated water for non-potable industrial applications, such as equipment rinsing and cooling circuits, thereby reducing freshwater withdrawal at the facility.

Sludge generated by lime-based precipitation poses both an environmental challenge and an opportunity for resource recovery. Configuration 3 yields crystalline O(NiH)_2_ and Cr(OH)_3_ sludges with improved settling, leading to lower filter press energy use, reduced dewatered cake volume, and lower transportation costs than Configurations 1 and 2. The Ni(OH)_2_-enriched fraction is a promising secondary raw material, recoverable via acidic leaching followed by electrowinning [53]. The Cr(OH)_3_ fraction can be converted to Cr_2_O_3_ via calcination at 800–900°C for use as a pigment, refractory material, or tanning agent [53], thereby facilitating closure of the chromium material cycle. These recovery strategies convert hazardous waste streams into valuable secondary resources, improving the economic viability of the treatment process. Comprehensive sludge characterization, including X-ray Diffraction (XRD) phase analysis, Brunauer-Emmett-Teller (BET) surface area measurement, and quantification of metal content under adaptive dosing conditions, is a key priority for future research aimed at evaluating the technical and economic feasibility of these valorization pathways.

Several avenues should be prioritized in future research. The response surface methodology (RSM) framework, currently focused on nickel as the primary response variable, should be expanded to a multi-response design that simultaneously optimizes the removal of chromium, copper, and zinc, thereby capturing cross-metal interaction effects that this study does not address. Integrating the predictive model into a continuous-flow automated control system that combines online influent monitoring with real-time adaptive dosing constitutes a significant near-term implementation step.

A comprehensive life-cycle cost analysis, incorporating energy consumption, sludge valorization revenues, and capital amortization, is necessary to fully assess the economic benefits of Configuration 3 beyond chemical operating costs. Surface interaction studies, including Langmuir and Freundlich isotherm fitting, SEM-EDX and Fourier transform infrared (FTIR) characterization, and zeta potential measurements as functions of pH and coagulant dose, would clarify the relative contributions of precipitation and surface adsorption to nickel removal, elucidate the electrostatic mechanisms underlying A-PAM bridging efficiency and optimal particle destabilization, and enhance the mechanistic interpretation of the RSM model coefficients. The fate of emerging micro-contaminants (EMCs), including N-nitrosamine precursors from plating-bath surfactants, requires dedicated analytical investigation due to their potential for co-removal with metal hydroxide sludges during precipitation. The variability-responsive RSM methodology developed in this study is also directly applicable to other compositionally variable industrial effluents, such as acid mine drainage, pharmaceutical, and agro-industrial streams. Treating influent fluctuations as explicit design variables rather than uncontrolled disturbances represents a significant methodological advancement for adaptive, regulation-compliant treatment system design.

## 5. Conclusions

Strategic physicochemical reconfiguration, combined with variability-responsive statistical optimization, delivers superior treatment performance, enhanced operational stability, and exceptional economic efficiency in electroplating wastewater treatment. Over 24 weeks of full-scale industrial operation, lime slurry-based Configuration 3 achieved optimal results, removing 99% of chromium and 97% of nickel and reducing chloride levels by approximately 50% compared with conventional FeCl_3_-based treatment. This configuration operated at 0.134 €·m^−3^, representing a 69% reduction relative to Configuration 1 (0.432 €·m^−3^). The principal scientific contribution is a variability-responsive RSM-CCD model ([Ni]_f_ = f([Ni]_i_, pH, [A-PAM], *R*² = 0.9917) that explicitly incorporates fluctuations in influent nickel concentration (33–92 mg/L) as an independent design factor. This approach, implemented for the first time in electroplating wastewater treatment, generates adaptive optimal dosing targets (pH 8.5–9.0, [A-PAM] 6–7 mg/L) that dynamically respond to influent variability, ensuring residual nickel remains below 2 mg/L throughout the operational domain. The quadratic pH effect (B^2^ = +3.98, *p* < 0.0001) was experimentally validated and linked to ththermodynamicallye Ni(OH)_2_ solubility minimum, confirming the physical significance of the model coefficients. These findings demonstrate that substantial performance improvements are achievable within established physicochemical treatment frameworks through intelligent process chemistry and adaptive statistical control, without reliance on capital-intensive emerging technologies.

## Supporting information

S1 Data Underlying data for Figs 1, 2, 3, 6, 7, 8, 9, and 12. (XLSX)

## Abbreviations

A-PAM: Anionic polyacrylamide
AGMD: Air-gap membrane distillation
AF: Antifoaming agent
Al_2_O_3_: Aluminum oxide
ANOVA: Analysis of variance
BET: Brunauer-Emmett-Teller (surface area measurement method)
BOD₅: Biochemical oxygen demand (5 days)
*β*₁–*β*_4_: Cumulative formation constants;
C1, C2, C3: Configuration 1, 2, 3
*C*_chem_: Chemical operating cost (€•m^−3^)
CCD: Central composite design
Cl^−^: Chloride ion
COD: Chemical oxygen demand
Cr(III): Trivalent chromium
Cr(VI): Hexavalent chromium
Cu (Cu²⁺): Copper
DCC: Direct current coagulation
DF: Desirability function
*D*_i_: Dosage of reagent *i* (kg•m^−3^)
DXA: Twips Per Pixel (docx unit specification)
EC: Electrocoagulation
EC_w_: Electrical conductivity of water
ED: Electrodialysis
EDTA: Ethylenediaminetetraacetic acid
EEC: Electrical energy consumption
E1: Chromic acidic effluents
E2: Alkaline effluents
E3: Initial reference wastewater
EMCs: Emerging contaminants
EO: Electrooxidation
EPW: Electroplating wastewater
°C: Degrees Celsius
€•m^−3^: Euros per cubic meter
F-value: Fisher value
Fe: Iron
FeCl_3_: Ferric chloride
FeSO_4_: Ferrous sulfate
f-EPW: Final treated electroplating wastewater
FTIR: Fourier transform infrared Spectroscopy
H_2_SO_4_: Sulfuric acid
h: Hours
HMs: Heavy metals
ICP: Inductively coupled plasma
INNORPI: Tunisian Institute of Standardization
[Ni]ᵢ: Initial nickel concentration
[Ni]_f_: Final nickel concentration
*K*_sp_: Solubility product constant
LMH: Liters per square meter per hour
LS: Lime slurry
LSTM: Long Short-Term Memory (neural network)
mg/L: Milligrams per liter
min: Minutes
MS: Mean square
NaCl: Sodium chloride
NaClO: Sodium hypochlorite
Na_2_S: Sodium sulfide
NaHSO_3_: Sodium bisulfite
NH_4_⁺-N: Ammonium nitrogen
Ni(II): Divalent nickel
NO_3_^−^-N: Nitrate nitrogen
NT 106.002: Tunisian Standard 106.002
NTU: Nephelometric turbidity unit
OC: Operating cost
ONAS: National Office of Sanitation
OPs: Organic pollutants
PAC: Polyaluminum chloride
PAM: Polyacrylamide
Pb: Lead
PC: Physicochemical
PE-SACC: Photovoltaic energy–sinusoidal alternating current coagulation
PHD: Public hydraulic domain
P_i_: Unit price of reagent *i* (€/kg)
PLC: Programmable logic controller
*p*-value: Probability value
PV: Photovoltaic
PVC: Polyvinyl chloride
Q: Ionic product
*R*²: Correlation coefficient
*R*²adj: Adjusted correlation coefficient
*R*²pred: Predicted correlation coefficient
rpm: Revolutions per minute
RSM: Response Surface Methodology
SACC: Sinusoidal alternating current coagulation
SEM-EDX: Scanning Electron Microscopy - Energy Dispersive X-ray
SLM: Supported liquid membrane
SS: Stainless steel
TN: Total nitrogen
TOC: Total organic carbon
TP: Total phosphorus
TSS: Total suspended solids
UBSB: Biochar
XRD: X-ray Diffraction
Zn (Zn²⁺): Zinc
