# Supplementary material for: Integrated optimization of electroplating wastewater treatment: A comparative assessment of physicochemical treatment configurations and process enhancement using response surface methodology
Source: PLoS One. 2026 Aug 21;21(8):e0345965. doi: 10.1371/journal.pone.0345965 (PMC13496402; doi:10.1371/journal.pone.0345965)
Supplement: Supplementary file 1 [file pone.0345965.s002.pdf]

PLOS One

Integrated Optimization of Electroplating Wastewater Treatment: Comparative Performance Assessment of Physicochemical Configurations and Response Surface Methodology-Based Process Enhancement

--Manuscript Draft--

**Manuscript Number:**	PONE-D-26-12528
**Article Type:**	Research Article
**Full Title:**	Integrated Optimization of Electroplating Wastewater Treatment: Comparative Performance Assessment of Physicochemical Configurations and Response Surface Methodology-Based Process Enhancement
**Short Title:**	RSM-Based Optimization of Physicochemical Electroplating Wastewater Treatment
**Corresponding Author:**	Soumaya Ibrahimi<br>University of Gabes National Engineering School of Gabes: Universite de Gabes Ecole Nationale d'Ingenieurs de Gabes<br>Gabes, TUNISIA
**Keywords:**	Electroplating wastewater (EPW); Lime Slurry; Response Surface Methodology; optimization; operating costs
**Abstract:**	This investigation evaluates physicochemical treatment methods for industrial electroplating wastewater (EPW). Monitoring at a Tunisian full-scale facility revealed significant instability in conventional systems, with residual nickel fluctuating from 0.02 to 60 mg/L and chloride persistently exceeding regulatory limits (442-1418 mg/L). Three configurations were compared over 24 weeks: ferric chloride-based coagulation (Configuration 1), polyaluminum chloride coagulation (Configuration 2), and lime slurry-based chemical attack (Configuration 3). Configuration 3 demonstrated superior consistency, achieving 99% chromium removal, 97% nickel removal, and 50% chloride reduction, while reducing chemical operating costs by 69% (0.134 vs. 0.432 €/m3). This study introduces a novel optimization strategy that explicitly incorporates influent nickel concentration variability (33–92 mg/L) as an independent factor within a Response Surface Methodology–Central Composite Design, alongside pH (6.3–9.7) and flocculant dosage (2.6–9.4 mg/L). By treating wastewater composition fluctuations as a controlled design variable rather than experimental noise, the approach generated a robust predictive model ([Ni]f = f([Ni], pH, [A-PAM])) with exceptional accuracy (R2 = 0.9917). The derived equation enables real-time adaptive chemical dosing, allowing operators to dynamically calculate optimal pH and flocculant requirements based on measured influent concentration. Statistical analysis revealed critical pH-metal loading interactions (F = 5.70, p = 0.044), confirming that optimal parameters shift systematically with influent composition. Under model-predicted conditions, residual nickel consistently remained below 2 mg/L despite extreme influent variability, ensuring regulatory compliance. This integrated approach demonstrates that variability-responsive optimization simultaneously enhances treatment efficacy, operational stability, and economic efficiency.
**Order of Authors:**	Soumaya Ibrahimi
	Aicha Gasmi
	Karim Kriaa
	Farid Fadhillah
	Majeed Ali Habeeb
	Djamel Ghernaout
	Noureddine Elboughdiri
	Ahmed Hannachi
**Additional Information:**	
**Question**	**Response**

Powered by Editorial Manager® and ProduXion Manager® from Aries Systems Corporation

**Financial Disclosure**<br><br>Enter a financial disclosure statement that describes the sources of funding for the work included in this submission. Review the submission guidelines for detailed requirements. View published research articles from *PLOS ONE* for specific examples.<br><br>This statement is required for submission and **will appear in the published article** if the submission is accepted. Please make sure it is accurate.<br><br>**Funded studies**<br>Enter a statement with the following details:<br>• Initials of the authors who received each award<br>• Grant numbers awarded to each author<br>• The full name of each funder<br>• URL of each funder website<br>• Did the sponsors or funders play any role in the study design, data collection and analysis, decision to publish, or preparation of the manuscript?<br><br>Did you receive funding for this work?	Yes
Please add funding details.<br>as follow-up to "**Financial Disclosure**<br><br>Enter a financial disclosure statement that describes the sources of funding for the work included in this submission. Review the submission guidelines for detailed requirements. View published research articles from *PLOS ONE* for specific examples.<br><br>This statement is required for submission and **will appear in the published article** if the submission is accepted. Please make sure it is accurate.	This research was supported and funded by the Deanship of Scientific Research at Imam Mohammad Ibn Saud Islamic University (IMSIU) (grant number IMSIU-DDRSP2602).

**Funded studies**<br>Enter a statement with the following details:<br>• Initials of the authors who received each award<br>• Grant numbers awarded to each author<br>• The full name of each funder<br>• URL of each funder website<br>• Did the sponsors or funders play any role in the study design, data collection and analysis, decision to publish, or preparation of the manuscript?<br><br>Did you receive funding for this work?"	
Please select the country of your main research funder (please select carefully as in some cases this is used in fee calculation).<br>as follow-up to "**Financial Disclosure**<br><br>Enter a financial disclosure statement that describes the sources of funding for the work included in this submission. Review the submission guidelines for detailed requirements. View published research articles from *PLOS ONE* for specific examples.<br><br>This statement is required for submission and **will appear in the published article** if the submission is accepted. Please make sure it is accurate.<br><br>**Funded studies**<br>Enter a statement with the following details:<br>• Initials of the authors who received each award<br>• Grant numbers awarded to each author<br>• The full name of each funder<br>• URL of each funder website<br>• Did the sponsors or funders play any role in the study design, data collection and analysis, decision to publish, or preparation of the manuscript?<br><br>Did you receive funding for this work?"	TUNISIA - TN
**Competing Interests**	We certify that we have no financial or personal relationship with any person or

Use the instructions below to enter a competing interest statement for this submission. On behalf of all authors, disclose any competing interests that could be perceived to bias this work—acknowledging all financial support and any other relevant financial or non-financial competing interests.<br><br>This statement is required for submission and **will appear in the published article** if the submission is accepted. Please make sure it is accurate and that any funding sources listed in your Funding Information later in the submission form are also declared in your Financial Disclosure statement.<br><br>View published research articles from *PLOS ONE* for specific examples.<br><br>**NO authors have competing interests**<br><br>Enter: *The authors have declared that no competing interests exist.*<br><br>**Authors with competing interests**<br><br>Enter competing interest details beginning with this statement:<br><br>*I have read the journal's policy and the authors of this manuscript have the following competing interests: [insert competing interests here]*<br><br>* typeset	organization that could improperly influence our work. In addition, we have no professional or personal interest in any product, service, or company that could be perceived as influencing the views expressed in the manuscript or its evaluation.
**Ethics Statement**<br><br>Enter an ethics statement for this submission. This statement is required if the study involved:<br><br>• Human participants<br>• Human specimens or tissue<br>• Vertebrate animals or cephalopods<br>• Vertebrate embryos or tissues	Not applicable

• Field research<br><br>Write "N/A" if the submission does not require an ethics statement.<br><br>General guidance is provided below. Consult the submission guidelines for detailed instructions. **Make sure that all information entered here is included in the Methods section of the manuscript.**	

Powered by Editorial Manager® and ProduXion Manager® from Aries Systems Corporation

**Format for specific study types**<br><br>**Human Subject Research (involving human participants and/or tissue)**<br>• Give the name of the institutional review board or ethics committee that approved the study<br>• Include the approval number and/or a statement indicating approval of this research<br>• Indicate the form of consent obtained (written/oral) or the reason that consent was not obtained (e.g. the data were analyzed anonymously)<br><br>**Animal Research (involving vertebrate animals, embryos or tissues)**<br>• Provide the name of the Institutional Animal Care and Use Committee (IACUC) or other relevant ethics board that reviewed the study protocol, and indicate whether they approved this research or granted a formal waiver of ethical approval<br>• Include an approval number if one was obtained<br>• If the study involved *non-human primates*, add *additional details* about animal welfare and steps taken to ameliorate suffering<br>• If anesthesia, euthanasia, or any kind of animal sacrifice is part of the study, include briefly which substances and/or methods were applied<br><br>**Field Research**<br><br>Include the following details if this study involves the collection of plant, animal, or other materials from a natural setting:<br>• Field permit number<br>• Name of the institution or relevant body that granted permission	
**Data Availability**<br><br>Authors are required to make all data underlying the findings described fully available, without restriction, and from the time of publication. PLOS allows rare exceptions to address legal and ethical concerns. See the PLOS Data Policy and FAQ for detailed information.	Yes - all data are fully available without restriction

A Data Availability Statement describing where the data can be found is required at submission. Your answers to this question constitute the Data Availability Statement and **will be published in the article**, if accepted.<br><br>**Important:** Stating 'data available on request from the author' is not sufficient. If your data are only available upon request, select 'No' for the first question and explain your exceptional situation in the text box.<br><br>Do the authors confirm that all data underlying the findings described in their manuscript are fully available without restriction?	
**Describe where the data may be found in full sentences. If you are copying our sample text, replace any instances of XXX with the appropriate details.**<br><br>• If the data are **held or will be held in a public repository**, include URLs, accession numbers or DOIs. If this information will only be available after acceptance, indicate this by ticking the box below. For example: *All XXX files are available from the XXX database (accession number(s) XXX, XXX.)*.<br>• If the data are all contained **within the manuscript and/or Supporting Information files**, enter the following: *All relevant data are within the manuscript and its Supporting Information files.*<br>• If neither of these applies but you are able to provide **details of access elsewhere**, with or without limitations, please do so. For example:<br><br>*Data cannot be shared publicly because of [XXX]. Data are available from the XXX Institutional Data Access / Ethics Committee (contact via XXX) for researchers who meet the criteria for access to confidential data.*<br><br>*The data underlying the results presented in the study are available from (include the name of the third party*	All relevant data are within the manuscript and its Supporting Information files.

and contact information or URL).<br>• This text is appropriate if the data are owned by a third party and authors do not have permission to share the data.<br><br>* typeset	
Additional data availability information:	

Integrated Optimization of Electroplating Wastewater Treatment: Comparative Performance Assessment of Physicochemical Configurations and Response Surface Methodology-Based Process Enhancement

Soumaya Ibrahimi[1*], Aicha Gasmi[1], Karim Kriaa[2], Farid Fadhillah[2*], Majeed Ali Habeeb[3], Djamel Ghernaout[4], Noureddine Elboughdiri[4], Ahmed Hannachi[1]

[1] Department of Chemical Engineering, Laboratory of Engineering Processes and Industrial Systems, National School of Engineering of Gabes, University of Gabes, 6029 Gabes, Tunisia

[2] College of Engineering, Imam Mohammad Ibn Saud Islamic University (IMSIU), Riyadh 11432, Saudi Arabia.

[3] University of Babylon, College of Education for Pure Sciences, Department of Physics, Iraq, Babil

[4] Department of Chemical Engineering, College of Engineering, University of Ha'il, PO Box 2440, Ha'il 81441, Saudi Arabia

[*]Corresponding Authors: soumaya.ibrahimi@univgb.tn; fffadhillah@imamu.edu.sa

Abstract

This investigation evaluates physicochemical treatment methods for industrial electroplating wastewater (EPW). Monitoring at a Tunisian full-scale facility revealed significant instability in conventional systems, with residual nickel fluctuating from 0.02 to 60 mg/L and chloride persistently exceeding regulatory limits (442-1418 mg/L). Three configurations were compared over 24 weeks: ferric chloride-based coagulation (Configuration 1), polyaluminum chloride coagulation (Configuration 2), and lime slurry-based chemical attack (Configuration 3). Configuration 3 demonstrated superior consistency, achieving 99% chromium removal, 97% nickel removal, and 50% chloride reduction, while reducing chemical operating costs by 69% (0.134 vs. 0.432 €/m³).

This study introduces a novel optimization strategy that explicitly incorporates influent nickel concentration variability (33–92 mg/L) as an independent factor within a Response Surface Methodology–Central Composite Design, alongside pH (6.3–9.7) and flocculant dosage (2.6–9.4 mg/L). By treating wastewater composition fluctuations as a controlled design variable rather than experimental noise, the approach generated a robust predictive model ($[Ni]_f = f([Ni]_i, pH, [A\text{-}PAM])$) with exceptional accuracy ($R^2 = 0.9917$). The derived equation

enables real-time adaptive chemical dosing, allowing operators to dynamically calculate optimal pH and flocculant requirements based on measured influent concentration. Statistical analysis revealed critical pH-metal loading interactions ($F = 5.70$, $p = 0.044$), confirming that optimal parameters shift systematically with influent composition. Under model-predicted conditions, residual nickel consistently remained below 2 mg/L despite extreme influent variability, ensuring regulatory compliance. This integrated approach demonstrates that variability-responsive optimization simultaneously enhances treatment efficacy, operational stability, and economic efficiency.

Keywords Electroplating wastewater (EPW), Lime Slurry, Response Surface Methodology, optimization, operating costs.

Highlights

- Lime slurry treatment achieved 99% Cr and 97% Ni removal from electroplating wastewater.
- RSM-CCD optimization yielded a robust predictive model ($R^2 = 0.9917$) for adaptive dosing.
- pH–metal loading interactions were identified as key drivers of treatment performance.
- A 69% cost reduction confirms lime slurry as an economically viable industrial solution.
- Systematic optimization of proven methods can deliver transformative performance gains.

Abbreviations

AGMD	Air-gap membrane distillation	E3	Initial reference wastewater
AF	Antifoaming agent	Fe	Iron
Al_2O_3	Aluminum oxide	$FeCl_3$	Ferric chloride
ANOVA	Analysis of variance	$FeSO_4$	Ferrous sulfate
A-PAM	Anionic polyacrylamide	f-EPW	Final treated electroplating
BOD_5	Biochemical oxygen demand (5	F-value	Fisher value
C1, C2, C3	Configuration 1, 2, 3	H_2SO_4	Sulfuric acid
CCD	Central composite design	HMs	Heavy metals
Cl^-	Chloride ion	ICP	Inductively coupled plasma
COD	Chemical oxygen demand	LMH	Liters per square meter per hour
Cr(III)	Trivalent chromium	LS	Lime slurry
Cr(VI)	Hexavalent chromium	MS	Mean square
Cu (Cu^{2+})	Copper	NaCl	Sodium chloride
DCC	Direct current coagulation	NaClO	Sodium hypochlorite
DF	Desirability function	Na_2S	Sodium sulfide
EC	Electrocoagulation	NH_4^+-N	Ammonium nitrogen
ECw	Electrical conductivity of water	Ni(II)	Divalent nickel
ED	Electrodialysis	$[Ni]_i$	Initial nickel concentration
EEC	Electrical energy consumption	[Ni]f	Final nickel concentration
EMCs	Emerging contaminants	NO_3^--N	Nitrate nitrogen
EO	Electrooxidation	NT	Tunisian Standard 106.002
EPW	Electroplating wastewater	NTU	Nephelometric turbidity unit
E1	Chromic acidic effluents	OC	Operating cost
E2	Alkaline effluents	ONAS	National Office of Sanitation

OPs	Organic pollutants	RSM	Response Surface Methodology
PAC	Polyaluminum chloride	SACC	Sinusoidal alternating current
PAM	Polyacrylamide	SBR	Sequencing batch reactor
Pb	Lead	SLM	Supported liquid membrane
PC	Physicochemical	SS	Stainless steel
PE-SACC	Photovoltaic energy–sinusoidal	TN	Total nitrogen
PHD	Public hydraulic domain	TOC	Total organic carbon
PLC	Programmable logic controller	TP	Total phosphorus
PV	Photovoltaic	TSSs	Total suspended solids
PVC	Polyvinyl chloride	UBSB	Biochar
p-value	Probability value	Zn (Zn^{2+})	Zinc
R^2	Correlation coefficient	ZLD	Zero-liquid discharge
R^2adj	Adjusted correlation coefficient		
R^2pred	Predicted correlation coefficient		

1. Introduction

Electroplating plays a key role in modern manufacturing by providing functional surface coatings that enhance corrosion resistance, mechanical performance, and aesthetic quality across industries such as automotive, electronics, aerospace, and consumer goods **[1]**. However, these processes generate complex industrial effluents containing high concentrations of heavy metals (HMs), notably chromium (Cr), nickel (Ni), copper (Cu), zinc (Zn), and lead (Pb), elevated salinity, variable pH, and significant organic pollutants (OPs) from additives, surfactants, and cleaning agents **[2,3]**. In addition, electroplating wastewater (EPW) may contain emerging contaminants (EMCs), such as N-nitrosamines **[4]**, which are of growing concern due to their carcinogenic properties. When inadequately treated, EPW poses serious environmental and public health risks because of the toxicity, persistence, and bioaccumulative behavior of both HMs and EMCs in aquatic systems **[5,6]**.

Among HM contaminants, hexavalent chromium (Cr(VI)) and divalent nickel (Ni(II)) are of particular concern because of their widespread use in electroplating operations and well-established toxicological effects **[7]**. Cr(VI) is recognized for its carcinogenicity and acute ecotoxicity **[2]**, while nickel exhibits carcinogenic, allergenic, and chronic ecological effects **[8]**. Additional challenges arise from the widespread use of chloride-based coagulants, such as ferric chloride ($FeCl_3$), which, although efficient for metal removal, increase chloride levels in treated effluents and may limit water reuse because of discharge limits **[2,9]**. Moreover, strong metal–organic complexes formed by plating additives can inhibit conventional precipitation mechanisms, allowing residual dissolved metals to persist despite appropriate chemical dosing and pH control **[2,10]**.

As a result, discharge regulations have become increasingly stringent worldwide, requiring treatment systems that can ensure stable compliance under variable operating conditions

[11,12]. In this regulatory context, Tunisia has adopted the NT 106.002 standard as a binding reference for controlling industrial effluent quality, particularly for metal-finishing processes such as electroplating **[11]**.

Several strategies are currently used to treat EPW, primarily chemical precipitation **[13]**, coagulation-flocculation **[9]**, electrochemical treatment **[14,15]**, adsorption **[16,17]**, electrodialysis **[18]**, and membrane filtration **[19]**. Advanced sequential treatment approaches have also been developed to address the complexity of EPW pollutants **[20-23]**. While these technologies can achieve satisfactory pollutant removal individually, their standalone implementation is often insufficient to meet stringent discharge regulations or water reuse requirements **[24,25]**. Consequently, chemical treatment—particularly precipitation—remains a cornerstone of EPW management due to its high effectiveness at elevated pollutant concentrations, simplicity, and relatively low cost **[2]**. However, its performance is highly sensitive to operating conditions and often unstable in real industrial settings, mainly due to fluctuations in wastewater composition associated with production cycles, bath renewal, and rinsing practices **[1]**.

Despite extensive research, knowledge gaps remain regarding the long-term performance, operational robustness, and economic sustainability of physicochemical (PC) treatment systems under real-world industrial conditions. Most studies are limited to laboratory-scale experiments or short-term trials with synthetic or compositionally stable wastewaters, which do not capture the temporal and compositional variability of industrial effluents **[1]**. Moreover, process optimization has traditionally relied on univariate approaches or statistical designs that assume constant influent characteristics—an assumption that poorly reflects industrial reality and limits system adaptability **[26]**.

In this context, Response Surface Methodology (RSM) is a powerful yet underutilized tool for systematically optimizing EPW treatment processes **[27-28]**. However, most RSM-based studies fail to explicitly incorporate influent metal concentration variability as a design parameter, treating it as experimental noise or artificially stabilizing it. Addressing this limitation is essential for developing adaptive, robust treatment strategies that maintain high performance amid real-time fluctuations.

Accordingly, the present study proposes an integrated experimental and analytical framework to evaluate, optimize, and conduct an economic assessment of alternative PC treatment configurations for industrial EPW. The main objectives are to: (i) assess the long-term performance of a full-scale conventional treatment system under real-world operating conditions; (ii) compare three PC configurations based on $FeCl_3$, polyaluminum chloride

(PAC), and lime treatments in terms of efficiency, stability, and robustness; (iii) conduct a comparative economic analysis focused on chemical consumption costs; and (iv) implement an innovative RSM-based optimization strategy that explicitly integrates influent nickel concentration variability as an independent design variable, alongside pH and flocculant dosage.

By explicitly incorporating influent variability into the optimization framework, this work moves beyond conventional static treatment design toward a variability-aware, predictive control strategy. The proposed approach enables the development of a quantitative dosing model that links influent metal concentration to optimal operating conditions, providing a practical tool for real-time process adjustment. The novelty of this study lies in transforming influent variability from a limiting constraint into a central optimization parameter, offering a robust pathway toward more resilient, cost-effective, and environmentally sustainable EPW treatment systems that maintain consistent performance under inherently variable industrial conditions.

2. Materials and Methodology

2.1. Conventional Physicochemical (PC) treatment process and electroplating wastewater (EPW) segregation

In this study, the EPW samples analyzed were collected from the industrial facility under investigation in Tunisia. They are segregated at the source by chemical type to prevent incompatible reactions and improve overall treatment efficiency. In particular, chromic acidic effluents (E1) and alkaline effluents (E2) are managed separately due to their distinct chemical characteristics and treatment requirements. Chromic effluents mainly originate from surface treatment and rinsing operations, whereas alkaline effluents are produced during degreasing and alkaline cleaning stages.

The industrial treatment line follows a conventional PC scheme widely used in electroplating facilities. E1 effluents are first subjected to a chromium reduction step that converts Cr(VI) to Cr(III).

The reduced chromic effluents are then combined with alkaline streams to promote neutralization and metal hydroxide precipitation, followed by chemical precipitation, solid–liquid separation, sludge dewatering, and final pH adjustment prior to discharge. The resulting effluent from mixing the alkaline and dechromated streams constitutes the initial reference wastewater (E3) used throughout the remainder of this study.

2.2. Performance monitoring of the final treated electroplating wastewater (f-EPW) after actual process

This assessment aims to characterize the temporal variability of treated EPW under real-world process conditions and to examine how fluctuations in influent quality affect overall treatment performance.

To comprehensively characterize wastewater quality and assess the operating conditions of the reference treatment process, samples were collected at key points along the treatment line, including the treatment stage (E3) and the final clarified effluent (f-EPW).

Sampling campaigns were conducted weekly over one full operational year under the precipitation-based treatment process, capturing temporal variability related to production schedules, operational fluctuations, and seasonal effects. Immediately after collection, samples were preserved at 4°C and transported to the laboratory for analysis. All PC analyses were performed within 48 hours to minimize sample degradation and ensure data reliability.

The monitored parameters included pH, electrical conductivity (ECw), total suspended solids (TSSs), chemical oxygen demand (COD), biochemical oxygen demand (BOD_5), chloride concentration, and HM content (i.e., Cr^{3+} and Ni^{2+}).

As summarized in Table 1, the comparative analysis between E3 and f-EPW reveals both the effectiveness and critical limitations of the current treatment system. The existing process demonstrates notable success in pH neutralization, achieving complete pH remediation. Organic matter reduction shows variable performance, with COD removal efficiencies ranging from 49-90% and BOD_5 reductions of 20-86%. While minimum f-EPW values meet regulatory compliance, substantial fluctuations and maximum concentrations exceed NT 106.002 standards. TSS removal exhibits extreme variability (2-118 mg/L in f-EPW vs. 2 ± 0.3 mg/L in E3), suggesting operational inconsistencies or insufficient settling efficiency. However, the most critical deficiency concerns HM and chloride removal, which were found to be limiting factors.

The temporal evolution of trivalent chromium (Cr(III)) concentrations (Fig. 1) indicates that Cr(III) levels remained relatively low during most of the operational period; however, sporadic peaks reaching up to 6.03 mg/L were recorded, occasionally exceeding the discharge limit of 2 mg/L. These episodic exceedances suggest that the conventional process is sensitive to sudden changes in influent composition and operational disturbances.

As illustrated in Fig. 2, Nickel removal performance exhibited even greater instability. Residual Nickel concentrations in the treated effluent fluctuated widely, from 0.02 to 60 mg/L. Several

high-concentration episodes were observed, indicating that the conventional process cannot consistently maintain regulatory compliance under elevated influent Nickel loading. This pronounced variability confirms that Nickel removal efficiency is strongly governed by fluctuations in influent characteristics.

Chloride concentration emerged as the most critical constraint in the conventional treatment process. As shown in Fig. 3, chloride levels in the treated effluent ranged from 442 to 1418 mg/L over the monitoring period, frequently exceeding the Tunisian discharge limit of 700 mg/L. This persistent chloride enrichment is primarily attributed to the extensive use of $FeCl_3$ as the main coagulant **[9]**. Upon dissolution, $FeCl_3$ releases chloride ions (Cl-) and hydrolyzes to form ferric hydroxide precipitates, which are essential for coagulation. However, excessive dosing increases chloride loading and generates acidity, consuming alkalinity and lowering pH. These effects induce operational instability and require additional chemical inputs for pH adjustment, thereby increasing chemical consumption and operating costs (OCs). Moreover, elevated chloride concentrations limit the reuse potential of the treated effluent and compromise regulatory compliance.

Overall, the long-term monitoring results show that although the conventional treatment process can perform acceptably under favorable operating conditions, its efficiency is strongly constrained by the pronounced temporal variability in EPW composition. These findings highlight the limitations of the current treatment strategy and clearly justify the need to develop and evaluate alternative or optimized treatment processes that reduce chloride accumulation while ensuring stable and efficient HM removal. This rationale underpins the investigation of the second treatment process, presented and discussed in the following section.

Table 1. Temporal fluctuations of key physicochemical (PC) parameters in electroplating wastewater (EPW) and comparison with regulatory discharge limits. (COD: Chemical Oxygen Demand; EC_W: Electrical conductivity of water; f-EPW: final treated electroplating wastewater; TSS_S: Total Suspended Solids; E3: Initial Reference Wastewater).

Parameter	E3	f-EPW	Discharge limits NT 106.002	
			PHD [1]	ONAS [2]
pH	2.1± 0.5	6.5-9	6.5-8.5	6.5-9
TSS_S (mg/L)	2±0.3	2-118	30	400
EC_W (µS/cm)	7923-8261	2720-6100	-	-
COD (mg/L)	299- 464	30-235	90	1000
DBO_5 (mg /L)	74-226	10-180	30	400
Cl^- (mg/L)	1171-1815	442-1418	600	700

[1] NT 106.002 for discharge into Public Hydraulic Domain (PHD).

[2] NT 106.002 for discharge into National Office of Sanitation (ONAS) public sewerage network.

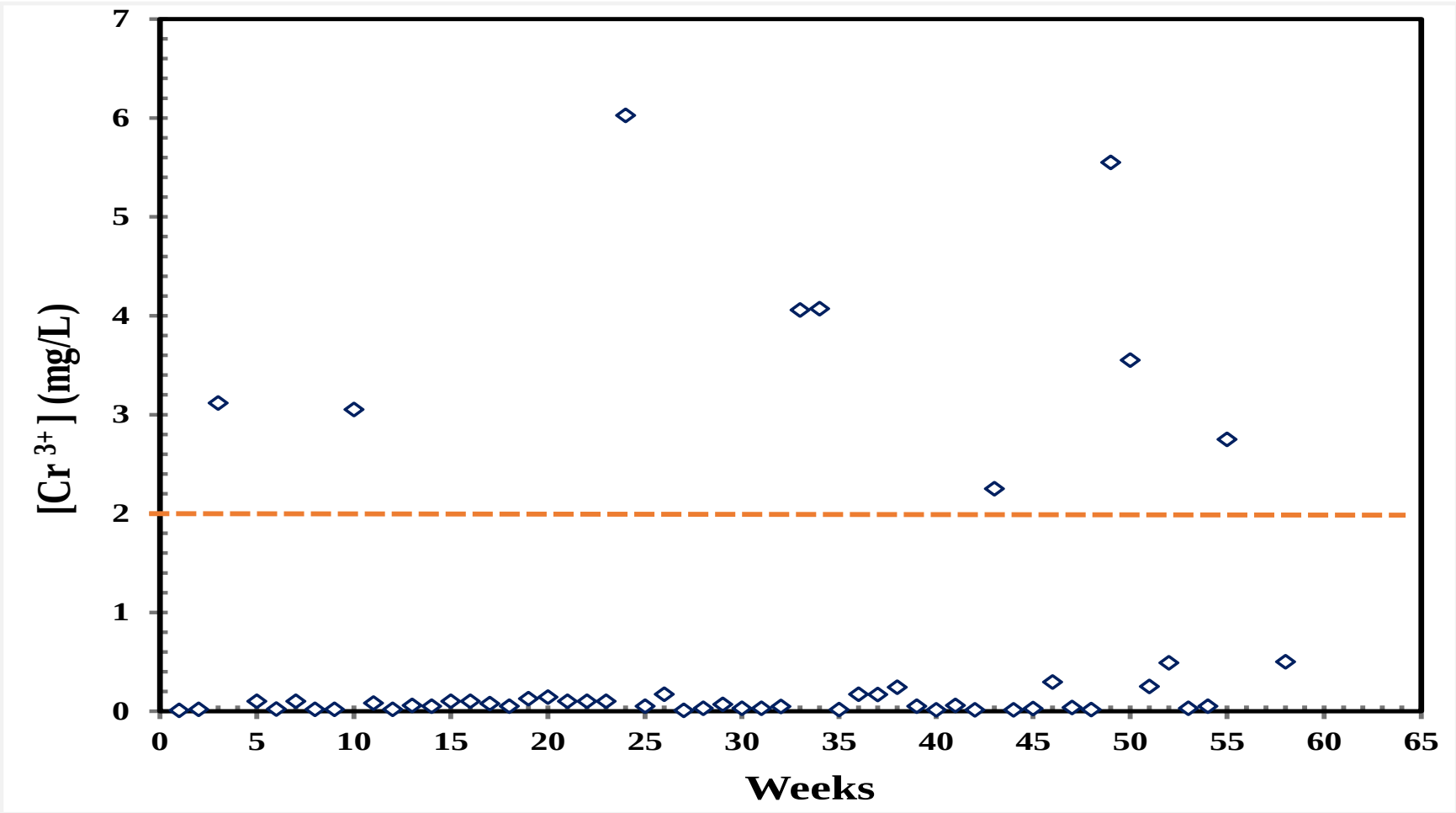

Fig. 1. Temporal fluctuations in Cr^{3+} concentration in the f-EPW.

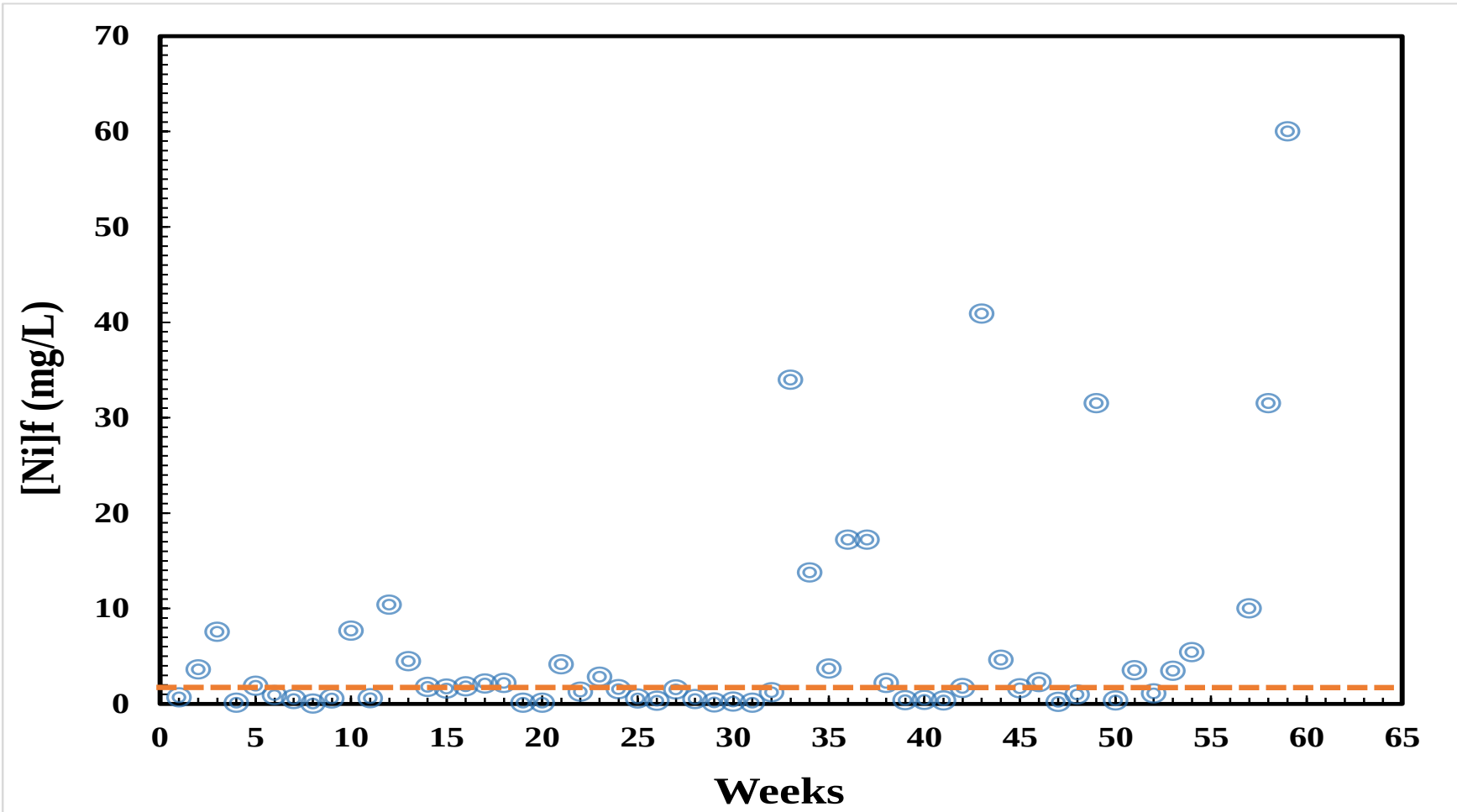

Fig. 2. Temporal fluctuations in Ni concentration in the f-EPW.

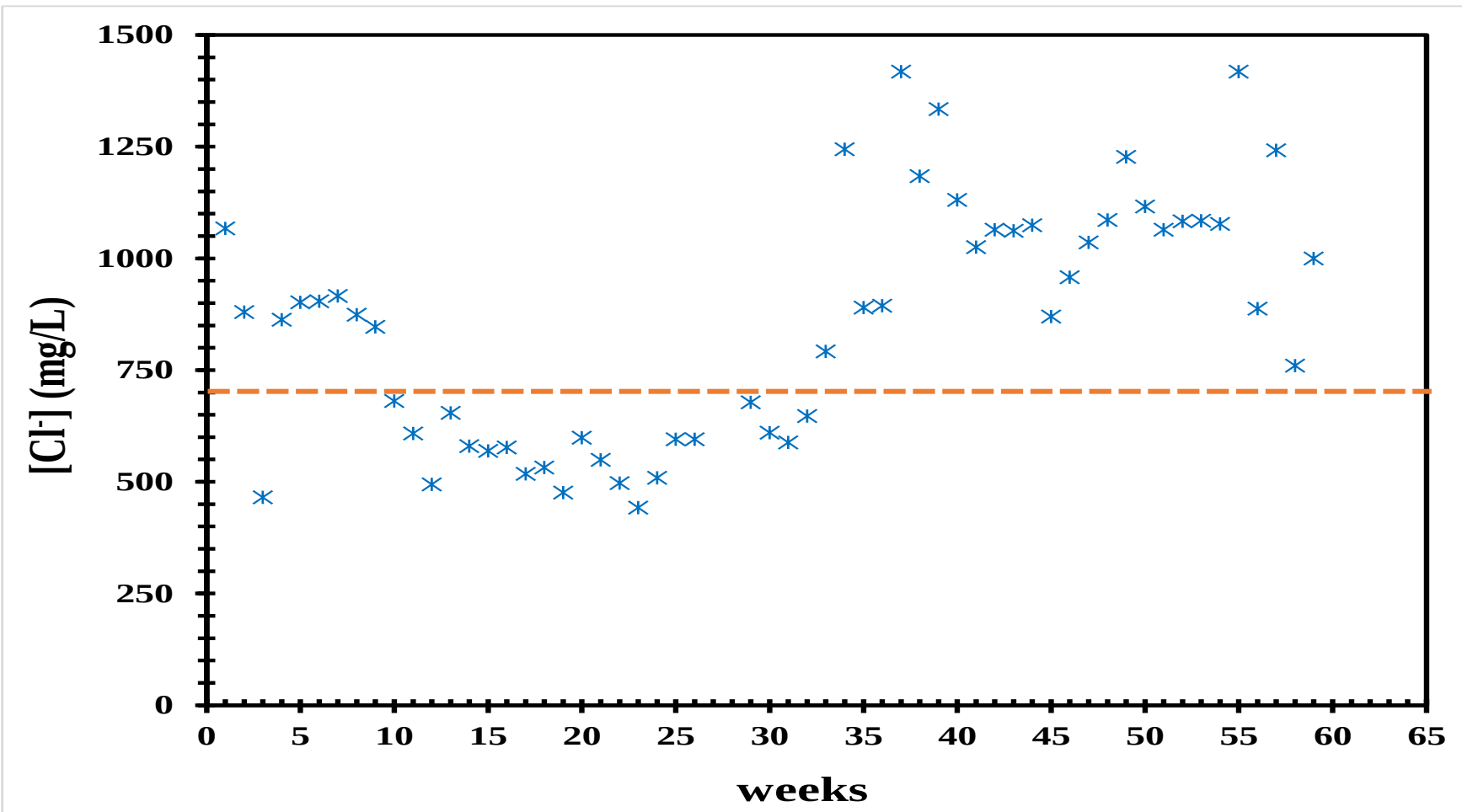

Fig.3. Temporal fluctuations in Cl^- concentration in the f-EPW.

2.3. Methodological Framework and Treatment Configurations

As illustrated in Fig. 4, the experimental framework in this study was designed to progressively improve EPW treatment performance by comparing three successive treatment configurations.

All configurations follow the same global PC treatment scheme described in Section 2.1 and Fig. 4, and the same reference influent (E3), corresponding to the mixed alkaline and dechromated effluent, was systematically used as the initial wastewater stream.

The three treatment processes differ only in the chemical precipitation step, while all upstream and downstream unit operations (neutralization, solid–liquid separation, sludge dewatering, and final pH adjustment) remain identical. This approach enabled a direct and consistent comparison of the effects of precipitation chemistry on pollutant removal efficiency, treatment stability, and effluent quality.

- **Configuration 1: Reference conventional precipitation configuration**

Configuration 1 corresponds to the reference configuration. It uses a conventional PC precipitation scheme with $FeCl_3$ as the primary coagulant, followed by pH adjustment before flocculation.

This configuration served as the baseline for evaluating all subsequent process modifications. Although widely implemented in industrial electroplating facilities, preliminary monitoring revealed limitations in residual chloride accumulation and incomplete removal of complexed metal species.

- **Configuration 2: Modified precipitation process with optimized coagulation**

To address the limitations of the reference configuration while preserving the overall treatment structure, a modified process (Configuration 2) was proposed. In this configuration, $FeCl_3$ was replaced with PAC as the coagulant, and pH adjustment was optimized before the precipitation step.

The primary objective of this modification was to reduce chloride input associated with chemical addition while enhancing the efficiency of pollutant destabilization and aggregation. This was achieved by maintaining the same global treatment layout as Configuration 1.

- **Configuration 3: Chemical attack by lime slurry (LS)**

A second alternative configuration (Configuration 3) was implemented, replacing the conventional coagulation-based precipitation step with a lime slurry (LS)–based chemical attack. Following implementation, Configuration 3 was selected for further optimization using RSM coupled with a Central Composite Design (CCD) to identify optimal operating conditions and maximize treatment performance.

Overall, this comparative methodological framework provides a systematic basis for evaluating precipitation strategies, thereby facilitating the selection and optimization of the most efficient PC treatment configuration.

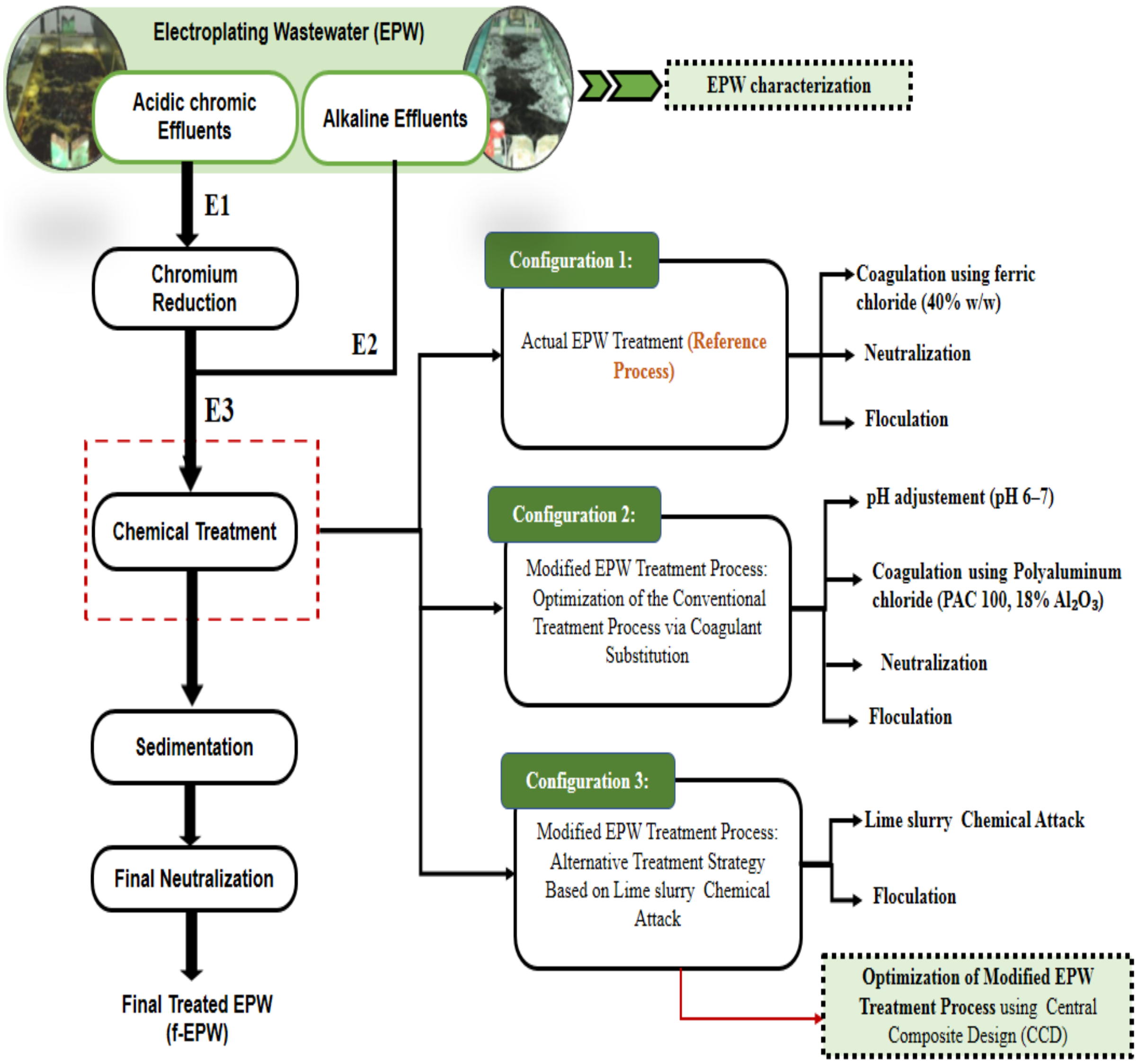

Fig. 4. Conceptual framework of the experimental methodology and the treatment configurations evaluated in this study.

2.4. Analytical methods

The PC parameters used to evaluate the performance of the EPW investigated in this study were systematically analyzed using standardized and validated analytical methods.

pH was measured with a calibrated digital pH meter, and EC_w (µS/cm) was measured with a conductivity meter, providing an indicator of the wastewater's ionic strength and dissolved salt content.

TSSs were quantified using the standard gravimetric method in accordance with APHA guidelines (APHA, 2017) **[29]**. This method involved filtering samples through pre-weighed membrane filters, drying at 105°C, and reweighing to determine suspended particulate concentrations.

COD was measured by the dichromate reflux method (ISO 15705) **[30]**, which quantifies the total chemically oxidizable organic matter in wastewater. BOD_5 was determined by the standard dilution and incubation procedure (APHA, 2017) **[29]**, providing an estimate of the biodegradable organic load.

Chloride ions (Cl^-, mg/L) were analyzed by argentometric titration using Mohr's method (APHA et al., 2017) **[29]**. HMs, including chromium (Cr(III)), nickel (Ni), Cu, Zn, iron (Fe), and Pb, were quantified by inductively coupled plasma spectroscopy (ICP-AES, Activa-HORIBA JOBIN YVON). This analytical technique enabled accurate determination of trace metal concentrations, which are critical indicators for assessing EPW contamination and treatment efficiency.

2.5. Operating Cost (OC) Analysis

Following the comparative methodological framework, an OC analysis was conducted to evaluate the economic performance of the three PC treatment configurations (Processes 1, 2, and 3). To ensure consistency and comparability, the assessment was limited to the chemical operating cost (C_{chem}, €/m³), which reflects the consumption of chemical reagents directly associated with each configuration.

All three configurations were operated under identical hydraulic and operational conditions, including the same treatment capacity, mixing intensity, and hydraulic residence time. Consequently, the electrical energy demand for pumping and agitation was assumed to be equivalent across configurations and was therefore excluded from the comparative analysis. Under these controlled conditions, the primary source of economic differentiation among the processes is the type and dosage of chemical reagents employed.

Focusing on C_{chem} provides a rigorous and meaningful basis for comparing the relative economic performance of the three treatment configurations without introducing bias related to infrastructure or energy consumption **[1]**.

The specific chemical OC was calculated using equation (1):

$$\mathbf{C_{chem}} = \sum_{i} \left(\boldsymbol{D_i x P_i} \right)_{\boldsymbol{i}} \quad \textbf{(1)}$$

where: i denotes the chemical reagents considered in each configuration: $FeCl_3$, sulfuric acid (H_2SO_4), antifoaming agent (AF), and flocculant for Configuration 1; PAC 100, H_2SO_4, AF, and flocculant for Configuration 2; and LS, AF, and flocculant for Configuration 3. D_i (kg·m⁻³) denotes the dosage of reagent i, and P_i (€/kg) denotes its unit price.

2.6. Experimental apparatus and operating parameters for treatment process optimization (Configuration 3)

Optimization of Configuration 3 was performed using a standard jar-test apparatus with six programmable paddle mixers and parallel 1-L beakers (Fig. 5). Three key operational parameters were systematically investigated: initial nickel concentration ($[Ni]_i$) in the E3 effluent, initial pH (adjusted with LS), and flocculant dosage (anionic polyacrylamide, [A-PAM]). LS was selected as the neutralizing agent for its proven effectiveness in HM precipitation and cost-efficiency in industrial-scale EPW treatment **[31,32]**.

For each experimental run, 300 mL of E3 effluent was dispensed into six parallel jars, and predetermined doses of LS and flocculant were added according to the experimental design matrix. The treatment sequence consisted of two main stages: (i) neutralization at 64 rpm for 5 minutes to ensure pH adjustment, complete reagent dispersion, and floc formation, followed by slow flocculation at 41 rpm for 15 minutes to promote floc growth; and (ii) quiescent settling for 30 minutes to allow gravitational separation of the formed flocs.

These operational parameters (mixing speeds and durations) were kept consistent with the current industrial treatment process to enable direct comparison and practical implementation. After sedimentation, supernatant samples were carefully collected from each jar for analysis of residual pollutant concentrations, including nickel, chromium, COD, and other target parameters. All experiments were conducted at ambient temperature (20-25°C) and performed in triplicate to ensure statistical reliability.

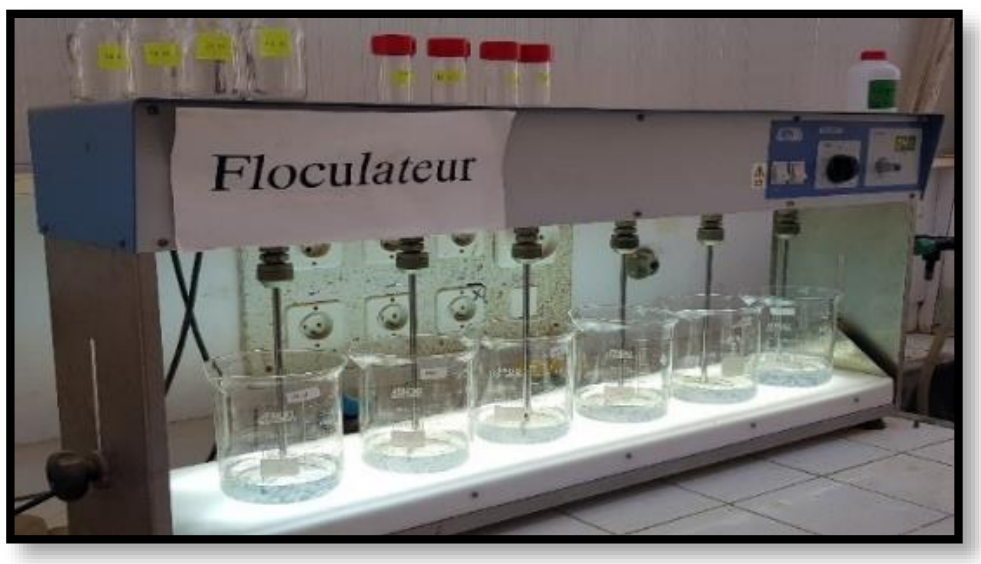

Fig. 5. Experimental jar-test apparatus and instrumentation.

2.7. Response Surface Methodology (RSM) Model

2.7.1. Preliminary trials: pH Adjustment and Metal Hydroxide Precipitation Behavior

Preliminary experiments were conducted to investigate the pH-adjustment behavior and metal hydroxide precipitation characteristics of the EPW ($[Ni]_i$ = 92 mg/L). The presence of chromium significantly influences the precipitation behavior of nickel in the bimetallic system.

Although literature reports individual precipitation optima at pH 6.4 for Cr(III) and pH 8.5 for Ni(II) **[31]**, the titration curve (Fig. 6) shows that complete neutralization and alkaline conditions (pH 9-10) are achieved at LS dosages above 5 mL/L (400 mg/L, based on an LS concentration of 80 g/L). This pH range ensures simultaneous precipitation of both metal hydroxides, as confirmed by the pH stabilization plateau. The steep transition zone between 3-5 mL/L (240-400 mg/L), around pH 6-9, corresponds to the equivalence point region, where buffering capacity is minimal and pH control is critical. Therefore, maintaining pH ≥ 9 with adequate lime dosage is essential to maximize metal removal efficiency in the bimetallic Cr-Ni system.

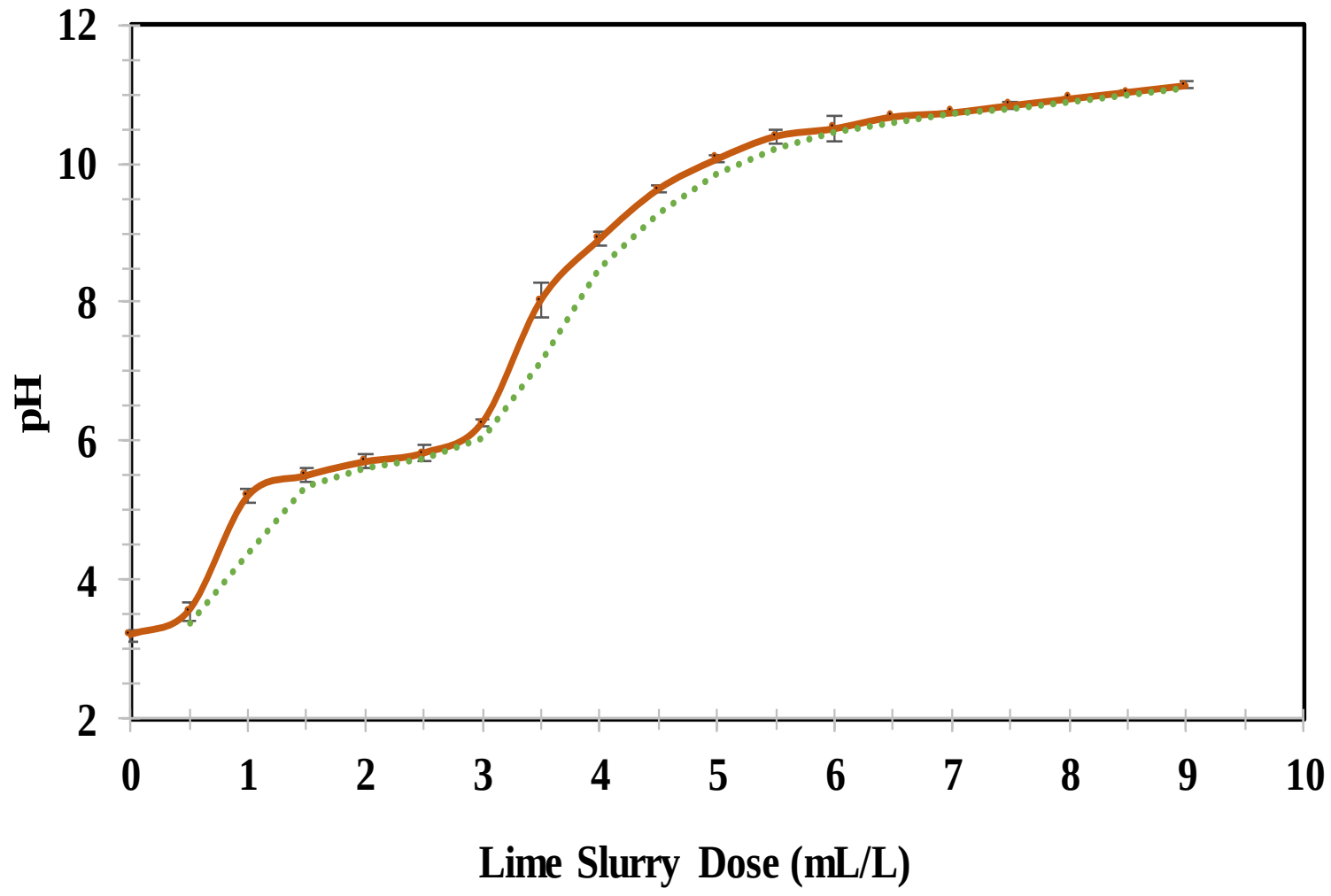

Fig. 6. Evolution of pH as a function of Lime Slurry (LS) dosage during preliminary neutralization tests of electroplating wastewater (EPW).

2.7.2. Experimental Design

A CCD was implemented within the RSM framework to systematically evaluate how key operational parameters affect Nickel removal efficiency from EPW. CCD is a well-established second-order experimental design that estimates quadratic and interaction effects while minimizing the number of experimental runs, making it particularly suitable for process optimization studies. In the present work, the experimental design, data analysis, and model development were carried out using Design-Expert® software.

Among the three wastewater treatment processes previously investigated, the most effective method was selected for further optimization based on the results of comparative experiments. Following the selection of the optimal treatment process, three independent variables were identified for optimization based on the outcomes of preliminary experiments and supported by relevant literature. These variables included $[Ni]_i$, solution pH, and flocculant dosage ([A-PAM]). These parameters were recognized as the most influential factors governing nickel

hydroxide precipitation, floc formation, and settling behavior, and, consequently, overall purification performance **[32,33]**.

An A-PAM was used as the flocculant because of its strong affinity for $Ni(OH)_2$ precipitates and its proven effectiveness in EPW treatment **[34]**. The flocculant dosage was varied according to the CCD matrix to evaluate its effects on Nickel removal efficiency and floc settling performance.

The experimental ranges for the selected variables were determined from preliminary results and electroplating plant datasets. $[Ni]_i$, defined as the nickel concentration measured immediately after the dechromatation step, was varied from 33 to 92 ppm. This range was selected based on the annual variability of post-dechromatation nickel concentrations (E3) observed in the industrial monitoring database in the year preceding the study.

Therefore, the chosen interval reflects realistic operating conditions and ensures the practical relevance of the experimental design. The pH range and [A-PAM] were determined from preliminary tests and literature data on polyacrylamide application in EPW treatment **[33,34]**. These ranges were selected to encompass both suboptimal and near-optimal operating conditions and to enable identification of significant linear, quadratic, and interaction effects. The primary response variable was the residual Nickel concentration in the treated effluent. Table 2 presents the experimental domain and the corresponding coded levels of the independent variables used in the CCD.

Table 2. Experimental ranges and levels of independent variables used in the Response Surface Methodology (RSM). (A-PAM: Anionic Polyacrylamide; $[Ni]_i$: Nickel concentration before treatment.)

**Variable**	**-α**	**-1**	**0**	**+1**	**+α**
A: $[Ni]_i$ (ppm)	33	45	62.5	80	92
B: pH	6.32	7	8	9	9.68
C: [A-PAM] (ppm)	2.6	4	6	8	9.36

3. Results and discussion

3.1. Comparative Evaluation of The Treatment Configurations under Successive Operating Periods and Process Stability Assessment

3.1.1. Final Treated Electroplating Wastewater (f-EPW) Quality and Pollutant Removal Efficiency

The long-term stability of industrial wastewater treatment processes is a critical performance criterion, as treatment systems must operate reliably despite temporal fluctuations in influent composition **[1]**. **Figs**. 7–9 present the 24-week monitoring results for chloride, chromium, and nickel concentrations in the treated effluents of the three configurations, with post-dechromatation wastewater (E3) as the reference influent.

Chloride removal remained limited in Configuration 1, with effluent concentrations ranging from 800 to 1500 mg/L and high variability throughout the monitoring period. This modest performance is primarily attributed to the use of $FeCl_3$ as a coagulant, which introduces additional Cl- and inherently limits net chloride removal **[35,36]**.

Building on this baseline, Configuration 2 showed notable improvement, maintaining effluent concentrations between 800 and 1100 mg/L with greater stability. Replacing $FeCl_3$ with PAC avoided secondary chloride inputs and enabled partial removal by incorporating chloride into precipitated solids **[37]**. However, the most significant advancement was achieved with Configuration 3, which showed progressively decreasing effluent concentrations that stabilized between 550 and 750 mg/L during the final monitoring phase, particularly during weeks 17–24. This represents approximately a 50% reduction compared to the influent baseline, an improvement linked to lime-based neutralization **[38]**.

While chloride removal showed a clear progression in efficiency across configurations, chromium removal exhibited marked differences primarily in stability rather than average efficiency. Configurations 1 and 2 showed considerable variability, with concentration peaks reaching 2–2.5 mg/L during weeks 1–2 for Configuration 1 and fluctuating values occasionally exceeding 2 mg/L for Configuration 2, particularly during weeks 9–11. In sharp contrast, Configuration 3 consistently maintained very low chromium concentrations, predominantly below 1 mg/L during weeks 17–24, with only occasional data points near 1 mg/L. The sequential acidification–neutralization strategy provides superior control of chromium speciation and precipitation kinetics **[38]**.

Among the three target pollutants, nickel removal posed the greatest challenge due to the high residual concentration after dechromatization. Configurations 1 and 2 showed pronounced variability, similar to their chromium removal patterns, with effluent concentrations ranging from approximately 2 mg/L to peaks of 12 mg/L in Configuration 2 at week 11 and 17 mg/L in

Configuration 1 during week 1. This instability highlights the limited buffering capacity of the conventional coagulation system.

Following the trend observed for chloride and chromium, process configuration 3 also showed improved performance in nickel removal, with effluent concentrations generally maintained between 1 and 5 mg/L, though occasional points reached approximately 6.5 mg/L, notably in week 17. During the stable operating period spanning weeks 19–24, concentrations frequently approached or fell below 1 mg/L, confirming the advantage of lime-based precipitation with optimized pH control **[38]**. Nevertheless, residual fluctuations indicate that even this configuration remains sensitive to influent variability, though to a significantly lesser extent than conventional approaches.

The consistent superiority of Configuration 3 across all monitored parameters can be attributed to several synergistic factors. Lower standard deviations for chromium and nickel confirm its superior operational resilience, which stems from the buffering capacity of excess lime, faster hydroxide precipitation kinetics, and the formation of more stable metal hydroxide phases rather than metal–coagulant complexes in conventional systems **[39]**. These advantages translate into both higher average removal efficiencies and substantially reduced temporal variability.

Despite these clear advantages, all three configurations share a fundamental limitation: reliance on fixed chemical dosing strategies. Influential nickel variability—driven by changes in electroplating operations, rinsing practices, and dechromatization efficiency—cannot be adequately managed under static operating conditions **[1]**. Consequently, periods of under- or over-dosing remain unavoidable, affecting both treatment consistency and chemical consumption efficiency. This limitation becomes particularly evident during the early weeks of operation for each configuration, when adaptation to influent characteristics appears to require gradual optimization.

In summary, Configuration 3 represents the most effective current treatment strategy, achieving approximately 50% chloride removal, exceeding 99% chromium removal with exceptional stability during the final monitoring period, and approximately 95–97% nickel removal, all with enhanced stability under realistic industrial conditions. However, achieving consistently robust performance under highly variable influent conditions will require coupling this configuration with advanced control strategies, such as real-time metal monitoring and adaptive dosing systems **[40]**. Such process intensification would enable dynamic adjustment of lime dosage and pH set-points, thereby transforming process configuration 3 from a superior static process into a fully adaptive and industrially resilient treatment solution.

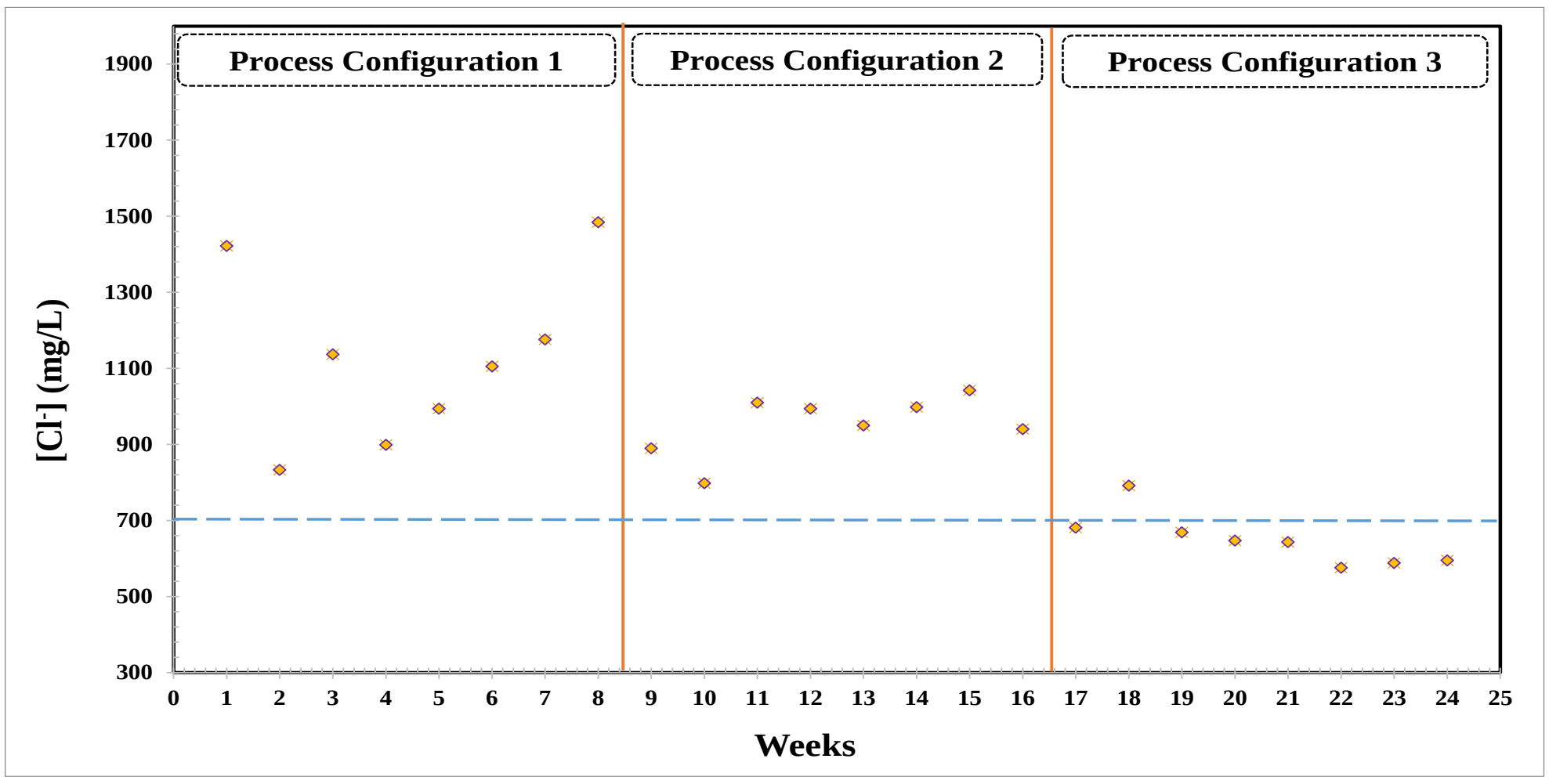

Fig. 7. Chloride concentration in f-EPW: process stability assessment over 24 weeks.

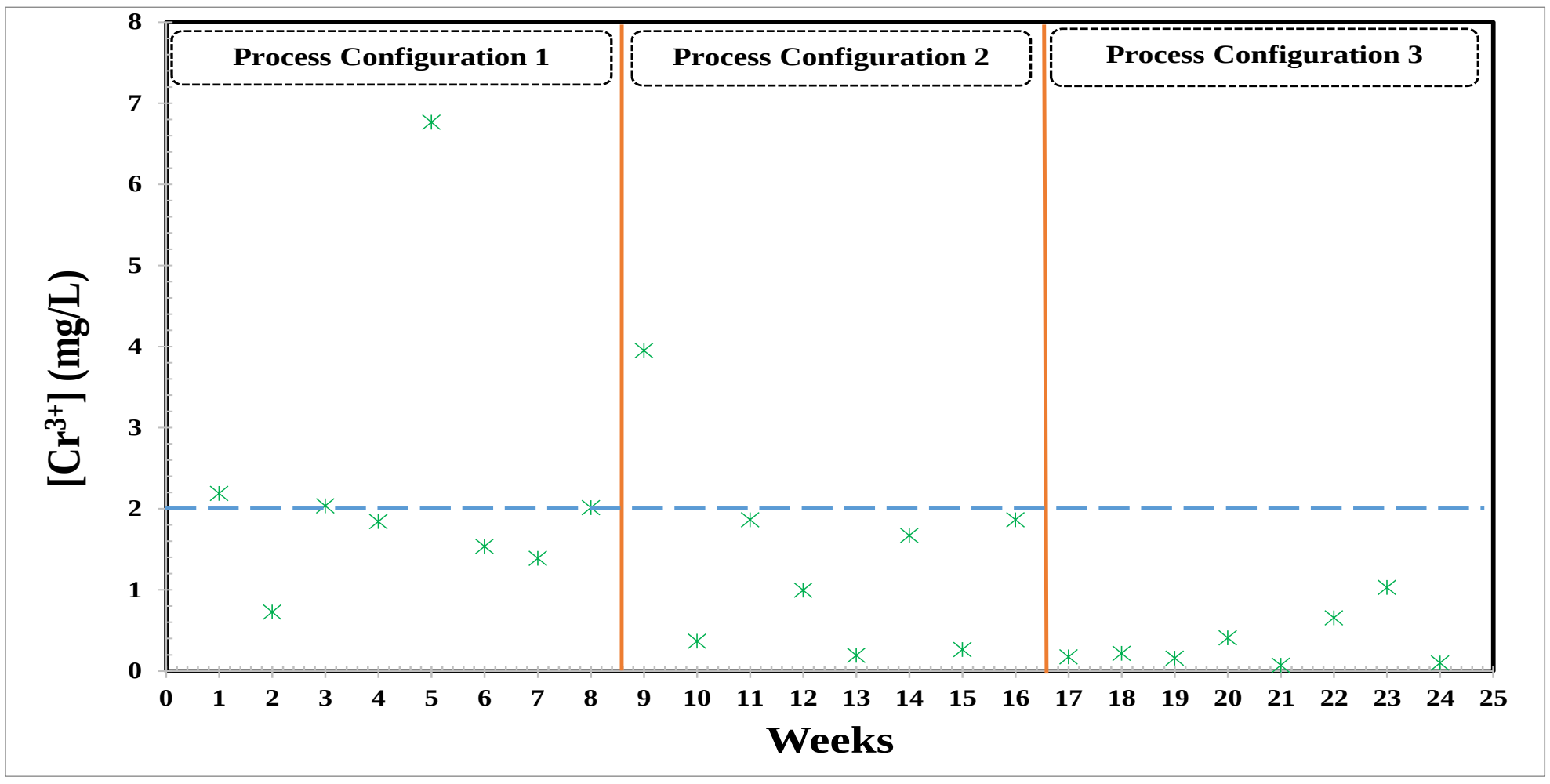

Fig. 8. Chromium concentration in f-EPW: process stability assessment over 24 weeks.

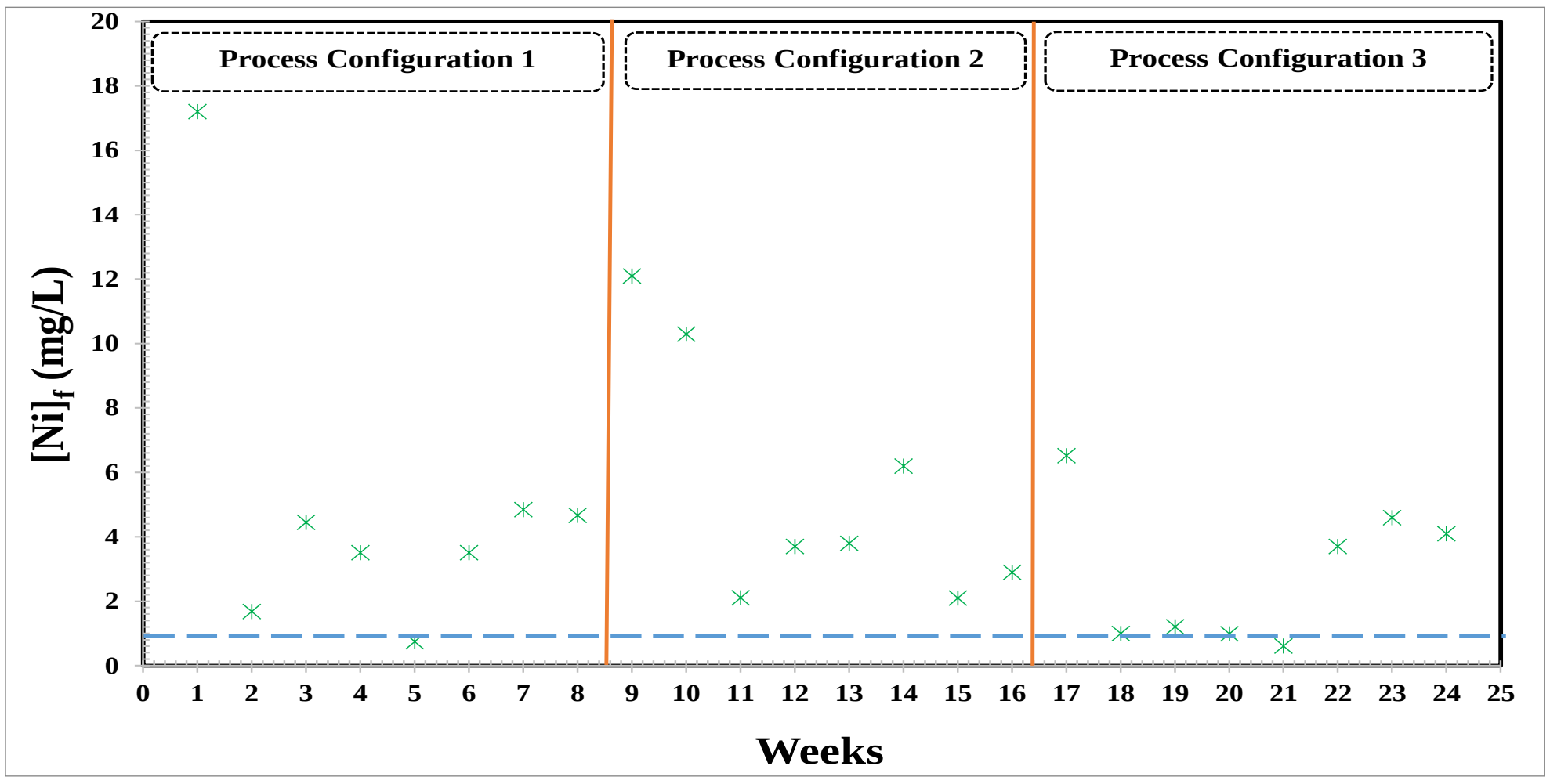

Fig. 9. Nickel concentration in f-EPW: process stability assessment over 24 weeks.

3.1.2. Economic Feasibility and Cost-Effectiveness Analysis

The economic feasibility of industrial wastewater treatment processes is a key criterion for technology selection because OCs directly affect long-term sustainability. OCs generally include energy consumption, chemical reagents, equipment maintenance, and sludge management **[1]**.

In this comparative assessment, all three configurations operated under identical hydraulic conditions using the same pumping, mixing, and solid–liquid separation equipment. Consequently, energy consumption and routine maintenance requirements were assumed equivalent, making chemical consumption the primary differentiating cost factor. As summarized in Table 3, the economic comparison therefore focused on the specific chemical OC (C_{chem}, €·m^{-3}) per cubic meter of treated wastewater, including all reagents—coagulants, pH adjustment chemicals, AF, and polymer flocculant.

Configuration 1, based on $FeCl_3$ coagulation, had the highest OC at 0.432 €·m^{-3}, primarily due to the relatively high unit cost of $FeCl_3$ and the large dosages required to achieve acceptable treatment performance. Although widely used industrially, this conventional approach becomes economically unfavorable for high-strength EPW, where chemical consumption dominates operating expenses.

Configuration 2, using PAC as the primary coagulant, reduced chemical costs to 0.303€·m^{-3}, representing approximately 30% savings compared with Configuration 1. This reduction stems from PAC's higher coagulation efficiency, which allows lower reagent dosages while maintaining comparable performance. As a result, it is a cost-effective intermediate solution without major process modifications.

Process Configuration 3, based on lime-driven chemical attack, achieved the lowest cost at 0.134 €·m^{-3}, corresponding to approximately 69% and 56% reductions compared to Configurations 1 and 2, respectively. This economic advantage stems from the multifunctional role of LS, which simultaneously enables pH adjustment, metal precipitation, and coagulation, thereby eliminating the need for multiple chemical inputs. Additionally, the wastewater's intrinsic acidity proved sufficient for the chemical attack stage, avoiding the need for supplementary acid. These features demonstrate that optimized reaction pathways can deliver both superior treatment efficiency and substantial cost savings.

Beyond direct chemical costs, Configuration 3 offers additional economic benefits in sludge management. The literature indicates that coagulation-flocculation processes produce sludges

with greater volume, poorer dewaterability, and inferior quality compared with lime precipitation **[35]**.

Coagulant-based sludges contain significant residual polymers and metal-organic complexes, increasing volume and complicating disposal **[2,36]**. Conversely, lime precipitation produces predominantly crystalline metal hydroxide sludges with superior settling and dewatering properties, yielding lower dewatered cake volumes and reduced transportation and disposal costs **[35,38]**.

Furthermore, LS shows greater recyclability potential because metal hydroxides can be readily recovered via acidification or thermal treatment [20,38]. Studies confirm that lime precipitation produces more stable sludge with lower HM leaching potential, thereby reducing environmental risks and regulatory compliance costs **[38,39]**.

The nickel-rich sludge from Configuration 3 has notable valorization potential, as recovered nickel can serve as a catalyst in hydrogen production **[41]**. Additionally, the absence of chloride-based coagulants reduces corrosion-related equipment risks, lowering maintenance costs and extending service life **[42]**.

Overall, Configuration 3 is the most cost-effective treatment option, combining the lowest OCs with superior and stable performance. The substantial savings—approaching 70% compared with conventional coagulation—provide strong economic justification for upgrading processes in electroplating facilities. These results show that regulatory compliance, environmental performance, and economic competitiveness can be achieved simultaneously through appropriately designed treatment strategies, supporting the transition toward more sustainable industrial wastewater management practices.

Table 3. Calculating the chemical operating cost (C_{chem}). (AF: antifoaming agent, A-PAM: anionic polyacrylamide, PAC: polyaluminum chloride, LS: lime slurry, D_i: dosage of reagent i, and P_i: unit price of reagent i)

		P_i (€·kg⁻1)	D_i (kg·m⁻³)	C_{chem} (€·m⁻³)
Configuration 1	$FeCl_3$	0.41	0.864	0.432
	H_2SO_4	0.21	0.309	
	AF	16.16	3 E-07	
	A-PAM	1.07	0.016	
Configuration 2	PAC100	0.28	0.62	0.303
	H_2SO_4	0.21	0.309	
	AF	16.16	3 E-07	
	A-PAM	1.1	0.02	
Configuration 3	LS	0.2	0.59	0.134
	AF	16.16	3 E-07	
	A-PAM	1.07	0,016	

3.2. Optimization of the Treatment Process Configuration 3

3.2.1. Response Surface Methodology (RSM) and Model Development

Configuration 3 was optimized using RSM coupled with CCD to systematically evaluate the interactive effects of critical operational parameters on nickel removal efficiency. The experimental design matrix, presented in Table 4, includes three independent variables: $[Ni]_i$ in the E3 effluent, solution pH, and [A-PAM].

Experimental data from CCD trials were subjected to regression analysis to develop a predictive model relating operational parameters to the final nickel concentration ($[Ni]_f$) in the treated effluent. Design Expert software was used for statistical analysis and model development. The relationship between the independent variables and the response was adequately described by a second-order polynomial equation, indicating significant curvature and interaction effects within the experimental domain. Equations (2) and (3) represent the model in coded and actual terms, respectively, enabling both comparative factor assessment and practical application for process control.

Coded form: $Y = 0.5859 + 1.07A - 4.2B - 0.3301C - 1.34AB + 0.1783AC + 0.735BC + 0.6423A^2 + 3.98B^2 + 0.1205C^2$ **(2)**

Actual form: $[Ni]_f = 293.49 + 0.273[Ni]_i - 68.34pH - 3.35[A\text{-}PAM] - 0.034[Ni]_i \cdot pH - 0.002[Ni]_i \cdot [A\text{-}PAM] + 0.368pH \cdot [A\text{-}PAM] + 0.000422[Ni]_i^2 + 3.98pH^2 + 0.030[A\text{-}PAM]^2$ **(3)**

where: Y represents the coded response, $[Ni]_f$ denotes the final nickel concentration (mg/L), $[Ni]_i$ represents the initial nickel concentration (mg/L), pH indicates the solution pH, and [A-PAM] signifies the anionic polyacrylamide dosage (mg/L). The coded equation facilitates direct comparison of factor effects by normalizing coefficients to dimensionless units, while the actual equation enables practical prediction of effluent quality under specified operating conditions.

Coefficient analysis reveals how factors influence nickel removal. The large negative coefficient for pH (-4.20 in coded form) confirms that increasing pH within the experimental range substantially reduces $[Ni]_f$, consistent with enhanced hydroxide precipitation at elevated pH. The positive quadratic pH coefficient (3.98) indicates a minimum on the response surface, suggesting an optimal pH beyond which further increases may reduce removal efficiency, potentially due to hydroxide resolubilization at extremely alkaline conditions or the formation of soluble hydroxo-complexes. The negative AB interaction coefficient (-1.34) indicates that the beneficial effect of pH on nickel removal diminishes at higher $[Ni]_i$, suggesting saturation of precipitation capacity or kinetic limitations under elevated metal loading. Conversely, the

positive BC interaction coefficient (0.735) shows that flocculant effectiveness increases at higher pH, likely reflecting enhanced floc aggregation and the settling characteristics of nickel hydroxide precipitates formed under more alkaline conditions.

Table 4. Experimental design matrix and response. ($[Ni]_i$: Nickel concentration before treatment; [A-PAM]: anionic polyacrylamide dose; $[Ni]_f$: Nickel concentration after treatment)

Run	A: $[Ni]_i$ (ppm)	B: pH	C: [A-PAM] (ppm)	A	B	C	$[Ni]_f$ (ppm)	
							Actual value	Predicted value
1	-1	-1	-1	45	7	4	8.61	9.51
2	1	-1	-1	80	7	4	12.53	12.14
3	-1	1	-1	45	9	4	0.60	0.12
4	1	1	-1	80	9	4	0.37	0.35
5	-1	-1	1	45	7	8	7.64	7.44
6	1	-1	1	80	7	8	9.50	9.76
7	-1	1	1	45	9	8	0.93	0.99
8	1	1	1	80	9	8	2.02	0.90
9	-1.68	0	0	33	8	6	0.56	0.22
10	1.68	0	0	92	8	6	1.70	2.35
11	0	-1.68	0	62.5	6.318	6	20.3	19.85
12	0	1.68	0	62.5	9.682	6	3.76	4.52
13	0	0	-1.68	62.5	8	2.6	2.01	1.90
14	0	0	1.68	62.5	8	9.36	0.20	0.62
15	0	0	0	62.5	8	6	0.93	0.92
16	0	0	0	62.5	8	6	0.93	0.92

3.2.2. Statistical Analysis and Model Validation

Analysis of variance (ANOVA) was conducted to assess model significance, adequacy, and predictive capability. The results, summarized in Table 5, show robust model performance with an F-value of 111.37 (p-value < 0.0001), indicating that the probability of such a large F-value arising from random noise is less than 0.01%. This low probability confirms that the developed model captures genuine relationships between operational parameters and nickel removal performance rather than random experimental variation.

Individual term significance analysis showed that pH (B) has the most pronounced effect on nickel removal, with an F-value of 292.64 (p-value < 0.0001). This dominant effect reflects the critical role of pH in governing nickel hydroxide precipitation equilibria, with solubility minima occurring within specific pH ranges. The quadratic pH term (B^2) was also highly significant (F-value = 393.85, p-value < 0.0001), confirming substantial curvature in the pH-response

relationship and indicating an optimal pH region for maximum nickel removal. The interaction terms AB ($[Ni]_i$ × pH, F-value = 5.70, p-value = 0.044) and BC (pH × flocculant dosage, F-value = 8.48, p-value = 0.020) were statistically significant, showing that the influence of pH on nickel removal varies with both influent metal loading and polymer dosing rate. These interactive effects underscore the complexity of the treatment system and justify the use of RSM rather than traditional one-factor-at-a-time approaches.

$[Ni]_i$ (A) and flocculant dosage (C) did not show individual statistical significance as main effects (p-value > 0.05), suggesting that their effects are mediated primarily through interactions with pH rather than as independent factors. This observation aligns with fundamental precipitation chemistry, where pH predominantly controls metal speciation and hydroxide formation **[2]**, while influent concentration and flocculant dosage modulate the extent and efficiency of these pH-dependent processes. The lack of significance for certain terms does not diminish their inclusion in the model, as they contribute to hierarchical integrity and support significant interaction terms.

Table 5. Analysis of variance (ANOVA) results. (MS: Mean Square, DF: Desirability function, p-value: Probability value, F-value: Fisher value)

Source	DF	MS	F-value	p-value
**Model**	9	56.75	111.37	< 0.0001
**A**	1	1.23	2.40	0.1596
**B**	1	149.11	292.64	< 0.0001
**C**	1	0.9219	1.81	0.2155
**AB**	1	2.90	5.70	0.0440
**AC**	1	0.0512	0.1005	0.7594
**BC**	1	4.32	8.48	0.0195
**A^2**	1	0.2116	0.4153	0.5373
**B^2**	1	200.68	393.85	< 0.0001
**C^2**	1	0.1837	0.3604	0.5649

Model adequacy was rigorously evaluated using multiple statistical metrics (Table 6). The correlation coefficient (R^2 = 0.9917) indicates that 99.17% of the variability in nickel removal is explained by the model, demonstrating an excellent fit to the experimental data. The adjusted R^2 (0.9832) accounts for the number of model terms and confirms that the high R^2 is not artificially inflated by excessive parameters. The predicted R^2 (0.9352) reflects the model's ability to predict responses for new observations, and its reasonable agreement with the adjusted

R^2 (difference < 0.2) indicates the absence of overfitting and validates the model's robustness for predictive applications beyond the experimental dataset. The adequate precision ratio of 37.08, substantially exceeding the desirable threshold of 4, indicates an excellent signal-to-noise ratio and confirms that the model can reliably distinguish true effects from experimental error across the design space.

Table 6. Statistical summary of the developed model. (R^2: Correlation coefficient; R^2adj: Adjusted R^2; R^2pred: Predicted R^2)

Response	R^2	$Radj^2$	R_{pred}^2	Adequate precision
**$[Ni]_f$ (ppm)**	0.9917	0.9832	0.9352	37.0842

Fig. 10 shows a strong correlation between predicted and experimental values, with data points clustering tightly around the diagonal line of perfect prediction, visually confirming the model's accuracy and the absence of systematic prediction bias.

The comprehensive statistical analysis establishes the developed RSM model as a reliable and robust tool for optimizing the performance of Configuration 3. The model captures the complex interactions among operational parameters governing nickel removal and provides a quantitative framework for identifying optimal treatment conditions that balance removal efficiency, chemical consumption, and process stability under variable influent characteristics. This optimization approach addresses the critical limitation identified, where fixed operational parameters proved inadequate for accommodating influent variability, by enabling data-driven selection of adaptive operating strategies responsive to real-time changes in wastewater composition.

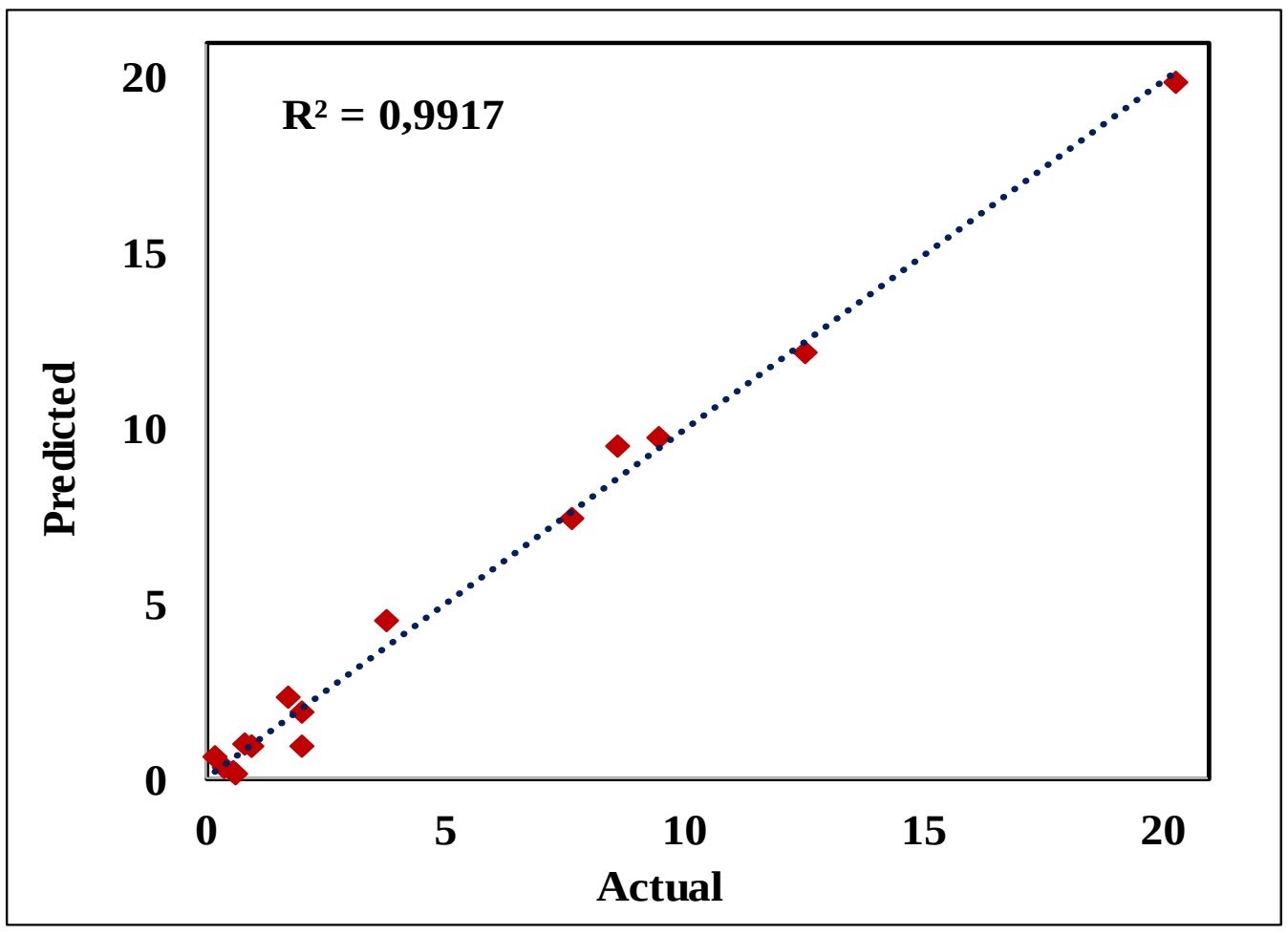

Fig. 10. Predicted vs. experimental values: Final nickel concentration $[Ni]_f$ (ppm).

3.2.3. Interactive Effects Visualization

Three-dimensional (3D) response surface plots (Fig. 11) visualize the complex relationships among operational parameters and nickel removal efficiency, revealing interaction effects and identifying optimal operating regions within the experimental domain.

Fig. 11a shows the response surface for $[Ni]_f$ as a function of $[Ni]_i$ and pH at a constant [A-PAM] of 6 mg/L. The surface exhibits pronounced second-order polynomial curvature with a distinct valley at optimal conditions. pH has a dominant influence (F-value: 292.64), producing a steep gradient from pH 6 to 8-9, confirming its controlling role in nickel hydroxide precipitation kinetics **[1]**.

At pH < 7, insufficient alkalinity results in elevated residual concentrations across all metal loadings, indicating incomplete precipitation. Optimal conditions occur at pH 8-9, where $Ni(OH)_2$ solubility is minimized and precipitation becomes thermodynamically favorable **[13, 33]**.

The surface topology reveals a significant pH/$[Ni]_i$ interaction, as indicated by the negative AB coefficient (-1.34). At moderate loadings (10-30 mg/L), performance shows gradual pH-dependent changes, indicating lower sensitivity to pH optimization. Conversely, at elevated concentrations (60-100 mg/L), steeper pH-dependent gradients show that precise pH control is critical for acceptable effluent quality. Contour projections indicate that achieving $[Ni]_f < 5$ mg/L requires pH > 8 for influents at 80-100 mg/L, whereas pH 7-7.5 suffices for $[Ni]_i < 50$ mg/L, providing guidance for adaptive process control strategies.

Fig. 11b shows the response surface for $[Ni]_f$ as a function of pH and [A-PAM] at a constant $[Ni]_i$ (80 mg/L). The surface shows pH dominance, with [A-PAM] effects becoming significant only under favorable precipitation conditions. At pH ≤ 7, the surface remains elevated ($[Ni]_f >$ 15 mg/L) regardless of flocculant dosage, confirming that insufficient alkalinity prevents effective treatment and that increased polymer addition cannot compensate for thermodynamically unfavorable conditions **[33]**.

As pH increases to 7.5-8, the surface descends with increased curvature along the [A-PAM] axis, indicating the growing importance of flocculant optimization. The optimal [A-PAM] region centers at 6-8 mg/L, where polymer-enhanced flocculation promotes aggregation and settling of colloidal hydroxide precipitates. Excessive dosing (>10 mg/L) yields diminishing returns and may impair performance through charge reversal or floc destabilization.

At pH ≥ 8.5, the response surface reaches its minimum ($[Ni]_f <$ 2-3 mg/L) with relatively gentle gradients, indicating operational robustness and reduced sensitivity to [A-PAM] variations.

This resilience offers practical advantages by tolerating influent variability without compromising effluent quality. However, the positive B^2 coefficient (3.98) warrants caution at pH > 9.5 to avoid potential hydroxide resolubilization under excessively alkaline conditions.

Fig. 11c shows response surface slices at fixed pH values (6, 7, 7.5, and 9), illustrating how the $[Ni]_i$/[A-PAM] interaction evolves across pH conditions. At pH 6, the nearly planar surface topology ($[Ni]_f$ = 20-35 mg/L) indicates minimal interaction between variables, as both factors exert negligible influence when pH-controlled precipitation is fundamentally constrained.

At pH 7, the surface begins to exhibit curvature at $[Ni]_f$ = 6-15 mg/L, indicating emerging sensitivity to both parameters as hydroxide precipitation becomes thermodynamically viable.

At pH 7.5, increased curvature indicates pronounced $[Ni]_i$/[A-PAM] interaction, and the optimal flocculant dosage shifts slightly higher at elevated metal loadings to accommodate increased solids content and maintain effective flocculation **[38]**.

At pH 9, the surface maintains consistently low residual concentrations ($[Ni]_f$ < 3 mg/L) across the entire $[Ni]_i$/[A-PAM] domain, confirming that optimal pH conditions provide the thermodynamic foundation for effective treatment. The comprehensive analysis shows that maintaining pH 8.5-9.0 with [A-PAM] at 6-7 mg/L for influents at 80 mg/L consistently achieves removal efficiency > 97.5%, ensuring regulatory compliance.

These findings suggest that pH set-points and flocculant dosing rates should be dynamically adjusted in response to variations in influent concentration to maintain optimal performance in post-dechromatation EPW treatment **[2]**.

As shown in Fig. 11c, the response surface slices clearly show that increasing pH shifts the system from load-sensitive to load-robust, enabling stable nickel removal across a wide range of influent variability when pH ≥ 7.5.

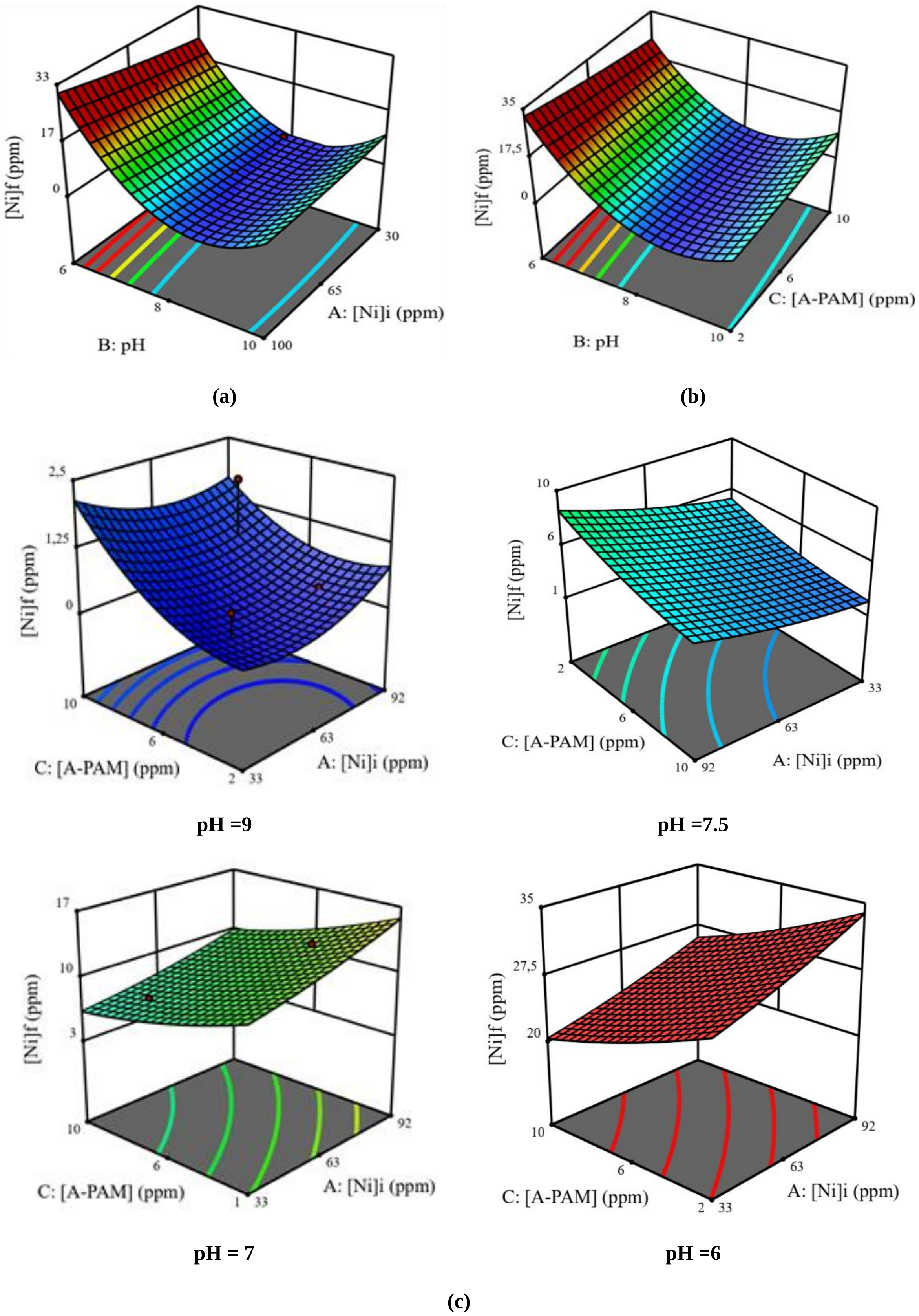

Fig. 11. Three-dimensional (3D) response surface plots for optimizing nickel removal: (a) effect of $[Ni]_i$ and pH at [A-PAM] = 6 ppm; (b) effect of pH and [A-PAM] at $[Ni]_i$ = 80 ppm; and (c) effect of $[Ni]_i$ and [A-PAM] at pH 6, 7, 7.5, and 9.

3.2.4. Model Validation Under Industrial Conditions

To evaluate the predictive reliability of the developed RSM-CCD model, validation was performed using real EPW samples. This strategy was designed to assess the model's accuracy and robustness under actual industrial conditions, which are characterized by natural fluctuations in influent composition.

The influent nickel concentration was first determined experimentally through laboratory analysis. Based on this measured input, the optimal operating conditions—specifically pH and [A-PAM]—were calculated using the established predictive model. The treatment was then applied under these model-recommended conditions, and the resulting effluent nickel concentration was measured to verify the model's predictions.

The results in Table 7 show strong agreement between predicted and experimentally measured effluent nickel concentrations. For an influent concentration of 62 ppm, the model predicted a residual nickel concentration of 0.822 ppm, while the measured value was 0.91 ppm, yielding a prediction error of 9.67%. This deviation remains within acceptable limits for industrial wastewater treatment applications, confirming the model's predictive accuracy.

These findings confirm that the developed optimization model maintains reliable performance when applied to industrial wastewater samples. The model's ability to accurately predict treatment outcomes under real-world operating conditions demonstrates its practical applicability for full-scale implementation and process control.

Table 7. Validation of the Optimization Model Under Real Industrial Conditions. (A-PAM: Anionic Polyacrylamide, $[Ni]_f$: Final Nickel Concentration).

Conditions	Response: $[Ni]_f$ (ppm)		Error (%)
	Predicted value	Actual value	
$[Ni]_i$ = 62 ppm<br>pH = 7.96<br>[A-PAM] = 7.94 ppm<br>Desirability = 0.158	0.822	0.91	9.67

3.3. Innovation and Comparative Performance of Electroplating Wastewater (EPW) Treatment Systems to the Literature

As shown in Table 8, the comparative assessment places the present study within the broader landscape of EPW treatment technologies, highlighting critical trade-offs among performance, economic viability, and operational complexity. A distinguishing feature of this work is its full-

scale industrial validation over 24 weeks under real process conditions, in contrast to predominantly laboratory-scale investigations **[7, 10, 14]**, where treatment durations rarely exceed several days under controlled conditions. While laboratory studies consistently report removal efficiencies of 95-99%, the present study's extended dataset provides empirical evidence that optimized lime-based precipitation maintains high removal efficiencies (99% for Cr, 97% for Ni) despite compositional fluctuations, demonstrating operational resilience that laboratory investigations cannot adequately assess.

The comparison with Gao et al. **[1]**, who operated a sophisticated eight-stage system at full scale, is particularly instructive. Despite automated controls and cascaded treatment stages, their facility experienced persistent instability, and treated wastewater quality frequently failed to meet standards due to improper dosing and unreliable monitoring. This underscores that system complexity does not guarantee treatment stability. Configuration 3's streamlined approach—achieving superior performance through optimized chemistry rather than advanced processes—demonstrates that robust treatment depends fundamentally on controlling chemical mechanisms rather than layering multiple unit operations.

Configuration 3's chemical cost of 0.134 €/m³ represents approximately a 69% reduction relative to conventional $FeCl_3$-based treatment, positioning lime precipitation among the most cost-effective options reported. This advantage stems from lime's multifunctional role—simultaneously enabling pH adjustment, metal precipitation, and coagulation—thereby eliminating the need for multiple specialized reagents characteristic of conventional systems.

Comparison with Gao et al.'s **[1]** operation, which reports total costs exceeding 7.80 €/m³, underscores the economic burden of process complexity. Their elevated costs stem from multiple chemical additions across eight stages, plus substantial sludge management expenses (4.97 €/m³)—a consequence of coagulant-polymer precipitation that generates voluminous, poorly dewatering sludges. Lime-based systems produce crystalline metal hydroxide sludges with superior settling properties, as corroborated by Bhagavath et al. (2021) **[33]**, who documented sludge volume reduction when replacing alum with lime combinations.

Advanced technologies offer specific advantages while introducing distinct barriers. Tang et al.'s **[12]** photovoltaic-powered electrocoagulation (EC) demonstrates exceptional removal (>98%) at low cost (~0.019 €/m³), but requires substantial capital investment, electrode maintenance (cleaning every 7 days), and the addition of conductive salt.

Hybrid membrane processes require sequential operations with extended treatment times (240 minutes) and complex protocols, and are viable only for high-value metal recovery **[25]**. These comparisons show that advanced technologies, despite impressive laboratory performance,

introduce operational complexities and economic burdens that may be prohibitive for typical industrial applications.

A fundamental limitation of many studies is reliance on synthetic effluents, which systematically overestimate industrial performance. The present study's treatment of authentic post-dechromatation wastewater—with variable nickel (33-92 mg/L), residual chromium, competing metals, organic complexants, and elevated chloride—provides a realistic assessment. Bhagavath et al. **[33]** achieved 90-91% nickel removal from synthetic solutions but observed significant degradation with real effluents exceeding 1000 mg/L Ni, necessitating dilution. Identification of metal-ligand complexation, chloride interference, and fixed-dosing limitations highlights challenges common across modalities that are rarely addressed in laboratory studies, underscoring that success depends on understanding complex matrices rather than technology selection alone.

The comparative analysis validates the present approach as scientifically rigorous, economically competitive, and industrially viable. Configuration 3's combination of high efficiency, 24-week operational stability, and exceptional cost-effectiveness positions it favorably against both conventional multi-stage systems and emerging technologies. The substantial improvements achieved through systematic optimization of established chemical treatment—rather than complex emerging technologies—demonstrate that significant advances remain possible within proven frameworks. Future progress depends on intelligent process design, full-scale validation, and adaptive optimization through real-time sensing and automated dosage adjustment—building incrementally on fundamental principles that serve industrial practice effectively.

Table 8. Comparison of literature-reported Electroplating Wastewater (EPW) treatments with the present full-scale study. (PC: Physicochemical; C1, C2, C3: Configuration 1, 2, 3; $FeCl_3$: Ferric chloride; PAC: Polyaluminum chloride; PAM: Polyacrylamide; NaClO: Sodium hypochlorite; Na_2S: Sodium sulfide; $FeSO_4$: Ferrous sulfate; Cr: Chromium; Ni: Nickel; Cu: Copper; Zn: Zinc; Cl^-: Chloride; COD: Chemical oxygen demand; NTU: Nephelometric turbidity unit; EPW: Electroplating wastewater; PVC: Polyvinyl chloride; UBSB: biochar; PE-SACC: Photovoltaic energy-sinusoidal alternating current coagulation; AC: Alternating current; DCC: Direct current control; EEC: Energy consumption; EO: Electrooxidation; Ti/TiO_2-GO-SnO_2: Titanium/titanium dioxide-graphene oxide-tin dioxide composite electrode; SS: Stainless steel; AGMD: Air gap membrane distillation; SLM: Supported liquid membrane; ED: Electrodialysis; RSM: Response surface methodology; CCD: Central composite design; R^2: Coefficient of determination; Lab: Laboratory scale).

**Ref.**	**Scale**	**Treatment**	**Key Pollutants — Influent→ Effluent (mg/L) (Removal)**	**Cost (€/m³)**	**Remarks**
**This study**	Full-scale + Lab	PC— Multi-stage 3 configurations: C1: FeCl, C2: PAC and C3: Lime slurry	Cr: 70–132 → 0.1–1.03 (99%)<br>Ni: 33–92 → 0.61–4.6 (97%)<br>Cl^-: 1171–1418 → 588–792 (50%)	C1 : 0.432<br>C2 : 0.303<br>C3 : 0.134	RSM–CCD model (R^2=0.9917); adaptive dosing; best cost-efficiency
**[1]**	Full-scale	PC — Multi-stage (PAC/PAM, NaClO, Na_2S, $FeSO_4$)	Cr: 4–332 → 0.53–7.61 (up to 99.8%)<br>Ni: 20–499 → variable (40–90%)<br>COD: 1000–2500 → 400–1000 (24–79%)	Chem: ~2.05 / Sludge: ~4.97	Inconsistent performance; very high
**[33]**	Lab + Industrial	PC — Coagulation with *A. squamosa* biomass	Ni: 200–1700 → ~87.6 (90–91%)<br>Turbidity: → 60–64 NTU (>98%)	Low (expected)	Green coagulant; sludge reduced 4× vs. alum
**[10]**	Lab	Subcritical hydrothermal + Redox + Adsorption (PVC co-treatment)	Ni^{2+}: real EPW → ~0.03% residual (99.97%)	—	Exceptional removal; dual-waste valorization; not upscaled
**[7]**	Lab	Adsorption — UBSB biochar	Cr: 0.44 → ~0 (100%)<br>Ni: 10.1 → variable (variable)<br>Cu: 36.7 → ~0 (90–100%)	Low (expected)	Real EPW tested; long contact time; variable Ni/Zn
**[12]**	Lab + Pilot	Electrochemical — PE-SACC (sinusoïdal AC, Fe electrodes)	Cu: 50 → 0.12 (99.3%)<br>Zn: 50 → 0.63 (99.1%)<br>Ni: 50 → 0.08 (98.4%)	~0.019 (EEC: 0.105 kWh/m³)	−37% energy vs. DCC; −67% sludge; low COD removal
**[14]**	Lab	Electrochemical — EO (Ti/TiO_2-GO-SnO_2 electrode)	Cr(VI): 100 → 2.45 (97.6%)<br>Zn: 100 → 4.34 (95.7%)<br>COD: 800 → 101 (87.3%)	~1.12 (14.97 kWh/m³)	Novel electrode; high energy cost; continuous mode viable
**[25]**	Lab	Membrane hybrid — AGMD + SLM + ED	Ni: real EPW → concentrated/recovered AGMD (99.4%) · SLM (76%) · ED (81%)	—	Ni *recovery* focus; complex multi-stage; not yet upscaled

4. Conclusions

This comprehensive investigation of electroplating wastewater (EPW) treatment has demonstrated that strategic process reconfiguration, coupled with variability-responsive statistical optimization, can simultaneously deliver superior treatment performance, enhanced operational stability, and exceptional economic efficiency.

Through systematic evaluation of three physicochemical treatment configurations over 24 weeks of full-scale industrial operation, the lime slurry-based chemical attack process (process configuration 3) proved optimal, achieving 99% chromium removal, 97% nickel removal, and 50% chloride reduction, while lowering operating costs by 69% compared with conventional $FeCl_3$ treatment (0.134 vs. 0.432 €/m³). The mechanistic advantages of this configuration include effective dissociation of refractory metal-ligand complexes via sequential acidification-neutralization, enhanced hydroxide precipitation kinetics under alkaline conditions, and elimination of supplementary chloride inputs that constrain conventional coagulation systems.

The principal innovation of this research is a variability-responsive optimization methodology that explicitly incorporates influent nickel concentration fluctuations (33-92 mg/L) as an independent design factor within the Response Surface Methodology-Central Composite Design framework. Implemented for the first time in EPW treatment, this approach generated a robust second-order polynomial predictive model ($[Ni]_f = f([Ni]_i$, pH, [A-PAM])) with exceptional accuracy ($R^2 = 0.9917$, adjusted $R^2 = 0.9832$). The model quantifies how optimal pH requirements shift systematically with influent metal loading, driven by significant interaction effects (F-value = 5.70, p-value = 0.044), thereby validating that static optimization approaches are inadequate for accommodating industrial wastewater variability. Critically, the derived mathematical relationship enables real-time adaptive chemical dosing, allowing operators to dynamically calculate optimal pH targets (8.5-9.0) and flocculant requirements (6-7 mg/L) based on measured influent composition. Under model-predicted conditions, residual nickel consistently remained below 2 mg/L across the entire experimental domain, ensuring regulatory compliance despite extreme compositional fluctuations.

The practical advantages of this integrated approach include: (1) transforming treatment from an empirical operation to quantitative, model-guided control, (2) minimal capital requirements by using basic online monitoring rather than expensive advanced control systems, (3) substantial cost reduction through optimized chemical use, (4) enhanced process robustness against influent variability, and (5) improved environmental performance through chloride

minimization and consistent regulatory compliance. These benefits position the optimized lime-based process as a technically superior and economically viable alternative to conventional EPW treatment methodologies.

Several limitations merit acknowledgment. The optimization framework focused exclusively on nickel as the primary response variable, whereas a simultaneous multi-objective optimization that considers chromium, copper, and zinc removal could reveal additional opportunities for performance improvement. The predictive model was developed using batch-scale experiments under controlled conditions; comprehensive validation through extended full-scale implementation with automatic adaptive dosing would confirm industrial robustness and identify potential scale-dependent effects. Economic analysis was restricted to chemical operating costs, excluding energy consumption, sludge disposal expenses, and capital investment requirements necessary for a complete life-cycle cost assessment.

Future research should prioritize: (1) expanding the variability-responsive optimization approach to capture simultaneous fluctuations of multiple metal species using multidimensional experimental designs, (2) integrating machine learning algorithms that automatically refine predictive relationships from historical performance data, (3) conducting systematic validation under full-scale industrial conditions that include extreme operational disturbances and prolonged monitoring periods, (4) performing a comprehensive techno-economic analysis that includes life-cycle costs and environmental impacts, (5) investigating model extrapolation boundaries and developing recalibration protocols for long-term reliability, and (6) characterizing sludge properties and valorization potential under adaptive dosing conditions.

Beyond its immediate application to EPW, the variability-responsive optimization methodology is a generalizable framework with transformative potential across diverse industrial sectors characterized by compositional variability, including metal finishing, mining, pharmaceutical manufacturing, and food processing. The principle that optimization should embrace rather than avoid influent variability by treating it as an explicit design factor represents a paradigm shift in industrial wastewater treatment. As water scarcity intensifies and discharge regulations tighten globally, this integrated approach—combining advanced process chemistry, statistical optimization, and adaptive control—establishes a new standard for sustainable industrial water management, where environmental performance and economic efficiency are simultaneously optimized rather than perceived as competing objectives.

Acknowledgments

This research was supported and funded by the Deanship of Scientific Research at Imam Mohammad Ibn Saud Islamic University (IMSIU) (grant number IMSIU-DDRSP2602).

References

1. Gao J, Han HL, Zhu J, Zhou J. Navigating challenges in electroplating wastewater management: A study on pollutant removal characteristics and economic impacts by physicochemical treatment. Colloids Surf C Environ Asp. 2025;3:100060. https://doi.org/10.1016/j.colsuc.2025.100060

2.Li Z, Du C. Current status and research trends of electroplating wastewater treatment: Systematic review and bibliometric analysis. Desalination. 2025;613:119004. https://doi.org/10.1016/j.desal.2025.119004

3. Babilas D, Chromikova J, Kopyto D, Leszczynska-Sejda K, Dydo P. Application of electrodialysis enhanced with complex formation integrated with electrolysis for treatment of electroplating wastewaters as a new approach to selective copper recovery. Chem Eng J. 2024; 494:152707. https://doi.org/10.1016/j.cej.2024.152707

4. Huang H, Qi S, Chen Z, Chen W, Su Y, Chen W, et al. N-nitrosamines in electroplating and printing/dyeing industrial wastewater treatment plants: Removal efficiency, environmental emission, and influence on drinking water. Water Res. 2024;255:121537. https://doi.org/10.1016/j.watres.2024.121537

5. Zhang L, Qin L, Ma L, Shen Z, Jin Y, Chen S. Treatment of electroplating wastewater using electrocoagulation and integrated membrane. Water Sci Technol. 2024;89:2538–2557. https://doi.org/10.2166/wst.2024.143

6. Li Z, Bi Y, Li Y, Dong P, Zhang Y, Huang Z, et al. Synergistic benefits of pollution and carbon emissions reduction in the resource utilization of electroplating wastewater. J Environ Chem Eng. 2025;13:117825. https://doi.org/10.1016/j.jece.2025.117825

7. Arriola-Villaseñor E, Ardila AN, Barrera R, Hernández J. Using banana waste biochar for simultaneous removal of heavy metals from raw real wastewater from the electroplating industry. Desalin Water Treat. 2023;314:88–102. https://doi.org/10.5004/dwt.2023.30079

8. Mahmood Z, Yuan Y, Garg S, Chen Y, Li X, Cui T, et al. Boosting adsorption of organically complexed Ni onto thin-layered porous biomass-derived carbon. Water. 2026;18:371. https://doi.org/10.3390/w18030371

9. García-Ávila F, Mayancela-Santander E, Alvarado-Pacheco B, Valdiviezo-Gonzales L, Cadme-Galabay M, Zhindon-Arévalo C, et al. Comparative analysis of coagulants for selective removal of iron and aluminum from galvanic wastewater: A practical and effective approach. Ain Shams Eng J. 2025;16:103393. https://doi.org/10.1016/j.asej.2025.103393

10. Xiu FR, Zhang Y, Du J, Qi Y. Treatment of waste PVC by subcritical nickel-containing electroplating wastewater: Crucial redox effect on PVC dechlorination and nickel removal. J Environ Chem Eng. 2025;13:119050. https://doi.org/10.1016/j.jece.2025.119050

11. Ibrahimi S, Gasmi A, Elboughdiri N, Sassi Aydi S, Ghernaout D, Hannachi A. Nanofiltration and electrocoagulation treatment of Moorish bath wastewater for irrigation purposes: Case study of El-Hamma, southeastern Tunisia. J Water Process Eng. 2025;70:106955. https://doi.org/10.1016/j.jwpe.2025.106955

12. Tang X, Xu T, Hu S, Liu K, Zeng Z, Wu Q, et al. Design and implementation of control system for electroplating wastewater treatment by photovoltaic energy sinusoidal alternating current coagulation technology. Process Saf Environ Prot. 2024;182:405–415. https://doi.org/10.1016/j.psep.2023.12.003
13. Kamar MT, Elattar H, Mahmoud AS, Peters RW, Mostafa MK. A critical review of state-of-the-art technologies for electroplating wastewater treatment. Int J Environ Anal Chem. 2024;104:4143–4176. https://doi.org/10.1080/03067319.2022.2098486
14. Rajoria S, Vashishtha M, Sangal VK. Electroplating wastewater treatment by electro-oxidation using synthesized new electrode: Experimental, optimization, kinetics, and cost analysis. Process Saf Environ Prot. 2024;183:735–756. https://doi.org/10.1016/j.psep.2024.01.023
15. Almazán-Ruiz FJ, Caballero F, Cruz-Díaz MR, Rivero EP, Vazquez-Arenas J, González I. Nickel recovery from an electroplating rinsing effluent using RCE bench scale and RCE pilot plant reactors: The influence of pH control. Chem Eng Res Des. 2015;97:18–27. https://doi.org/10.1016/j.cherd.2015.02.022
16. Song J, Ran B, An Q, Deng S, Feng Z, Zhao B, et al. Dual cross-linked hydrogel microbial reactor: An improved microbial immobilization technology to treat mixed electroplating wastewater. Process Biochem. 2025;150:57–67. https://doi.org/10.1016/j.procbio.2025.01.001
17. Wang J, Guan Y, Guo M, Gao J, Yang M, Liu Y, et al. New insights into the remediation of chromium-contaminated industrial electroplating wastewater by an innovative nano-modified biochar derived from spent mushroom substrate: Mechanisms, batch study, stability and application. Chemosphere. 2024;367:143621. https://doi.org/10.1016/j.chemosphere.2024.143621
18. Scarazzato T, Panossian Z, Tenório JAS, Pérez-Herranz V, Espinosa DCR. A review of cleaner production in electroplating industries using electrodialysis. J Clean Prod. 2017;168:1590–1602. https://doi.org/10.1016/j.jclepro.2017.03.152
19. Karimi S, Barbe S, Engstler R, Davies PA, Hosseinipour E. High-pressure batch reverse osmosis for zero liquid discharge in a Cr(III) electroplating process. Desalination. 2024;580:117479. https://doi.org/10.1016/j.desal.2024.117479
20. Yu XT, Hou Y, Ren XY, Sun CC, Wang MY. Research progress on the removal, recovery and direct high-value materialization of valuable metal elements in electroplating/electroless plating waste solution. J Water Process Eng. 2022;46:102577. https://doi.org/10.1016/j.jwpe.2022.102577
21. Peng C, Song S, Lu S, López-Valdivieso A. Electroplating wastewater treatment through chemical precipitation and electrodialysis. Eur J Miner Process Environ Prot. 2004;4:210–215.
22. Moersidik SS, Nugroho R, Handayani M, Kamilawati, Pratama MA. Optimization and reaction kinetics on the removal of nickel and COD from wastewater from electroplating industry using electrocoagulation and advanced oxidation processes. Heliyon. 2020;6:e03319. https://doi.org/10.1016/j.heliyon.2020.e03319
23. Lin S, Wu D, Yi B, Hu X, Fang L, Yu S, et al. Advanced sequential treatment approach for enhanced recovery in tungsten–nickel electroplating wastewater. J Water Process Eng. 2025;71:107421. https://doi.org/10.1016/j.jwpe.2025.107421
24. Hidalgo LA, Mihaylov L, Croft CF, Nagul EA, Spassov TG, Kolev SD. Development and application of polymer inclusion beads for the recovery of Zn(II) from simulated industrial waste electroplating solutions. Sep Purif Technol. 2025;378:134581. https://doi.org/10.1016/j.seppur.2025.134581

25. Qoriati D, Tsai DW, You SJ, Wang YF. Experimental investigation on synergistic integrated MD-SLM-ED for nickel recovery from electroplating wastewater: Influence of operating parameters and optimization. Chem Eng J. 2025;506:160277. https://doi.org/10.1016/j.cej.2025.160277

26. Gasmi A, Ibrahimi S, Elboughdiri N, Tekaya MA, Ghernaout D, Hannachi A, et al. Comparative study of chemical coagulation and electrocoagulation for the treatment of real textile wastewater: Optimization and operating cost estimation. ACS Omega. 2022;7:22456–22476. https://doi.org/10.1021/acsomega.2c01652

27. Amari A, Aljibori HSS, Algarni Z, Elboughdiri N, Diab MA, Baek KH, et al. A novel S-scheme photocatalyst Fe2O3/Bi2O3/g-C3N4 with enhanced visible-light photocatalytic performance for antibiotic degradation and CO2 reduction: RSM-based optimization. J Ind Eng Chem. 2024;140:599–616. https://doi.org/10.1016/j.jiec.2024.08.002

28. Ibrahimi S, Gasmi N, Ben Jamaa A, Elboughdiri N, Ghernaout D, Bagané M, et al. Response surface methodology optimization of greener cement production: Exploring the effects of Tunisian phosphogypsum and raw meal fineness on energy efficiency and high-performance cement manufacturing. ACS Omega. 2026;11:2851–2867. https://doi.org/10.1021/acsomega.5c08931

29. Baird RB, Eaton AD, Rice EW, editors. Standard Methods for the Examination of Water and Wastewater. 23rd ed. Washington (DC): American Public Health Association; 2017.

30. International Organization for Standardization. Water quality—Determination of the chemical oxygen demand index (ST-COD)—Small-scale sealed-tube method. ISO 15705:2002. Geneva: ISO; 2002.

31. Kumar V, Dwivedi SK. A review on accessible techniques for removal of hexavalent chromium and divalent nickel from industrial wastewater: Recent research and future outlook. J Clean Prod. 2021;295:126229. https://doi.org/10.1016/j.jclepro.2021.126229

32. Abou-Elela SI, El-Kamah HM, Aly HI. Waste minimization in the electroplating industry (case study). Int J Environ Stud. 1998;55:287–296. https://doi.org/10.1080/00207239808711186

33. Bhagavath R, Subrahmanian S, Narendrakumar G. Enhanced removal of Ni(II) from electroplating effluents using herbal biomass as alum substituents. Desalin Water Treat. 2021;244:241–252. https://doi.org/10.5004/dwt.2021.27912

34. Lee CS, Robinson J, Chong MF. A review on application of flocculants in wastewater treatment. Process Saf Environ Prot. 2014;92:489–508. https://doi.org/10.1016/j.psep.2014.04.010

35. Aguilar MI, Sáez J, Lloréns M, Soler A, Ortuño JF, Meseguer V, et al. Improvement of coagulation–flocculation process using anionic polyacrylamide as coagulant aid. Chemosphere. 2005;58:47–56. https://doi.org/10.1016/j.chemosphere.2004.09.008

36. Amuda OS, Amoo IA, Ipinmoroti KO, Ajayi OO. Coagulation/flocculation process in the removal of trace metals present in industrial wastewater. J Appl Sci Environ Manag. 2006;10:159–162. https://doi.org/10.4314/jasem.v10i3.17339

37. Abdul Fattah AB, Azhar AH. Treatment of automotive wastewater by coagulation-flocculation using poly-aluminum chloride (PAC), ferric chloride (FeCl3) and aluminum sulfate (alum). AIP Conf Proc. 2013;1571:524–531. https://doi.org/10.1063/1.4858708

38. Chen Q, Yao Y, Li X, Lu J, Zhou J, Huang Z. Comparison of heavy metal removals from aqueous solutions by chemical precipitation and characteristics of precipitates. J Water Process Eng. 2018;26:289–300. https://doi.org/10.1016/j.jwpe.2018.11.003

39. Nordkalk. Water treatment—Lime products for water and wastewater treatment. Available from: https://www.nordkalk.com/water-treatment-with-lime-products/. Accessed 1 Jan 2023.

40. Kavaliauskas Ž, Blažiūnas G, Šajev I, Iljinas A, Gimžauskaitė D. Development and optimization of an automated industrial wastewater treatment system using PLC and LSTM neural network. Appl Sci. 2025;15:8990. https://doi.org/10.3390/app15168990

41. Adeniyi AG, Ighalo JO. A review of steam reforming of glycerol. Chem Pap. 2019;73:2619–2635. https://doi.org/10.1007/s11696-019-00840-8

42. Revie RW, Uhlig HH. Corrosion and Corrosion Control: An Introduction to Corrosion Science and Engineering. 4th ed. Hoboken (NJ): John Wiley & Sons; 2008.
