# Supplementary material for: Integrated optimization of electroplating wastewater treatment: A comparative assessment of physicochemical treatment configurations and process enhancement using response surface methodology
Source: PLoS One. 2026 Aug 21;21(8):e0345965. doi: 10.1371/journal.pone.0345965 (PMC13496402; doi:10.1371/journal.pone.0345965)
Supplement: Supplementary file 2 [file pone.0345965.s003.docx]

This manuscript presents a comparative evaluation of physicochemical treatment configurations for electroplating wastewater and introduces an RSM-based optimization framework incorporating influent variability. The work is relevant and practically valuable, especially due to its full-scale industrial dataset and cost analysis. However, the manuscript requires **major revisions,** particularly in the mechanistic interpretation of treatment processes and discussion of human/environmental implications.

Address the following comments:

1. What are the discharge limits for the concerned pollutants and mention standards in the introduction?
2. What operating conditions critically influence the precipitation process?
3. State some of the previous RSM related studies for industrial effluents, their major outcomes and then correlate with the research gaps.
4. The manuscript consists of many short paragraphs. Authors are requested to properly format and improve the scientific language of manuscript.
5. TSS removal exhibits extreme variability (2-118 mg/L in f-EPW vs. 2 ± 0.3 mg/L in E3), why there is standard deviation missing at some places
6. Authors are requested to discuss the mechanism in detail for metal speciation and precipitation chemistry in the manuscript
7. Electroplating wastewater contains strong chelating agents such as EDTA, surfactants. How the mentioned coagulants break the metal-organic complexes and their kinetics effect the precipitation process?
8. The coefficients from the model should be more related to real chemistry
9. Why are emerging contaminants discussed only in the introduction but not in results and discussion?
10. The PC parameters used to evaluate the performance of the EPW investigated in this study were systematically analyzed using standardized and validated analytical methods. Mention the references or cite it
11. The manuscript report ~50% chloride removal which is unlikely as chloride mostly remain soluble. Explain it and perform the mass balance.
12. Unit and notations should be consistent
13. Some figures don’t have error bars
14. Discuss about sludge management, recyclability and how it effect the economic aspect
15. Can authors explain the superiority of lime treatment over current research trends such as adsorption, electrocoagulation, and membrane processes
16. The manuscript reports a significant quadratic effect of pH on nickel removal. Experimentally validate it by evaluating residual Ni over pH range to identify minimum solubility region, re-dissolution at higher pH
17. Correlate RSM optimum pH with minimum thermodynamic solubility
18. Surface interaction studies can be performed to analyse the role of adsorption alongwith precipitation. Isotherm model can be fitted.
19. Discuss the role of mixing speed during jar test, how it effects the diffusion?
20. Environmental and health implication can be discussed in separate section
