# Supplementary material for: Integrated optimization of electroplating wastewater treatment: A comparative assessment of physicochemical treatment configurations and process enhancement using response surface methodology
Source: PLoS One. 2026 Aug 21;21(8):e0345965. doi: 10.1371/journal.pone.0345965 (PMC13496402; doi:10.1371/journal.pone.0345965)
Supplement: Supplementary file 3 [file pone.0345965.s005.docx]

**Response to Reviewers**

Journal of PLOS ONE | Manuscript No. PONE-D-26-12528

**Dear Editor and Reviewers,**

We thank the Editor and Reviewers for their thorough evaluation of our manuscript and for their valuable comments and constructive suggestions. All feedback has been carefully considered, and the manuscript has been extensively revised in response. These revisions have strengthened the manuscript's scientific rigor, clarity, technical depth, and overall organization.

All modifications in the revised manuscript are marked in red for clarity.

Below, we present a detailed point-by-point response to each comment.

**Responses to the Academic Editor**

**Comment 1. Please ensure that your manuscript meets PLOS ONE's style requirements, including those for file naming.**

**Response:**

The manuscript has been meticulously reformatted to comply with the official PLOS ONE guidelines, including manuscript structure, section organization, figure and table formatting, references, abbreviations, and file naming conventions. The revised version is now fully compliant with all journal formatting requirements.

**Comment 2. Please note that PLOS One has specific guidelines on code sharing for submissions in which author-generated code underpins the findings in the manuscript.**

**Response:**

No custom computational code or proprietary software was developed for this study. Statistical analyses and response surface methodology (RSM) modeling were conducted using the commercially available Design-Expert® software. All experimental data, model equations, operating conditions, and statistical outputs required to reproduce the results are provided in the manuscript and the Supporting Information files.

**Comment 3.** Thank you for stating in your Funding Statement:

**“This research was supported and funded by the Deanship of Scientific Research at Imam Mohammad Ibn Saud Islamic University (IMSIU) (grant number IMSIU-DDRSP2602).“** Please provide an amended statement that declares *all* the funding or sources of support (whether external or internal to your organization) received during this study, as detailed online in our guide for authors at <http://journals.plos.org/plosone/s/submit-now>.  Please also include the statement “There was no additional external funding received for this study.” in your updated Funding Statement.

Please include your amended Funding Statement within your cover letter. We will change the online submission form on your behalf.

Thank you for stating the following financial disclosure:

"This research was supported and funded by the Deanship of Scientific Research at Imam Mohammad Ibn Saud Islamic University (IMSIU) (grant number IMSIU-DDRSP2602)."

Please state what role the funders took in the study.  If the funders had no role, please state: "The funders had no role in study design, data collection and analysis, decision to publish, or preparation of the manuscript."
If this statement is not correct you must amend it as needed.

**Response:**

One of the project requirements is to include the funding statement and acknowledgments exactly as they appear in the manuscript, without any changes. Thank you to keep it as it is (you can refer to this published paper: <https://doi.org/10.1016/j.seppur.2025.133205> Funding Statement/ acknowledgement are the same).

The Funding Statement/ acknowledgement: “This research was supported and funded by the Deanship of Scientific Research at Imam Mohammad Ibn Saud Islamic University (IMSIU) (grant number IMSIU-DDRSP2602). The funder was actively involved in the study design, data collection and analysis, the decision to publish, and the preparation of the manuscript.

**Comment 4.** We note that your Data Availability Statement is currently as follows**: [All relevant data are within the manuscript and its Supporting Information files.]**

Please confirm at this time whether or not your submission contains all raw data required to replicate the results of your study. Authors must share the “minimal data set” for their submission. PLOS defines the minimal data set to consist of the data required to replicate all study findings reported in the article, as well as related metadata and methods (<https://journals.plos.org/plosone/s/data-availability#loc-minimal-data-set-definition>).

For example, authors should submit the following data:

-The values behind the means, standard deviations and other measures reported;
- The values used to build graphs;

- The points extracted from images for analysis.

Authors do not need to submit their entire data set if only a portion of the data was used in the reported study.

If your submission does not contain these data, please either upload them as Supporting Information files or deposit them to a stable, public repository and provide us with the relevant URLs, DOIs, or accession numbers. For a list of recommended repositories, please see <https://journals.plos.org/plosone/s/recommended-repositories>.

If there are ethical or legal restrictions on sharing a de-identified data set, please explain them in detail (e.g., data contain potentially sensitive information, data are owned by a third-party organization, etc.) and who has imposed them (e.g., an ethics committee). Please also provide contact information for a data access committee, ethics committee, or other institutional body to which data requests may be sent. If data are owned by a third party, please indicate how others may request data access.

**Response:**

We thank the editor for this clarification. We confirm that our submission now contains all raw data required to replicate all findings reported in the study. A new Supporting Information file **(S1 Data)** has been added, containing the underlying data. All relevant data for Figures 1, 2, 3, 6, 7, 8, 9, and 12 are now provided. The Data Availability Statement has been updated accordingly to: "**All relevant data are within the manuscript and its Supporting Information files**."

**Comment 5.** Thank you for stating the following in the Acknowledgments Section of your manuscript:

**“This research was supported and funded by the Deanship of Scientific Research at Imam Mohammad Ibn Saud Islamic University (IMSIU) (grant number IMSIU-DDRSP2602).”**

We note that you have provided additional information within the Acknowledgements Section that is not currently declared in your Funding Statement. Please note that funding information should not appear in the Acknowledgments section or other areas of your manuscript. We will only publish funding information present in the Funding Statement section of the online submission form.

**Response:**

One of the project requirements is to include the funding statement and acknowledgments exactly as they appear in the manuscript, without any changes. Thank you to keep it as it is (you can refer to this published paper: <https://doi.org/10.1016/j.seppur.2025.133205> Funding Statement/ acknowledgement are the same).

The Funding Statement/ acknowledgement: “This research was supported and funded by the Deanship of Scientific Research at Imam Mohammad Ibn Saud Islamic University (IMSIU) (grant number IMSIU-DDRSP2602).

**Comment 6.** If the reviewer comments include a recommendation to cite specific previously published works, please review and evaluate these publications to determine whether they are relevant and should be cited. There is no requirement to cite these works unless the editor has indicated otherwise.

**Response:**

The reviewers did not recommend any specific references for citation.

**Responses to Reviewer 1**

We sincerely thank Reviewer 1 for the thorough and constructive evaluation. Reviewer 1's technically insightful comments have substantially strengthened the manuscript's scientific rigor, mechanistic depth, and overall coherence. All revisions in response to Reviewer 1 are marked in **red** in the revised manuscript.

**Comment 1. When coagulation process is discussed, techniques like zeta potential are considered, which is missing as of now. Also, for any coagulation process, turbidity and coagulant dosage curve is important, which is also missing. Also, sludge characterization can be added to solidify your paper. It is suggested to add more data or results which are related to coagulation and flocculation process.**

**Response:**

We thank the Reviewer for these insightful technical comments. We acknowledge that the coagulation and flocculation characterization section required further development, and we have revised the manuscript accordingly. We address each point in detail below.

***Point 1 —*** ***Zeta Potential:***

We concur that zeta potential is a fundamental parameter in coagulation science, as it governs colloidal stability and the charge-neutralization mechanism that determines destabilization efficiency. However, we note that direct zeta potential measurements were not conducted during the full-scale monitoring campaign because online zeta potential analyzers were unavailable at the Tunisian facility.

A dedicated paragraph has been added to the revised manuscript (**page 12, lines 281-291**) to discuss the role of zeta potential in the coagulation process.

"**Coagulation:** The dechromatized E1 effluents are combined with the alkaline E2 streams to produce a mixed effluent that undergoes coagulation. In this step, a chemical coagulant is introduced under rapid agitation to destabilize colloidal particles and suspended matter through charge neutralization and sweep floc formation. The effectiveness of destabilization is monitored using zeta potential measurements, which quantitatively assess the electrokinetic stability of the colloidal suspension. Optimal coagulation is achieved when the zeta potential approaches neutrality (approximately 0 mV), indicating that electrostatic repulsive forces have been sufficiently reduced to permit interparticle aggregation **[30]**. This process facilitates the formation of primary microflocs that act as nucleation sites for subsequent metal hydroxide precipitation, while also reducing turbidity and enhancing the settling characteristics of the treated effluent."

***Point 2 — Turbidity and coagulant dosage curve:***

The initial effluent E3 contains a negligible concentration of Total Suspended Solids (TSS) (2 ± 0.3 mg/L), as shown in **Table 1**. Because turbidity is determined by suspended particulate matter, it is negligible and not a relevant process performance indicator in this context. The primary water quality concerns in E3 are elevated concentrations of heavy metals and chlorides, which exceed regulatory discharge limits.

To further support this justification, Section 2.2 (**pages 14 and 15,** **lines 356-366**) has been revised to explicitly clarify that removal of suspended solids is not the primary challenge for this treatment system. Instead, the assessment focuses on heavy metal removal as the central performance criterion.

"The TSS of the initial effluent E3 is inherently negligible (2 ± 0.3 mg/L), indicating that turbidity, governed by the suspended particulate load, is also negligible and does not serve as a meaningful performance indicator or limiting parameter for this specific industrial wastewater matrix. The observed TSS variability in f-EPW (2 ± 1 – 118 ± 2 mg/L) is attributable to operational inconsistencies in the settling stage rather than to coagulation deficiency, confirming that suspended solids removal is not the primary challenge in this treatment system. In contrast, the most significant deficiencies are associated with HMs and chloride concentrations, which have been identified as the primary limiting factors for regulatory compliance and environmental impact. These parameters, therefore, constitute the central focus of the comparative performance assessment in this study."

***Point 3 —*** ***Sludge Characterization:***

We acknowledge that comprehensive quantitative sludge characterization was not conducted during the monitoring campaign. Nevertheless, preliminary field observations indicated that lime-based treatment (Configuration 3) produced higher sludge volumes due to calcium-based byproducts (CaCO₃, Ca(OH)₂). This configuration also showed improved settling behavior, increased dry solids content, and enhanced dewaterability, which partially offset disposal costs.

A dedicated paragraph summarizing these observations has been added to the new section "**4. Environmental implications and research perspectives**," which recommends standardized sludge characterization as future work. This paragraph is marked in red in the revised manuscript (**pages 68-69, lines 1662-1674**) (see also Response to Reviewer 2, Comment 14). Additionally, a preliminary discussion appears on **pages 42-43** (**lines 1039-1056**) and is marked in red in the revised manuscript.

**Comment 2. How is this method novel?**

**Response:**

The innovation lies not in the individual techniques employed (RSM, coagulation, flocculation, precipitation), which are well established, but in reframing the optimization problem conceptually.

Conventional RSM-based studies treat influent composition as a fixed parameter, optimizing treatment conditions for a single, predefined wastewater quality. In contrast, this study introduces influent nickel concentration as an independent variable in the RSM-CCD design, yielding a predictive model of the form [Ni]_f_ = f([Ni]_i_, pH, [A-PAM]). This model dynamically calculates optimal dosing based on the measured influent load, enabling real-time adaptive process control. The statistical identification of a significant pH–metal loading interaction (*F* = 5.70, *p* = 0.044) provides quantitative evidence that optimal parameters shift with influent composition. This variability-responsive framework, validated over 24 weeks at full industrial scale, represents a methodological advance beyond static laboratory optimizations.

**Comment 3. How this sentence is connected to the previous lines? how this is problematic in terms of the heavy metal?**

**Response:**

We acknowledge that the transition between the toxicological characterization of Cr(VI) and Ni(II) and the subsequent discussion of chloride-based coagulants was insufficiently explicit. The intended connection is as follows: after establishing the need to remove these heavy metals, the text addresses the persistent challenges of their removal. Specifically, chloride-based coagulants such as FeCl₃, although effective for metal precipitation, introduce secondary chloride contamination that can violate discharge standards.

The paragraph has been restructured into two distinct sections: one establishing the toxicological rationale and another outlining the technical limitations. This revision is presented on **pages 7 and 8 (lines 172-183)** of the revised manuscript (marked in red). The revised paragraph is as follows:

" Among heavy metal (HM) contaminants, hexavalent chromium (Cr(VI)) and divalent nickel (Ni(II)) are of particular concern because of their extensive use in electroplating and well-documented toxicological effects **[7]**. Cr(VI) is associated with carcinogenicity and acute ecotoxicity **[2]**, whereas nickel is linked to carcinogenic, allergenic, and chronic ecological effects **[8]**.

Consistent removal of these contaminants by conventional physicochemical treatments remains technically challenging for two primary reasons. First, chloride-based coagulants such as ferric chloride (FeCl₃), commonly used for heavy metal precipitation, introduce additional chloride into treated effluents. This can result in chloride concentrations exceeding discharge standards and limit opportunities for water reuse **[2,9]**. Second, strong metal–organic complexes formed by plating additives can inhibit standard precipitation mechanisms, allowing residual dissolved metals to persist even when chemical dosing and pH are appropriately controlled **[2,10]**."

**Comment 4. NT 106.002 what is this? This is not clear. can you please add more?**

**Response:**

**NT 106.002**, issued by the Tunisian Institute of Standardization (INNORPI), establishes the national regulatory framework for wastewater quality in Tunisia, specifically for discharges into receiving water bodies. This standard sets legally binding maximum permissible limits for various physicochemical parameters in industrial effluents, including heavy metals (chromium, nickel), chlorides, pH, and suspended solids.

The revised manuscript expands the original sentence on **page 8** **(lines 186-190)**, as marked in red. The updated text is as follows:

"Within this regulatory framework, Tunisia has adopted the NT 106.002 standard (INNORPI, Tunisian Institute of Standardization) as a mandatory reference for industrial effluent quality control. The standard sets maximum permissible limits for physicochemical parameters and is particularly applicable to metal-finishing processes, including electroplating **[11]**."

**Comment 5. The paragraph introduces multiple treatment technologies for EPW; however, the subsequent discussion focuses primarily on the limitations of chemical precipitation. It would improve clarity and balance if the authors either briefly discuss the limitations of other listed methods or justify the specific focus on precipitation.**

**Response:**

We agree with this observation. The paragraph has been revised to succinctly acknowledge the primary limitations of each method discussed, thereby providing context and explicitly justifying the emphasis on precipitation-based treatment. This revision appears on **pages 8 and 9 (lines 195-206)** of the revised manuscript (marked in red ):

" Multiple strategies are used to treat EPW, including chemical precipitation **[13]**, coagulation-flocculation **[9]**, electrochemical treatment **[14, 15]**, adsorption **[16,17]**, electrodialysis **[18]**, and membrane filtration **[19]**. Advanced sequential treatment approaches have been developed to address the complexity of EPW pollutants **[20–23]**. Despite these advancements, each technology has practical limitations that restrict its standalone industrial applicability **[24,25]**. Electrochemical methods, although effective, require high energy input and significant capital investment **[14]**. Adsorption processes require expensive regeneration or disposal of saturated media **[16]**. Membrane filtration and electrodialysis are prone to fouling and incur substantial operational costs, especially at elevated pollutant concentrations **[18,19]**. As a result, chemical treatment, particularly precipitation and coagulation-flocculation, remains fundamental to EPW management at the industrial scale due to its demonstrated effectiveness at high pollutant concentrations, operational simplicity, and comparatively low cost **[2]**.

**Comment 6. While the knowledge gaps are clearly articulated, they appear somewhat generic. Supporting these statements with specific literature examples would enhance the scientific rigor and contextual grounding of the discussion.**

**Response:**

We agree with the feedback. The paragraph has been revised to explicitly link each identified gap to specific published evidence. The revised text (**page 9, lines 210-223**) is shown below:

"Despite extensive research, significant knowledge gaps remain regarding the long-term performance, operational robustness, and economic sustainability of physicochemical (PC) treatment systems under real-world industrial conditions. Most studies are limited to laboratory-scale experiments or short-term trials using synthetic or compositionally stable wastewaters **[7,10,14]**, which do not capture the temporal and compositional variability of actual industrial effluents **[1]**. For example, Bhagavath et al. **[26]** achieved satisfactory nickel removal with synthetic solutions but observed substantial performance declines when treating real effluents, underscoring the limitations of laboratory-based findings. At full scale, Gao et al. **[1]** reported persistent treatment instability in an eight-stage automated system, attributing recurrent non-compliance to improper dosing and insufficient adaptation to influent variability. This evidence shows that operational complexity alone does not ensure treatment robustness. Furthermore, process optimization has traditionally relied on univariate methods or statistical designs that assume constant influent characteristics, neither of which accurately reflects industrial conditions, and both of which limit system adaptability **[27]**."

Section 3.3 further substantiates these gaps through a detailed comparative analysis of the published literature, providing additional supporting evidence.

**Comment 7. They means? it is referring here what? chromic acidic effluent? right? will it be alkaline effluent or chromic alkaline effluent?**

**Response:**

We appreciate the reviewer’s precise and valid observations, which are addressed below.

— The pronoun "They" has been replaced with an explicit subject to eliminate ambiguity.

— We confirm that the correct term is "chromic acidic effluent" (E1).

— The alkaline effluents (E2) originate exclusively from degreasing and alkaline cleaning stages and do not contain chromium. They are correctly designated as "alkaline effluents" rather than "chromic alkaline effluents." This distinction has been clarified in the revised text. These revisions are indicated in the manuscript with track changes on **page11 (lines 259-265)**.

**Comment 8. Please mention the exact month with years when sampling was carried out**

**Response:**

Sampling campaigns were conducted from January 2, 2024, to March 20, 2025, comprising 59 consecutive weekly sampling events. This timeframe was chosen to capture the full range of temporal variability associated with production schedules, operational fluctuations, and seasonal effects.

The revision is documented in the updated manuscript, with track changes on **page 14 (lines 332-333)** marked in red.

**Comment 9. In Table 1, why concentration of HMs is not listed?**

**(For the other parameters concentration is given for E3 as well but why for HMs it is not given?**

**Response:**

We appreciate the reviewer’s observation. The concentrations of the key heavy metals, chromium (Cr) and nickel (Ni), have been added to the revised Table 1.

This revision is documented in the manuscript with track changes on **page 15**, and the changes are marked in red .

**Comment 10. Please mention values of parameters with standard deviation.**

**Why in the figures the values are below zero? also please add the standard deviation for the values in the Figures 1-3. what does this dash line indicates?**

**Response:**

We address each comment as follows:

**1. Standard Deviations:** In Table 1, certain parameters were initially reported as concentration ranges (minimum to maximum) rather than as mean ± SD. This approach was chosen because the dataset comprises 59 weekly grab samples that do not consistently follow a normal distribution. The use of ranges was intended to highlight worst-case compliance exceedances. Nonetheless, we acknowledge that inconsistent reporting of uncertainty is a valid concern.

We have now standardized the reporting of uncertainty throughout the manuscript as follows:

* For continuous monitoring data, mean ± SD values are now provided alongside the range. In some cases, values are reported as (minimum ± SD to maximum ± SD).

* For figures previously lacking error bars, all relevant figures have been updated to include error bars representing ± SD of triplicate measurements.

**2. Values Below Zero:** No values were below zero; the issue pertained to figure clarity. The figures have been revised to ensure that all data points are clearly and unambiguously displayed.

**3. Dashed Line:** The dashed line indicates the maximum permissible limit as defined by NT 106.002. To eliminate any potential ambiguity, this line has been removed from the revised Figures 1, 2, and 3.

All revisions are reflected in the updated manuscript, with track changes on **pages 15–18**.

**Comment 11. Why the pH was adjusted after coagulation process? what are the advantage of doing so rather than before coagulation process?**

**Response:**

The sequence of pH adjustments relative to coagulation was designed to meet the chemical requirements of each configuration:

**Configuration 1 (FeCl₃):** FeCl₃ is added first to destabilize suspended particles and promote metal complexation. A subsequent pH adjustment (neutralization) precipitates heavy metals as insoluble hydroxide. This sequence reflects the treatment protocol currently used at the industrial facility.

**Configuration 2 (PAC):** An initial pH adjustment is made before PAC coagulation to optimize hydrolysis speciation, which is highly pH-dependent. A subsequent pH adjustment is made after coagulation to complete metal hydroxide precipitation and achieve regulatory compliance.

**Configuration 3 (Lime):** Lime (Ca(OH)₂) serves as both a coagulant and a pH-adjusting agent, directly raising pH to the precipitation range (pH 6.5–9) and inducing the concurrent formation and aggregation of metal hydroxide flocs in a single step.

This clarification has been incorporated into **Section 2.3,** **pages 20–22**, and is marked in red.

**Comment 12. What were those parameters?**

**Response:**

The physicochemical (PC) parameters monitored in this study included pH, electrical conductivity (EC_w_, µS/cm), total suspended solids (TSS, mg/L), chemical oxygen demand (COD, mg/L), five-day biochemical oxygen demand (BOD₅, mg/L), chloride ions (Cl⁻, mg/L), and heavy metals, including chromium (Cr(III)), nickel (Ni), copper (Cu), zinc (Zn), iron (Fe), and lead (Pb).

This clarification appears in **Section 2.4** on **page 24**.

**Comment 13. Mention the model name of the instruments used.**

**Response:**

The revised manuscript now specifies the model names of the instruments used to measure pH and electrical conductivity on **page 15 (line 548-552)**.

**Comment 14. Can you please add a line justifying why only these parameters were considered for optimization? Also, why the Ni concentration was optimized? can you provide justification for that also.**

**Response:**

**Section 2.7.2** (**pages 31 and 32**) justifies the selection of the three independent variables ([Ni]_i_, pH, [A-PAM]). These parameters were identified from preliminary experimental results and are supported by relevant literature, as they represent the most influential factors governing nickel hydroxide precipitation, floc formation, and overall purification performance.

Including influent nickel concentration ([Ni]_i_) as an independent variable is a central methodological advancement. Full-scale monitoring showed substantial fluctuations in influent nickel (33–92 mg/L). Conventional response surface methodology (RSM) studies typically treat influent composition as fixed, yielding static optimal conditions that are ineffective in variable industrial settings. Incorporating [Ni]_i_ as a design variable allows the model to dynamically recalculate optimal pH and flocculant dosage in response to measured influent concentration, thereby transforming variability from an uncontrolled disturbance into an actionable process variable.

**Comment 15. Use correct notation: Cl-**

**There must be concentration of X in effluent. Please correct it**

**Response:**

The notation Cl⁻ has been corrected and applied consistently throughout the revised manuscript. The parameter for the 800–1100 mg/L concentration range is now explicitly specified, as indicated by track changes on **page 36 (lines 845-849)**.

**Comment 16. How can the same parameter be both input and output? Here it is nickel.**

**Response:**

We appreciate the reviewer’s observation and clarify that nickel is treated as both an input and an output variable in this study; however, these represent distinct measurements at different stages of the treatment process. The input variable denotes the initial influent nickel concentration ([Ni]ᵢ), measured before treatment and indicative of the raw wastewater composition. The output variable denotes the final residual nickel concentration ([Ni]f), measured in the treated effluent after the complete precipitation-flocculation process. In the sentence under discussion, nickel refers specifically to **[Ni]_f_**, the final residual concentration in the treated effluent, rather than the initial influent concentration.

The original sentence has been revised in the manuscript (**page 38, lines 905-907**). The updated text is as follows:

"Consistent with trends observed for chloride and chromium, process configuration 3 showed improved performance in reducing the final residual nickel concentration ([Ni]_f_), with effluent concentrations typically ranging from 1 to 5 mg/L."

**Why optimization was performed only for configuration 3?**

The three configurations were evaluated over a 24-week period. Results showed that Configuration 3, which used lime slurry-based precipitation, significantly outperformed Configurations 1 and 2. Configuration 3 achieved 99% chromium removal, 97% nickel removal, and 50% chloride reduction at a chemical operating cost of 0.134 €/m³, compared with 0.432 €/m³ for Configuration 1.

Configurations 1 and 2 exhibited inherent structural limitations. Configuration 1 experienced persistent chloride accumulation, whereas Configuration 2 failed to meet regulatory requirements under variable influent conditions. These limitations precluded further meaningful optimization. Therefore, Configuration 3 was selected as the sole candidate for RSM-CCD optimization to develop an adaptive predictive model for real-time dosing under variable influent conditions.

See pages 38-40.

**Comment 17. Please properly indicate in figure that what does this dash line indicates?**

The dashed line indicates the maximum permissible limit per Tunisian Standard NT 106.002. For clarity, this line has been omitted from the revised **Figures 7-9**.

See **page 33 and 34** of the revised manuscript.

**Comment 18. Please add in table 5 that which term was significant and which was not.**

The revised **Table 5** now includes a "Significance" column that indicates whether each model term is statistically significant at the *p* < 0.05 level.

This information appears on **page 50** of the revised manuscript.

**19. while optimizing why other two parmeters were not considered as response?**

We appreciate the reviewer’s insightful question. Our optimization focused exclusively on residual nickel concentration (**[Ni]_f_**) as the response variable for the following scientifically substantiated reasons.

**1. Chromium:** Full-scale monitoring of Configuration 3 showed consistently high and stable chromium removal, achieving up to **99%** under all operating conditions. Because of this systematic, near-complete removal, chromium concentrations in the treated effluent showed minimal variability and consistently remained below the regulatory limit established by NT 106.002 during the monitoring period. Including chromium as a response variable in the response surface methodology (RSM) framework would therefore have provided no additional value for optimization.

**2. Chloride:** Chloride concentration in the treated effluent is not primarily influenced by coagulation-precipitation chemistry. In Configuration 3, the observed 50% reduction in chloride results from dilution and physical separation rather than from chemical precipitation mechanisms regulated by pH or flocculant dosage. As a result, the two operational variables included in the RSM design—pH and [A-PAM] dosage—do not directly affect chloride removal. Therefore, including chloride as a response variable is not statistically or scientifically justified.

**3. Nickel:** In contrast, residual nickel concentration showed significant variability (0.02–60 mg/L) under conventional treatment, was highly sensitive to both pH and flocculant dosage, and showed a strong dependence on influent loading. Consequently, nickel was the most critical and technically challenging parameter to control, which justified its selection as the sole response variable in the optimization framework.

**20. it would be better to write this below table. don’t merge it with caption. do it throughout (Table 8)**

We appreciate the reviewer’s suggestion. Accordingly, the abbreviation list has been removed from the caption and added as a standalone note below Table 8 in the revised manuscript.

This change is documented in the revised manuscript with track changes and marked in red on **page 68** to facilitate the reviewer's identification.

**21. conclusion is very lengthy. reduce it by focusing on important concluding points**

We appreciate the Reviewer’s suggestion. We have revised and condensed the conclusion to highlight the key findings and eliminate redundancy. The revised conclusion is as follows:

"Strategic physicochemical reconfiguration, combined with variability-responsive statistical optimization, delivers superior treatment performance, enhanced operational stability, and exceptional economic efficiency in electroplating wastewater treatment. Over 24 weeks of full-scale industrial operation, lime slurry-based Configuration 3 achieved optimal results, removing 99% of chromium and 97% of nickel and reducing chloride levels by approximately 50% compared with conventional FeCl₃-based treatment. This configuration operated at 0.134 €·m⁻³, representing a 69% reduction relative to Configuration 1 (0.432 €·m⁻³). The principal scientific contribution is a variability-responsive RSM-CCD model ([Ni]_f_ = f([Ni]_i_, pH, [A-PAM], *R*² = 0.9917) that explicitly incorporates fluctuations in influent nickel concentration (33–92 mg/L) as an independent design factor. This approach, implemented for the first time in electroplating wastewater treatment, generates adaptive optimal dosing targets (pH 8.5–9.0, [A-PAM] 6–7 mg/L) that dynamically respond to influent variability, ensuring residual nickel remains below 2 mg/L throughout the operational domain. The quadratic pH effect (B² = +3.98, *p* < 0.0001) was experimentally validated and thermodynamically linked to the Ni(OH)₂ solubility minimum, confirming the physical significance of the model coefficients. These findings demonstrate that substantial performance improvements are achievable within established physicochemical treatment frameworks through intelligent process chemistry and adaptive statistical control, without reliance on capital-intensive emerging technologies."

We have incorporated this revision into the updated manuscript, with track changes visible on **page 72** and marked in red.

**Responses to Reviewer 2**

We thank Reviewer 2 for their thorough and constructive evaluation. Their insightful comments have enhanced the manuscript's mechanistic depth and environmental scope. All revisions addressing Reviewer 2's feedback are marked in red in the revised manuscript.

**Comment 1. What are the discharge limits for the concerned pollutants and mention standards in the introduction?**

**Response:**

We acknowledge that explicitly listing discharge limits in the Introduction is crucial for contextualizing the treatment challenge. Although the manuscript references the Tunisian NT 106.002 standard (INNORPI), the specific numerical limits were previously provided only in **Table 1** (Section 2.2). Accordingly, we have revised the Introduction to explicitly include the discharge thresholds for the most critical pollutants examined in this study. The following sentence has been added and is marked in red on **page 8 (line 190 -194)** of the revised manuscript:

" Specifically, NT 106.002 establishes the following limits for the primary pollutants addressed in this study: for discharge into the Public Hydraulic Domain (PHD), the maximum allowable concentrations are 0.5 mg/L for Cr(III), 0.2 mg/L for Ni(II), and 600 mg/L for Cl⁻. For discharge into the public sewerage network (ONAS), the respective limits are 2 mg/L, 1 mg/L, and 700 mg/L."

**Comment 2: What operating conditions critically influence the precipitation process?**

**Response:**

Preliminary experimental investigations and results from the response surface methodology-central composite design (RSM-CCD) model identified the most critical parameters controlling precipitation efficiency as follows:

**pH (dominant factor, F = 292.64, p < 0.0001):** Ni(OH)₂ precipitation is thermodynamically favored at pH 8.0-9.0. Below pH 7, precipitation remains incomplete, while above pH 10, hydroxide resolubilization may occur.

**Lime slurry dosage:** This parameter regulates the supply of alkalinity and helps maintain pH stability. A pronounced buffering transition zone between pH 6 and 9 is observed at lime dosages of 240-400 mg/L.

**Initial metal concentration ([Ni]_i_):** This factor affects both the stoichiometric lime demand and the kinetics of floc formation. The interaction between factors A and B (F = 5.70, p = 0.044) indicates that optimal pH set points shift as metal loading increases.

**Flocculant dosage ([A-PAM]):** The optimal dosage range is 6 to 8 mg/L. Dosing above 10 mg/L results in charge reversal and subsequent floc destabilization.

**Mixing intensity:** Rapid mixing at 64 rpm for 5 minutes ensures effective reagent dispersion, while slow mixing at 41 rpm for 15 minutes promotes floc growth. These parameters were held constant to simulate industrial process conditions.

**Temperature:** All experiments were conducted at 20-25°C. Lower temperatures reduce both precipitation kinetics and floc settling rates.

**Comment 3: State some of the previous RSM related studies for industrial effluents, their major outcomes and then correlate with the research gaps.**

**Response:**

Numerous studies have used response surface methodology (RSM) for industrial wastewater treatment, each with limitations that the present work directly addresses:

**Bhagavath et al. (2021)** achieved greater than 90% nickel removal from synthetic electroplating wastewater (EPW) using RSM. However, they observed significant performance degradation when treating real effluents, confirming that fixed-composition experimental designs tend to overestimate industrial performance.

**Gao et al. (2023)** operated a full-scale EPW treatment system but reported persistent regulatory noncompliance due to improper static dosing under variable influent conditions. This represents the specific gap addressed by the present study.

A critical gap in previous research is the treatment of influent metal concentration as a fixed parameter. This study is the first to explicitly incorporate influent nickel concentration ([Ni]iyieldingg/L) as a central composite design (CCD) factor, resulting in a model that is inherently adaptive to fluctuations in influent composition.

A dedicated section discussing prior RSM studies and the explicit research gap has been incorporated into the revised Introduction (**page 9, lines 210-223**).

"Despite extensive research, significant knowledge gaps remain regarding the long-term performance, operational robustness, and economic sustainability of physicochemical (PC) treatment systems under real-world industrial conditions. Most studies are limited to laboratory-scale experiments or short-term trials using synthetic or compositionally stable wastewaters **[7,10,14]**, which do not capture the temporal and compositional variability of actual industrial effluents **[1]**. For example, Bhagavath et al. **[26]** achieved satisfactory nickel removal with synthetic solutions but observed substantial performance declines when treating real effluents, underscoring the limitations of laboratory-based findings. At full scale, Gao et al. **[1]** reported persistent treatment instability in an eight-stage automated system, attributing recurrent non-compliance to improper dosing and insufficient adaptation to influent variability. This evidence shows that operational complexity alone does not ensure treatment robustness. Furthermore, process optimization has traditionally relied on univariate methods or statistical designs that assume constant influent characteristics, neither of which accurately reflects industrial conditions, and both of which limit system adaptability **[27]**."

**Comment 4. The manuscript consists of many short paragraphs. Authors are requested to properly format and improve the scientific language of manuscript.**

**Response:**

We thank the reviewer for this valuable comment. The manuscript has been carefully revised by the external consultant of the project who is a non-Arabic speaker, to improve the overall quality, clarity, and readability of the English language. The text was thoroughly proofread, and grammatical, stylistic, and typographical corrections were implemented throughout the manuscript.

**Comment 5. TSS removal exhibits extreme variability (2-118 mg/L in f-EPW vs. 2 ± 0.3 mg/L in E3), why there is standard deviation missing at some places**

**Response:**

We appreciate the reviewer’s observation. The pronounced variability in TSS concentrations in f-EPW (2 to 118 mg/L), compared with the consistently low TSS in the E3 influent (2 ± 0.3 mg/L), indicates operational inconsistency in settling and clarification under fluctuating sludge-loading conditions throughout the one-year monitoring period. The E3 inlet operates as a mechanically mixed tank with controlled bleed-down, resulting in minimal variance. In contrast, f-EPW is collected after open gravitational clarifiers, which are more susceptible to hydraulic surges, variations in sludge blanket height, and seasonal temperature fluctuations.

Regarding missing standard deviations, we note that this issue overlaps with the concern raised in Comment 10 by Reviewer 1; thus, we provide a unified response. In Table 1, certain parameters were initially reported as concentration ranges (minimum to maximum) rather than as mean ± SD because the dataset comprises **59 weekly grab** samples that do not consistently follow a normal distribution. The use of ranges was intentional to highlight worst-case compliance exceedances. Nonetheless, we acknowledge that inconsistent reporting of uncertainty is a valid concern.

We have now standardized the reporting of uncertainty throughout the manuscript as follows:

* For continuous monitoring data, mean ± SD values are now provided alongside the range, or, where appropriate, values are reported as (minimum ± SD to maximum ± SD).

* For figures previously lacking error bars, all relevant figures have been updated to include error bars representing ± SD of triplicate measurements.

**Comment 6. Authors are requested to discuss the mechanism in detail for metal speciation and precipitation chemistry in the manuscript**

**Response:**

We appreciate the Reviewer’s insightful comment. In response, we have provided a detailed discussion of the mechanisms underlying metal speciation and precipitation chemistry. **Section 2.1** has been revised on **pages 11-13** of the manuscript. This section now addresses the thermodynamics of Ni(OH)₂ and Cr(OH)₃ precipitation, pH-dependent speciation, and interactions among co-precipitating metals.

**Comment 7. Electroplating wastewater contains strong chelating agents such as EDTA, surfactants. How the mentioned coagulants break the metal-organic complexes and their kinetics effect the precipitation process?**

**Response:**

Electroplating baths typically contain EDTA, gluconates, and various surfactants, which form thermodynamically stable metal-organic complexes that resist conventional hydroxide precipitation.

The principal mechanisms by which coagulants interact with these complexes are as follows:

**Lime (pH > 8.5):** Calcium ions (Ca²⁺) competitively bind to EDTA (Ca-EDTA, log K = 10.6), partially displacing metal ions and facilitating their precipitation as hydroxides. Elevated Ca²⁺ concentrations promote co-precipitation of metal-organic complexes via charge neutralization within CaCO₃ or Ca(OH)₂ colloids.

**FeCl₃ and polyaluminum chloride (PAC):** Ferric ions (Fe³⁺) preferentially bind to EDTA (Fe-EDTA⁻, log K = 25.1), displacing Ni²⁺ and Cr³⁺ from their complexes. However, this process forms soluble Fe-organic complexes, which may increase the chemical oxygen demand (COD) in the effluent.

**Kinetic effects:** Competitive displacement of metal ions from organic complexes proceeds slowly at ambient temperature, accounting for the temporal instability observed in Configurations 1 and 2 under high organic-complex loading.

A dedicated interpretive paragraph has been added to **Section 3.1.1** (**pages 34-35, lines 815-827**) of the revised manuscript.

"Before interpreting the experimental results, it is necessary to consider the chemical speciation of metals in the E3 effluent matrix. This matrix is significantly more complex than a simple metal-hydroxide system because it contains strong chelating agents commonly found in electroplating bath formulations, including ethylenediaminetetraacetic acid (EDTA), gluconates, citrates, and various surfactants. These agents form thermodynamically stable organometallic complexes with nickel and chromium. A substantial portion of these metals remains in solution and is not readily available for hydroxide precipitation, which accounts for discrepancies between theoretical and experimentally observed residual metal concentrations. The coagulants used in this study influence the dissociation of these complexes in distinct ways. LS acts through both competitive calcium-ion binding to EDTA and sustained alkalinity supply, thereby shifting equilibria toward precipitation. In contrast, ferric chloride releases chelated metals via the competitive affinity of Fe³⁺ for EDTA. Still, this process results in the formation of soluble iron-EDTA complexes that are resistant to further precipitation **[40]**."

**Comment 8: The coefficients from the model should be more related to real chemistry**

**Response:**

We agree that the RSM coefficients warrant chemical interpretation rather than being treated solely as empirical numbers. Accordingly, we have revised **Section 3.2.1** (**pages 46-47, lines 1134-1156**) to provide chemical interpretations for each significant coefficient.

**Revised:**

"Coefficient analysis clarifies how factors influence nickel removal. The large negative pH coefficient (-4.20 in coded form) shows that increasing pH within the experimental range substantially reduces [Ni]_f_, consistent with the pronounced decrease in Ni(OH)₂ solubility from pH 7 to 9. Thermodynamically, each unit increase in pH decreases the equilibrium Ni²⁺ concentration by about two orders of magnitude, a trend qualitatively captured by this coefficient. The positive quadratic pH coefficient (3.98**) produces a response surface minimum and reflects the established re-dissolution of Ni(OH)₂ at very high pH, attributed to the formation of soluble [Ni(OH)₃]⁻ and [Ni(OH)₄]²⁻ hydroxo-complexes [32].** **The inflection point near pH 9–10,** consistent with **published solubility diagrams**, indicates an optimal pH beyond which further increases in pH may reduce removal efficiency, likely due to hydroxide resolubilization under highly alkaline conditions or the formation of soluble hydroxo complexes **[32]**. The negative AB interaction coefficient (-1.34) suggests that the beneficial effect of pH on nickel removal diminishes at higher [Ni]ᵢ, in agreement with precipitation kinetics. At elevated [Ni²⁺], the molar stoichiometric demand for OH⁻ increases, necessitating higher pH to maintain the same driving force for nucleation and growth of Ni(OH)₂ crystals, rather than merely reflecting saturation of precipitation capacity or kinetic limitations under increased metal loading. The positive linear [Ni]ᵢ coefficient (+1.07) indicates that higher influent concentrations yield higher residual concentrations, consistent with mass-balance constraints near the precipitation capacity. The positive BC interaction coefficient (+ 0.735) **indicates that A-PAM flocculation is more effective when Ni(OH)₂ precipitates are fully formed (pH ≥ 8.5), as the anionic polymer bridges positively surface-charged hydroxide colloids. At low pH, incomplete precipitation produces fewer bridgeable particles, thereby limiting the flocculant' s contribution to nickel removal.**"

**Comment 9: Why are emerging contaminants discussed only in the introduction but not in results and discussion?**

**Response:**

The observation is valid. Emerging contaminants (EMCs), specifically N-nitrosamines referenced in the Introduction, were not included in the analytical monitoring program for the following reasons: (i) the primary focus was on critical regulatory non-compliance parameters (Ni, Cr, Cl⁻); (ii) validated N-nitrosamine analysis requires specialized instrumentation unavailable at the industrial monitoring laboratory; and (iii) N-nitrosamine precursors, such as secondary amines from surfactants, were expected to be partially removed during the precipitation process, along with COD.

The revised Introduction **(page 7, lines 165-171)** now reads:

" Although a full study would examine all types of pollutants in this wastewater, this research primarily focuses on removing heavy metals first, because regulators monitor them most closely and they cause most of the toxic effects. The main reason is that removing heavy metals directly addresses the biggest environmental issues. It is expected that some of the new contaminant sources will also be partially removed along with organic matter during chemical treatment, as indicated by lower chemical oxygen demand (COD) **[5,6]**.

**Comment 10: The PC parameters used to evaluate the performance of the EPW investigated in this study were systematically analyzed using standardized and validated analytical methods. Mention the references or cite it**

**Response:**

Thank you for your observation. In response, the revised manuscript (**Section 2.4**) now explicitly cites the following references for each analytical method (**pages 23-25**).

" pH was measured with a calibrated digital pH meter (AZ Instrument, model 86501) following APHA Method 4500-H⁺ B [APHA, 2017]. Electrical conductivity (ECw, µS/cm), an indicator of wastewater ionic strength and total dissolved salts, was determined with a benchtop conductivity meter (Eutech Instruments, model CON2700) following APHA Method 2510B [APHA, 2017].

Total suspended solids (TSS) were quantified using the standard gravimetric method in accordance with APHA guidelines (APHA, 2017) **[34]**. Samples were filtered through pre-weighed membrane filters, then dried at 105°C and reweighed to determine suspended particulate concentrations.

Chemical oxygen demand (COD) was measured using the dichromate reflux method (ISO 15705) **[35]**, which quantifies the total organic matter oxidizable by dichromate in wastewater. Biochemical oxygen demand over five days (BOD₅) was determined by the standard dilution and incubation procedure (APHA, 2017) **[34]**, providing an estimate of the biodegradable organic load.

Chloride ions (Cl⁻, mg/L) were analyzed by argentometric titration using Mohr's method (APHA et al., 2017) **[34]**. Heavy metals (HMs), including chromium (Cr(III)), nickel (Ni), copper (Cu), zinc (Zn), iron (Fe), and lead (Pb), were quantified by inductively coupled plasma atomic emission spectroscopy (ICP-AES, Activa-HORIBA JOBIN YVON).

This analytical technique enabled accurate determination of trace metal concentrations, which serve as critical indicators for assessing EPW contamination and treatment efficiency."

**Comment 11: The manuscript report ~50% chloride removal which is unlikely as chloride mostly remain soluble. Explain it and perform the mass balance.**

**Response:**

We thank the reviewer for this important observation and provide the following clarification. The observed ~50% reduction in chloride concentration in Configuration 3 does not result from chloride precipitation but from the following factors:

**• Elimination of chloride input from FeCl₃:** Configuration 1 uses FeCl₃ as a coagulant at 0.864 kg/m³. Each mole of FeCl₃ releases three moles of Cl⁻ upon dissolution, contributing approximately 530 mg Cl⁻/L to the treated effluent. In Configuration 3, FeCl₃ is replaced with lime (Ca(OH)₂), which is chloride-free, thereby removing this significant secondary chloride source. This change accounts for most of the observed 50% reduction in effluent chloride levels relative to Configuration 1 (800–1500 mg/L Cl⁻).

**• Physical enmeshment and co-sedimentation:** Minor but quantifiable amounts of chloride may become physically entrained within sludge flocs and subsequently removed during settling. This represents a small, non-chemical contribution.

**• Dilution effects:** Minor dilutions resulting from pH adjustments between configurations may also contribute to a small extent.

Simplified mass balance comparison between Configuration 1 and Configuration 3:

| **Source** | **Configuration 1 (mg/L)** | **Configuration 3 (mg/L)** |
| --- | --- | --- |
| Cl⁻ from E3 influent (mean) | ~1150 | ~1150 |
| Cl⁻ added by FeCl₃ coagulant | +530 | 0 (lime used) |
| Expected effluent Cl⁻ | ~1680 | ~1150 |
| Observed effluent Cl⁻ (mean) | ~1150 | ~650 |

In response to this comment, the interpretation of chloride behavior across the three configurations (**Section 3.1.1**, **pages 35 and 36, lines 840-866**) has been comprehensively revised and replaced with the following corrected interpretation, marked in red in the revised manuscript:

"Chloride management varied considerably among the three configurations, underscoring the direct impact of coagulant selection on effluent chloride loading. In Configuration 1, effluent chloride concentrations ranged from 800 to 1500 mg/L and showed high temporal variability throughout the monitoring period, consistently exceeding the NT 106.002 discharge limits. This suboptimal performance is primarily attributed to the use of FeCl₃ as a coagulant, which, upon dissolution and hydrolysis, releases additional Cl⁻ into the treated effluent, thereby increasing the chloride load rather than mitigating it **[41,42]**.

Configuration 2 showed a marked improvement over Configuration 1, with effluent chloride concentrations stabilizing between 800 and 1100 mg/L and exhibiting greater temporal consistency. Replacing FeCl₃ with polyaluminum chloride (PAC) eliminated the secondary chloride input associated with iron-based coagulation, thereby reducing the overall effluent chloride load. Although PAC contains chloride in its chemical structure, its contribution per unit dose is significantly lower than that of FeCl₃, which explains the partial improvement observed **[43]**. However, actual Cl⁻ removal through incorporation into precipitated solids remains negligible, as chloride ions do not form insoluble hydroxide or carbonate phases under the prevailing treatment conditions.

The most substantial improvement occurred with Configuration 3, where effluent chloride concentrations progressively decreased and stabilized at 550–750 mg/L during the final monitoring phase, particularly in weeks 17–24. This represents an approximate 50% reduction compared to the effluent levels of Configuration 1. However, this reduction does not indicate true Cl⁻ precipitation or removal from the aqueous phase, as chloride ions remain soluble under all physicochemical treatment conditions and are not subject to hydroxide co- precipitation **[41]**. Instead, the observed reduction is primarily due to the complete elimination of FeCl₃ as a secondary chloride source. At the coagulant dosage used in Configuration 1 (0. 864 kg/m ³), FeCl₃ hydrolysis releases an estimated 530 mg Cl⁻/L into the treated effluent. This substantial secondary chloride load is entirely avoided in Configuration 3 by substituting FeCl₃ with chloride- free lime slurry (Ca(OH)₂)."

**Comment 12: Unit and notations should be consistent**

**Response:**

We appreciate the reviewer’s valuable observation regarding quality. We have systematically reviewed all units and notations throughout the manuscript. The following inconsistencies have been addressed:

**• Concentration units:** The terms 'ppm' and 'mg/L' were previously used inconsistently. Because all solutions are aqueous and dilute, with a density of approximately 1 g/mL, ppm ≈ mg/L is valid. However, for scientific consistency, all concentrations are now reported exclusively as 'mg/L'.

**• Operating cost:** The notations '€/m³' and '€·m⁻³' have been standardized to '€·m⁻³' throughout the manuscript in accordance with SI conventions.

**• Ionic notation:** The notations 'Cl-', 'Ni2+', and 'Cr3+' have been corrected to 'Cl⁻', 'Ni²⁺', and 'Cr³⁺', respectively, in all instances throughout the text. Abbreviation inconsistency: 'TSS' and 'TSSs' have been unified to 'TSS'; 'A-PAM' and 'PAM', used synonymously, have been standardized to 'A-PAM' throughout.

• Figure axis labels and table column headers have been reviewed to ensure that unit symbols are consistently enclosed in parentheses (unit).

**Comment 13: Some figures don’t have error bars**

**Response:**

We appreciate the reviewer’s insightful observation. Accordingly, error bars have been added to all relevant figures in the revised manuscript.

**Comment 14: Discuss about sludge management, recyclability and how it effect the economic aspect**

**Response:**

We appreciate the reviewer’s valuable suggestion. While the original manuscript briefly addressed the advantages of lime treatment in the economic section, we have now added a new section titled "**4.****Environmental implications and research perspectives**" This section provides a comprehensive discussion of sludge management and recyclability, and their influence on the economic aspects of the treatment process. The addition is included in the revised manuscript with track changes on **pages 68-70** and is marked in red for clarity.

**Comment 15: Can authors explain the superiority of lime treatment over current research trends such as adsorption, electrocoagulation, and membrane processes**

**Response:**

We appreciate the reviewer’s insightful question. The manuscript already includes a comparative discussion in **Section 3.3** and **Table 8** (**pages 62-68**), which systematically situates the present study relative to conventional and emerging EPW treatment technologies. The following consolidated response is drawn directly from that section.

**Comparative Advantages of Lime-Based Treatment over Alternative Technologies**

**Comparison with Adsorption Technologies (Ref. [7]: UBSB biochar; Ref. [10]:** subcritical hydrothermal plus adsorption): Adsorption-based methods reported in the literature demonstrate high removal efficiencies. For example, Ref. [7] achieved up to 100% chromium removal and 90–100% copper removal using biochar derived from real electroplating wastewater (EPW), while Ref. [10] reported 99.97% residual nickel removal. However, these outcomes were obtained exclusively at the laboratory scale under controlled conditions using synthetic or highly diluted effluents. Notably, neither study has been scaled up to an industrial application. In contrast, Configuration 3 was validated over 24 continuous weeks at full industrial scale, treating authentic post-dechromatation wastewater containing variable nickel concentrations (33–92 mg/L), residual chromium, competing metals, organic complexants, and elevated chloride levels. These factors are known to systematically reduce adsorption performance due to competitive binding and rapid exhaustion of adsorption capacity. Bhagavath et al. [26] further observed that nickel removal efficiency from synthetic solutions (90–91%) declined substantially when treating real effluents with nickel concentrations exceeding 1000 mg/L, necessitating dilution. This requirement represents a fundamental limitation of adsorption technologies, whereas lime precipitation, operating stoichiometrically and without capacity constraints, does not exhibit it.

**Compared with electrocoagulation (Ref. [12]: PE-SACC; Ref. [14]: EO)**, Tang et al. [12] achieved high removal efficiencies (Cu: 99.3%, Zn: 99.1%, Ni: 98.4%) with photovoltaic-powered sinusoidal AC electrocoagulation, at an energy consumption of 0.105 kWh/m³ and an operating cost of approximately 0.019 €/m³. These costs are substantially lower than those of Configuration 1 (0.432 €/m³) and Configuration 2 (0.303 €/m³). However, these findings are based on laboratory or pilot-scale experiments with synthetic single-metal solutions. The technology also requires significant capital investment, frequent electrode cleaning (every 7 days), and the addition of conductive salts for low-conductivity matrices. Similarly, Ref. [14] reports 97.6% Cr(VI) and 87.3% COD removal using a novel Ti/TiO₂-GO-SnO₂ electrode, but at a high energy cost of 14.97 kWh/m³ (approximately 1.12 €/m³), which limits economic feasibility at full scale. In contrast, Configuration 3 achieves a chemical cost of 0.134 €/m³, representing a 69% reduction compared to conventional FeCl₃-based treatment (Configuration 1). This is accomplished without energy-intensive electrochemical processes, instead using gravity settling and optimized reagent chemistry, providing a significant economic advantage for industrial applications.

**Comparison with membrane processes (Ref. [25] — AGMD + SLM + ED hybrid):** The membrane hybrid system described in Ref. [25], which integrates air gap membrane distillation, supported liquid membrane, and electrodialysis, achieves high nickel recovery rates (AGMD: 99.4%, SLM: 76%, ED: 81%) from real EPW. However, this multi-stage process requires sequential operations, extended treatment times (240 minutes), and complex protocols, and it has not been demonstrated beyond the laboratory scale. Membrane processes are also highly susceptible to fouling under the elevated total suspended solids and organic loading typical of real EPW, and are economically feasible only for high-value metal recovery. Consequently, these processes are best suited as polishing or recovery steps following a primary chemical treatment stage, such as Configuration 3.

**Comparison with multi-stage physicochemical systems (Ref. [1]):** Gao et al. [1] operated a sophisticated, eight-stage, full-scale system with automated controls, yet the facility exhibited persistent treatment instability. Nickel removal fluctuated between 40% and 90%, and treated wastewater frequently failed to meet discharge standards due to improper dosing and unreliable monitoring. The total operating cost exceeded 7.80 €/m³, nearly 58 times higher than that of Configuration 3 (0.134 €/m³). This elevated cost was primarily due to multiple chemical additions across eight stages and sludge management expenses, which alone accounted for 4.97 €/m³. These findings indicate that increased system complexity does not ensure treatment stability, whereas Configuration 3 achieves superior performance through optimized chemistry rather than process multiplication.

**Summary of lime treatment advantages:**

A comparative analysis shows that Configuration 3's lime-based chemical precipitation offers several key advantages: (i) demonstrated full-scale industrial reliability over 24 weeks under variable EPW conditions; (ii) multifunctional reagent action that combines pH control, metal precipitation, and coagulation in a single dosing step; (iii) the lowest operating cost among all evaluated technologies (0.134 €/m³); (iv) no energy-intensive process drivers; (v) superior sludge dewaterability relative to coagulant-polymer systems [26]; and (vi) robust performance under variable influent metal loading (Ni: 33–92 mg/L), as validated by the RSM-CCD model (*R*² = 0.9917). Although electrocoagulation and membrane technologies offer specific benefits in controlled laboratory environments, lime-based chemical precipitation remains the most industrially mature, economically competitive, and operationally resilient primary treatment option for high-strength, variable EPW. Advanced technologies are best integrated as downstream polishing stages.

**Comment 16: The manuscript reports a significant quadratic effect of pH on nickel removal. Experimentally validate it by evaluating residual Ni over pH range to identify minimum solubility region, re-dissolution at higher pH**

**Comment 17: Correlate RSM optimum pH with minimum thermodynamic solubility**

**Response:**

We appreciate the reviewer’s complimentary and constructive comments on both the experimental validation of the quadratic pH effect and its thermodynamic interpretation. Because these comments are closely related, with Comment 16 requesting experimental evidence of pH-dependent solubility and Comment 17 seeking its correlation with the thermodynamic minimum solubility of Ni(OH)₂, we provide a unified response addressing both points.

In response, we designed and conducted a laboratory-scale validation experiment using authentic industrial E3 effluent. The optimum pH predicted by response surface methodology (RSM) aligned with the theoretical minimum solubility of Ni(OH), as determined by thermodynamic equilibrium calculations. The complete experimental protocol, results, and thermodynamic correlation are now presented in a **new subsection 3.2.5,** titled "**Experimental verification of the quadratic pH effect and its thermodynamic correlation**" on **pages 59-62** of the revised manuscript. All additions are marked in red.

**Comment 18: Surface interaction studies can be performed to analyse the role of adsorption along with precipitation. Isotherm model can be fitted.**

**Response:**

This suggestion is scientifically relevant. In lime-based treatment, Ni(II) removal occurs via two parallel mechanisms: (i) homogeneous precipitation of Ni(OH)₂ from solution when the ion product exceeds the solubility product (*K*_sp_); and (ii) surface adsorption or co-precipitation of Ni²⁺ onto the surfaces of newly formed Cr(OH)₃ flocs and Ca(OH)₂ particles, which provide high-surface-area, negatively charged surfaces at pH values greater than 8.

Precipitation remains the dominant removal mechanism; however, adsorption becomes more significant at low initial Ni concentrations (≤45 mg/L), where the ion product approaches the solubility product and surface adsorption provides additional removal capacity. Comprehensive isotherm studies, complemented by surface characterization techniques such as BET, SEM-EDX, and FTIR, are warranted in future work, as noted on **page 69**.

**Comment 19: Discuss the role of mixing speed during jar test, how it effects the diffusion?**

**Response:**

Mixing speed determines the hydrodynamic conditions for particle collisions and mass transfer, which directly govern the kinetics of precipitation and flocculation.

**During the rapid mixing phase (64 rpm, 5 min),** the lime slurry is fully dispersed, preventing local pH hot spots. This phase promotes diffusion-limited neutralization reactions and initiates Ni(OH)₂ nucleation.

**In the slow mixing phase (41 rpm, 15 min)**, orthokinetic flocculation facilitates the aggregation of primary Ni(OH)₂ and Cr(OH)₃ microflocs under controlled fluid shear. Shear rates above 100 rpm cause floc breakup and reduce settling efficiency.

These parameters were selected to align with the industrial facility's operating protocol, ensuring that response surface methodology (RSM) model predictions are directly applicable to full-scale implementation without scale-up corrections. A dedicated discussion of this alignment appears on **pages 27–28 (lines 650-667)**.

**Comment 20: Environmental and health implication can be discussed in separate section**

**Response:**

A new section, "**4. Environmental implications and research perspectives**" (**pages 68–70**, marked in red ), has been added. This section consolidates environmental and health implications, sludge management and valorization (Comment 14), and future research priorities within a unified environmental assessment framework.

This section addresses eliminating acute ecotoxicological risk from nickel and chromium discharges, reducing the secondary chloride load to enable potential water reuse, and sludge valorization pathways, such as nickel recovery via electrowinning and chromium(III) oxide production via calcination. It also outlines recommended future investigations, including multi-response response surface methodology, life-cycle cost analysis, surface characterization, assessment of emerging contaminant fate, and evaluation of transferability to other industrial effluents.

We believe the revisions address all comments and improve the manuscript's suitability for publication in PLOS ONE. We are available to provide any additional information or clarification as needed.

Sincerely,

The Authors
