# Supplementary material for: Integrated optimization of electroplating wastewater treatment: A comparative assessment of physicochemical treatment configurations and process enhancement using response surface methodology
Source: PLoS One. 2026 Aug 21;21(8):e0345965. doi: 10.1371/journal.pone.0345965 (PMC13496402; doi:10.1371/journal.pone.0345965)
Supplement: Supplementary file 4 [file pone.0345965.s006.docx]

**Response to Reviewers**

Manuscript PONE-D-26-12528R1

*Title: Integrated optimization of electroplating wastewater treatment: A comparative assessment of physicochemical treatment configurations and process enhancement using response surface methodology*

Journal: PLOS ONE

Dear Dr. Sovik Das and Reviewers,

We thank the Editor and Reviewers for the careful re-evaluation of our manuscript and for confirming that all previous comments have been adequately addressed. We are grateful for the opportunity to respond to the remaining point raised in this round of review. Our point-by-point response is provided below, with reviewer comments reproduced in italics and our responses given underneath. Changes made to the manuscript are indicated where relevant.

**Journal Requirements (Academic Editor)**

**Comment A:** *“If the reviewer comments include a recommendation to cite specific previously published works, please review and evaluate these publications to determine whether they are relevant and should be cited.”*

**Response:** We thank the Editor for this note. Reviewer’s comment did not recommend any specific previously published work to be cited; the comment concerned the methodology used for chemical speciation (addressed in detail under Reviewer #2, Comment 3 below). No additional citation was therefore required in response to this point.

**Comment B:** *“Please review your reference list to ensure that it is complete and correct. If you have cited papers that have been retracted, please include the rationale for doing so in the manuscript text, or remove these references and replace them with relevant current references. Any changes to the reference list should be mentioned in the rebuttal letter that accompanies your revised manuscript. If you need to cite a retracted article, indicate the article’s retracted status in the References list and also include a citation and full reference for the retraction notice.”*

**Response:** We confirm that the full reference list has been carefully reviewed for this revision. All references have been checked for completeness and accuracy (authors, titles, journal names, years, volumes, and DOIs where available), and none of the cited works have been identified as retracted. No changes were made to the reference list in this revision round.

**Reviewer #2**

**Comment 1:** *“All comments have been addressed.”*

**Response:** We thank the reviewer for this positive assessment and for confirming that all comments raised in the previous round of review have been satisfactorily addressed.

**Comment 2:** *Is the manuscript technically sound, and do the data support the conclusions? — “Yes.”*

**Response:** We thank the reviewer for this favorable evaluation confirming that the manuscript is technically sound and that the data support the conclusions drawn.

**Comment 3:** *Has the statistical analysis been performed appropriately and rigorously? — “No” (no specific methodological concern was described in the accompanying comments).*

**Response:** We thank the reviewer for this evaluation and would welcome any further clarification, as the written comments provided relate exclusively to the chemical speciation point addressed below (Comment 6), with no additional statistical concern specified. We would like to take this opportunity to reaffirm the statistical rigor underlying our RSM model, as detailed in Section 3.2.2 (“Statistical Analysis and Model Validation”) of the manuscript. The experimental design and statistical analysis were performed using Minitab software based on a central composite design (CCD). Model significance was assessed by analysis of variance (ANOVA), yielding a highly significant model F-value of 111.37 (p < 0.0001), together with significant individual and interaction terms (pH, [Ni]i × pH, pH × flocculant dosage, and the quadratic pH term) consistent with the underlying precipitation chemistry (Table 5). Model adequacy was further confirmed through multiple validation metrics (Table 6): a correlation coefficient R² = 0.9917, an adjusted R² of 0.9832, and a predicted R² of 0.9352, whose close mutual agreement (difference < 0.2) rules out overfitting. The adequate precision ratio of 37.08, well above the minimum acceptable threshold of 4, further confirms an excellent signal-to-noise ratio. We trust that these results reaffirm the statistical soundness of the analysis, and we remain fully available to expand on any specific aspect the reviewer may wish to see clarified further in the manuscript.

**Comment 4:** *Have the authors made all data underlying the findings in their manuscript fully available? — “Yes.”*

**Response:** We thank the reviewer for confirming that the underlying data have been made fully available, in accordance with the PLOS Data Availability policy.

**Comment 5:** *Is the manuscript presented in an intelligible fashion and written in standard English? — “Yes.”*

**Response:** We thank the reviewer for confirming that the manuscript is clearly and correctly written in standard English.

**Comment 6:** *“How was the speciation performed? Suggestion is to use Visual Minteq OR appropriate reference for speciation.”*

**Response:** We thank the reviewer for this important observation. We would like to clarify that this point had already been addressed during the previous (major) revision round, in which we added a dedicated subsection (“Experimental Validation of the Quadratic pH Effect and Thermodynamic Correlation”) explicitly treating nickel speciation on the basis of an established thermodynamic reference rather than software-based modeling. Specifically, the residual soluble Ni fraction at each pH was interpreted using the classical Ni(II) hydrolysis/speciation framework of Stumm & Morgan (1996), which provides the solubility product of Ni(OH)₂ (Ksp = 5.5 × 10⁻¹⁶ at 25°C) together with the cumulative stability constants of the successive Ni(II) hydroxo-complexes (log β₁–β₄ = 4.97, 8.55, 11.33 and 13.60, respectively).

In that subsection, the theoretical soluble Ni(II) concentration was calculated as a function of pH from the full speciation expression:

*[Ni] = [Ni²⁺] × (1 + β₁[OH⁻] + β₂[OH⁻]² + β₃[OH⁻]³ + β₄[OH⁻]⁴), with [Ni²⁺] = Ksp / [OH⁻]²*

This theoretical solubility curve was directly compared with our experimental residual Ni concentrations measured across pH 6.0–12.0 on authentic industrial effluent (Fig. 12 of the manuscript). The comparison confirms that the RSM-derived quadratic pH effect (B² = +3.98, p < 0.0001) is thermodynamically consistent with Ni(OH)₂ speciation behavior, capturing (i) incomplete precipitation below pH 7.5, (ii) a minimum-solubility zone at pH 8.0–9.5 driven by Ni(OH)₂ precipitation together with co-precipitation onto Cr(OH)₃ flocs and A-PAM enmeshment, and (iii) re-dissolution above pH 9.5 due to the formation of soluble anionic hydroxo-complexes [Ni(OH)₃]⁻ and [Ni(OH)₄]²⁻.

It should be clarified that the residual Ni concentration was measured by ICP, a technique that quantifies the total elemental metal content but does not, by its nature, allow the different chemical forms present in solution to be distinguished. It is precisely for this reason that the interpretation of Ni speciation was performed a posteriori, using the thermodynamic framework of Stumm & Morgan (1996), by comparing the ICP-measured total Ni concentrations with the theoretical Ni(OH)₂ solubility curve calculated as a function of pH.

We believe that this thermodynamic speciation analysis, grounded in the well-established reference of Stumm & Morgan (1996) and validated against our own experimental data, satisfactorily addresses the reviewer’s request and reinforces the mechanistic robustness of the RSM model. Visual MINTEQ was not additionally used, as the analytical speciation framework already accounts for the full set of Ni(II) hydroxo-complexes relevant to our effluent matrix.

We thank the Academic Editor and Reviewer #2 once again for their constructive feedback, which has helped strengthen the scientific rigor of our manuscript. We hope that the revised version is now suitable for publication in PLOS ONE.

Sincerely,

**Dr. Soumaya Ibrahimi**

*On behalf of all co-authors*
