# Supplementary material for: Integrated optimization of electroplating wastewater treatment: A comparative assessment of physicochemical treatment configurations and process enhancement using response surface methodology
Source: PLoS One. 2026 Aug 21;21(8):e0345965. doi: 10.1371/journal.pone.0345965 (PMC13496402; doi:10.1371/journal.pone.0345965)
Supplement: Peer review history

# Peer review: Decision Letter 0

**DOI:** 10.1371/journal.pone.0345965.r001

**Authors:** Sovik Das

27 Apr 2026

Dear Dr. Ibrahimi,

Thank you for submitting your manuscript to PLOS ONE. After careful consideration, we feel that it has merit but does not fully meet PLOS ONE’s publication criteria as it currently stands. Therefore, we invite you to submit a revised version of the manuscript that addresses the points raised during the review process.

Please submit your revised manuscript by Jun 11 2026 11:59PM. If you will need more time than this to complete your revisions, please reply to this message or contact the journal office at plosone@plos.org. When you're ready to submit your revision, log on to https://www.editorialmanager.com/pone/ and select the 'Submissions Needing Revision' folder to locate your manuscript file.

- A letter that responds to each point raised by the academic editor and reviewer(s). You should upload this letter as a separate file labeled 'Response to Reviewers
- A marked-up copy of your manuscript that highlights changes made to the original version. You should upload this as a separate file labeled 'Revised Manuscript with Track Changes'.
- An unmarked version of your revised paper without tracked changes. You should upload this as a separate file labeled 'Manuscript'.

If you would like to make changes to your financial disclosure, please include your updated statement in your cover letter. Guidelines for resubmitting your figure files are available below the reviewer comments at the end of this letter.

If applicable, we recommend that you deposit your laboratory protocols in protocols.io to enhance the reproducibility of your results. Protocols.io assigns your protocol its own identifier (DOI) so that it can be cited independently in the future. For instructions see: https://journals.plos.org/plosone/s/submission-guidelines#loc-laboratory-protocols. Additionally, PLOS ONE offers an option for publishing peer-reviewed Lab Protocol articles, which describe protocols hosted on protocols.io. Read more information on sharing protocols at https://plos.org/protocols?utm_medium=editorial-email&utm_source=authorletters&utm_campaign=protocols.

As the corresponding author, your ORCID iD is verified in the submission system and will appear in the published article. PLOS supports the use of ORCID, and we encourage all coauthors to register for an ORCID iD and use it as well. Please encourage your coauthors to verify their ORCID iD within the submission system before final acceptance, as unverified ORCID iDs will not appear in the published article. *Only*  the individual author can complete the verification step; PLOS staff *cannot*  verify ORCID iDs on behalf of authors.

We look forward to receiving your revised manuscript.

Kind regards,

Sovik Das

Academic Editor

PLOS One

Journal Requirements:

When submitting your revision, we need you to address these additional requirements.

1. Please ensure that your manuscript meets PLOS ONE's style requirements, including those for file naming. The PLOS ONE style templates can be found at

https://journals.plos.org/plosone/s/file?id=wjVg/PLOSOne_formatting_sample_main_body.pdf and

https://journals.plos.org/plosone/s/file?id=ba62/PLOSOne_formatting_sample_title_authors_affiliations.pdf.

2. Thank you for stating in your Funding Statement:

When submitting your revision, we need you to address these additional requirements.

1. Please ensure that your manuscript meets PLOS ONE's style requirements, including those for file naming. The PLOS ONE style templates can be found at

https://journals.plos.org/plosone/s/file?id=wjVg/PLOSOne_formatting_sample_main_body.pdf and

https://journals.plos.org/plosone/s/file?id=ba62/PLOSOne_formatting_sample_title_authors_affiliations.pdf.

2. Please note that PLOS One has specific guidelines on code sharing for submissions in which author-generated code underpins the findings in the manuscript. In these cases, we expect all author-generated code to be made available without restrictions upon publication of the work. Please review our guidelines at https://journals.plos.org/plosone/s/materials-and-software-sharing#loc-sharing-code and ensure that your code is shared in a way that follows best practice and facilitates reproducibility and reuse.

3. Thank you for stating in your Funding Statement:

“This research was supported and funded by the Deanship of Scientific Research at Imam Mohammad Ibn Saud Islamic University (IMSIU) (grant number IMSIU-DDRSP2602). “

Please provide an amended statement that declares *all* the funding or sources of support (whether external or internal to your organization) received during this study, as detailed online in our guide for authors at http://journals.plos.org/plosone/s/submit-now.  Please also include the statement “There was no additional external funding received for this study.” in your updated Funding Statement.

Please include your amended Funding Statement within your cover letter. We will change the online submission form on your behalf.

4. We note that your Data Availability Statement is currently as follows: [All relevant data are within the manuscript and its Supporting Information files. ]

Please confirm at this time whether or not your submission contains all raw data required to replicate the results of your study. Authors must share the “minimal data set” for their submission. PLOS defines the minimal data set to consist of the data required to replicate all study findings reported in the article, as well as related metadata and methods (https://journals.plos.org/plosone/s/data-availability#loc-minimal-data-set-definition).

For example, authors should submit the following data:

- The values behind the means, standard deviations and other measures reported;

- The values used to build graphs;

- The points extracted from images for analysis.

Authors do not need to submit their entire data set if only a portion of the data was used in the reported study.

If your submission does not contain these data, please either upload them as Supporting Information files or deposit them to a stable, public repository and provide us with the relevant URLs, DOIs, or accession numbers. For a list of recommended repositories, please see https://journals.plos.org/plosone/s/recommended-repositories.

If there are ethical or legal restrictions on sharing a de-identified data set, please explain them in detail (e.g., data contain potentially sensitive information, data are owned by a third-party organization, etc.) and who has imposed them (e.g., an ethics committee). Please also provide contact information for a data access committee, ethics committee, or other institutional body to which data requests may be sent. If data are owned by a third party, please indicate how others may request data access.

5. Thank you for stating the following in the Acknowledgments Section of your manuscript:

“This research was supported and funded by the Deanship of Scientific Research at Imam Mohammad Ibn Saud Islamic University (IMSIU) (grant number IMSIU-DDRSP2602).”

We note that you have provided additional information within the Acknowledgements Section that is not currently declared in your Funding Statement. Please note that funding information should not appear in the Acknowledgments section or other areas of your manuscript. We will only publish funding information present in the Funding Statement section of the online submission form.

Please remove any funding-related text from the manuscript and let us know how you would like to update your Funding Statement. Currently, your Funding Statement reads as follows:

“This research was supported and funded by the Deanship of Scientific Research at Imam Mohammad Ibn Saud Islamic University (IMSIU) (grant number IMSIU-DDRSP2602).”

Please include your amended statements within your cover letter; we will change the online submission form on your behalf.

6. If the reviewer comments include a recommendation to cite specific previously published works, please review and evaluate these publications to determine whether they are relevant and should be cited. There is no requirement to cite these works unless the editor has indicated otherwise.

[Note: HTML markup is below. Please do not edit.]

Reviewers' comments:

Reviewer's Responses to Questions

**Comments to the Author**

1. Is the manuscript technically sound, and do the data support the conclusions?

Reviewer #1: Partly

Reviewer #2: Partly

**********

2. Has the statistical analysis been performed appropriately and rigorously?

Reviewer #1: Yes

Reviewer #2: Yes

**********

3. Have the authors made all data underlying the findings in their manuscript fully available?

The PLOS Data policy

Reviewer #1: No

Reviewer #2: Yes

**********

4. Is the manuscript presented in an intelligible fashion and written in standard English?

Reviewer #1: No

Reviewer #2: No

**********

Reviewer #1: This manuscript presents an attempt to optimize operating parameters for electroplating wastewater treatment using Response Surface Methodology (RSM). While the approach is relevant, this paper lacks a novelty statement. The technical depth of the work is also insufficient, with limited mechanistic insight and inadequate justification of the chosen parameters. The manuscript is somewhat confusing and lacks coherence, making it difficult to follow the overall narrative. The flow between sections is weak, and the study lacks a logically connected progression of ideas. Additionally, some of the underlying assumptions and basis of the study appear questionable and are not adequately supported by scientific reasoning. Overall, the manuscript requires substantial improvement in terms of originality, technical rigour, clarity, and logical structuring before it can be considered for publication. Comments can be referred to in the attached PDF.

Reviewer #2: This manuscript presents a comparative evaluation of physicochemical treatment configurations for electroplating wastewater and introduces an RSM-based optimization framework incorporating influent variability. The work is relevant and practically valuable, especially due to its full-scale industrial dataset and cost analysis. However, the manuscript requires major revisions, particularly in the mechanistic interpretation of treatment processes and discussion of human/environmental implications.

Address the following comments:

1. What are the discharge limits for the concerned pollutants and mention standards in the introduction?

2. What operating conditions critically influence the precipitation process?

3. State some of the previous RSM related studies for industrial effluents, their major outcomes and then correlate with the research gaps.

4. The manuscript consists of many short paragraphs. Authors are requested to properly format and improve the scientific language of manuscript.

5. TSS removal exhibits extreme variability (2-118 mg/L in f-EPW vs. 2 ± 0.3 mg/L in E3), why there is standard deviation missing at some places

6. Authors are requested to discuss the mechanism in detail for metal speciation and precipitation chemistry in the manuscript

7. Electroplating wastewater contains strong chelating agents such as EDTA, surfactants. How the mentioned coagulants break the metal-organic complexes and their kinetics effect the precipitation process?

8. The coefficients from the model should be more related to real chemistry

9. Why are emerging contaminants discussed only in the introduction but not in results and discussion?

10. The PC parameters used to evaluate the performance of the EPW investigated in this study were systematically analyzed using standardized and validated analytical methods. Mention the references or cite it

11. The manuscript report ~50% chloride removal which is unlikely as chloride mostly remain soluble. Explain it and perform the mass balance.

12. Unit and notations should be consistent

13. Some figures don’t have error bars

14. Discuss about sludge management, recyclability and how it effect the economic aspect

15. Can authors explain the superiority of lime treatment over current research trends such as adsorption, electrocoagulation, and membrane processes

16. The manuscript reports a significant quadratic effect of pH on nickel removal. Experimentally validate it by evaluating residual Ni over pH range to identify minimum solubility region, re-dissolution at higher pH

17. Correlate RSM optimum pH with minimum thermodynamic solubility

18. Surface interaction studies can be performed to analyse the role of adsorption alongwith precipitation. Isotherm model can be fitted.

19. Discuss the role of mixing speed during jar test, how it effects the diffusion?

20. Environmental and health implication can be discussed in separate section

**********

what does this mean?). If published, this will include your full peer review and any attached files.

If you choose “no”, your identity will remain anonymous but your review may still be made public.

**Do you want your identity to be public for this peer review?**  For information about this choice, including consent withdrawal, please see our Privacy Policy

Reviewer #1: No

Reviewer #2: No

**********

[NOTE: If reviewer comments were submitted as an attachment file, they will be attached to this email and accessible via the submission site. Please log into your account, locate the manuscript record, and check for the action link "View Attachments". If this link does not appear, there are no attachment files.]

To ensure your figures meet our technical requirements, please review our figure guidelines: https://journals.plos.org/plosone/s/figures

You may also use PLOS’s free figure tool, NAAS, to help you prepare publication quality figures: https://journals.plos.org/plosone/s/figures#loc-tools-for-figure-preparation.

NAAS will assess whether your figures meet our technical requirements by comparing each figure against our figure specifications.

Attachment Submitted filename: PONE-D-26-12528.pdf

Attachment Submitted filename: PONE-D-26-12528 Comments.docx

# Author comment: Author response to Decision Letter 1

**DOI:** 10.1371/journal.pone.0345965.r002

2 Jun 2026

We have carefully addressed all reviewer and editor comments. Our detailed responses are provided in the attached file named "Response to Reviewers", and are also copied in full below for your convenience.

Response to Reviewers

Journal of PLOS ONE | Manuscript No. PONE-D-26-12528

Dear Editor and Reviewers,

We thank the Editor and Reviewers for their thorough evaluation of our manuscript and for their valuable comments and constructive suggestions. All feedback has been carefully considered, and the manuscript has been extensively revised in response. These revisions have strengthened the manuscript's scientific rigor, clarity, technical depth, and overall organization.

All modifications in the revised manuscript are marked in red for clarity.

Below, we present a detailed point-by-point response to each comment.

Responses to the Academic Editor

Comment 1. Please ensure that your manuscript meets PLOS ONE's style requirements, including those for file naming.

Response:

The manuscript has been meticulously reformatted to comply with the official PLOS ONE guidelines, including manuscript structure, section organization, figure and table formatting, references, abbreviations, and file naming conventions. The revised version is now fully compliant with all journal formatting requirements.

Comment 2. Please note that PLOS One has specific guidelines on code sharing for submissions in which author-generated code underpins the findings in the manuscript.

Response:

No custom computational code or proprietary software was developed for this study. Statistical analyses and response surface methodology (RSM) modeling were conducted using the commercially available Design-Expert® software. All experimental data, model equations, operating conditions, and statistical outputs required to reproduce the results are provided in the manuscript and the Supporting Information files.

Comment 3. Thank you for stating in your Funding Statement:

“This research was supported and funded by the Deanship of Scientific Research at Imam Mohammad Ibn Saud Islamic University (IMSIU) (grant number IMSIU-DDRSP2602).“ Please provide an amended statement that declares *all* the funding or sources of support (whether external or internal to your organization) received during this study, as detailed online in our guide for authors at http://journals.plos.org/plosone/s/submit-now. Please also include the statement “There was no additional external funding received for this study.” in your updated Funding Statement.

Please include your amended Funding Statement within your cover letter. We will change the online submission form on your behalf.

Thank you for stating the following financial disclosure:

"This research was supported and funded by the Deanship of Scientific Research at Imam Mohammad Ibn Saud Islamic University (IMSIU) (grant number IMSIU-DDRSP2602)."

Please state what role the funders took in the study. If the funders had no role, please state: "The funders had no role in study design, data collection and analysis, decision to publish, or preparation of the manuscript."

If this statement is not correct you must amend it as needed.

Response:

One of the project requirements is to include the funding statement and acknowledgments exactly as they appear in the manuscript, without any changes. Thank you to keep it as it is (you can refer to this published paper: https://doi.org/10.1016/j.seppur.2025.133205 Funding Statement/ acknowledgement are the same).

The Funding Statement/ acknowledgement: “This research was supported and funded by the Deanship of Scientific Research at Imam Mohammad Ibn Saud Islamic University (IMSIU) (grant number IMSIU-DDRSP2602). The funder was actively involved in the study design, data collection and analysis, the decision to publish, and the preparation of the manuscript.

Comment 4. We note that your Data Availability Statement is currently as follows: [All relevant data are within the manuscript and its Supporting Information files.]

Please confirm at this time whether or not your submission contains all raw data required to replicate the results of your study. Authors must share the “minimal data set” for their submission. PLOS defines the minimal data set to consist of the data required to replicate all study findings reported in the article, as well as related metadata and methods (https://journals.plos.org/plosone/s/data-availability#loc-minimal-data-set-definition).

For example, authors should submit the following data:

-The values behind the means, standard deviations and other measures reported;

- The values used to build graphs;

- The points extracted from images for analysis.

Authors do not need to submit their entire data set if only a portion of the data was used in the reported study.

If your submission does not contain these data, please either upload them as Supporting Information files or deposit them to a stable, public repository and provide us with the relevant URLs, DOIs, or accession numbers. For a list of recommended repositories, please see https://journals.plos.org/plosone/s/recommended-repositories.

If there are ethical or legal restrictions on sharing a de-identified data set, please explain them in detail (e.g., data contain potentially sensitive information, data are owned by a third-party organization, etc.) and who has imposed them (e.g., an ethics committee). Please also provide contact information for a data access committee, ethics committee, or other institutional body to which data requests may be sent. If data are owned by a third party, please indicate how others may request data access.

Response:

We thank the editor for this clarification. We confirm that our submission now contains all raw data required to replicate all findings reported in the study. A new Supporting Information file (S1 Data) has been added, containing the underlying data. All relevant data for Figures 1, 2, 3, 6, 7, 8, 9, and 12 are now provided. The Data Availability Statement has been updated accordingly to: "All relevant data are within the manuscript and its Supporting Information files."

Comment 5. Thank you for stating the following in the Acknowledgments Section of your manuscript:

“This research was supported and funded by the Deanship of Scientific Research at Imam Mohammad Ibn Saud Islamic University (IMSIU) (grant number IMSIU-DDRSP2602).”

We note that you have provided additional information within the Acknowledgements Section that is not currently declared in your Funding Statement. Please note that funding information should not appear in the Acknowledgments section or other areas of your manuscript. We will only publish funding information present in the Funding Statement section of the online submission form.

Response:

One of the project requirements is to include the funding statement and acknowledgments exactly as they appear in the manuscript, without any changes. Thank you to keep it as it is (you can refer to this published paper: https://doi.org/10.1016/j.seppur.2025.133205 Funding Statement/ acknowledgement are the same).

The Funding Statement/ acknowledgement: “This research was supported and funded by the Deanship of Scientific Research at Imam Mohammad Ibn Saud Islamic University (IMSIU) (grant number IMSIU-DDRSP2602).

Comment 6. If the reviewer comments include a recommendation to cite specific previously published works, please review and evaluate these publications to determine whether they are relevant and should be cited. There is no requirement to cite these works unless the editor has indicated otherwise.

Response:

The reviewers did not recommend any specific references for citation.

Responses to Reviewer 1

We sincerely thank Reviewer 1 for the thorough and constructive evaluation. Reviewer 1's technically insightful comments have substantially strengthened the manuscript's scientific rigor, mechanistic depth, and overall coherence. All revisions in response to Reviewer 1 are marked in red in the revised manuscript.

Comment 1. When coagulation process is discussed, techniques like zeta potential are considered, which is missing as of now. Also, for any coagulation process, turbidity and coagulant dosage curve is important, which is also missing. Also, sludge characterization can be added to solidify your paper. It is suggested to add more data or results which are related to coagulation and flocculation process.

Response:

We thank the Reviewer for these insightful technical comments. We acknowledge that the coagulation and flocculation characterization section required further development, and we have revised the manuscript accordingly. We address each point in detail below.

Point 1 — Zeta Potential:

We concur that zeta potential is a fundamental parameter in coagulation science, as it governs colloidal stability and the charge-neutralization mechanism that determines destabilization efficiency. However, we note that direct zeta potential measurements were not conducted during the full-scale monitoring campaign because online zeta potential analyzers were unavailable at the Tunisian facility.

A dedicated paragraph has been added to the revised manuscript (page 12, lines 281-291) to discuss the role of zeta potential in the coagulation process.

"Coagulation: The dechromatized E1 effluents are combined with the alkaline E2 streams to produce a mixed effluent that undergoes coagulation. In this step, a chemical coagulant is introduced under rapid agitation to destabilize colloidal particles and suspended matter through charge neutralization and sweep floc formation. The effectiveness of destabilization is monitored using zeta potential measurements, which quantitatively assess the electrokinetic stability of the colloidal suspension. Optimal coagulation is achieved when the zeta potential approaches neutrality (approximately 0 mV), indicating that electrostatic repulsive forces have been sufficiently reduced to permit interparticle aggregation [30]. This process facilitates the formation of primary microflocs that act as nucleation sites for subsequent metal hydroxide precipitation, while also reducing turbidity and enhancing the settling characteristics of the treated effluent."

Point 2 — Turbidity and coagulant dosage curve:

The initial effluent E3 contains a negligible concentration of Total Suspended Solids (TSS) (2 ± 0.3 mg/L), as shown in Table 1. Because turbidity is determined by suspended particulate matter, it is negligible and not a relevant process performance indicator in this context. The primary water quality concerns in E3 are elevated concentrations of heavy metals and chlorides, which exceed regulatory discharge limits.

To further support this justification, Section 2.2 (pages 14 and 15, lines 356-366) has been revised to explicitly clarify that removal of suspended solids is not the primary challenge for this treatment system. Instead, the assessment focuses on heavy metal removal as the central performance criterion.

"The TSS of the initial effluent E3 is inherently negligible (2 ± 0.3 mg/L), indicating that turbidity, governed by the suspended particulate load, is also negligible and does not serve as a meaningful performance indicator or limiting parameter for this specific industrial wastewater matrix. The observed TSS variability in f-EPW (2 ± 1 – 118 ± 2 mg/L) is attributable to operational inconsistencies in the settling stage rather than to coagulation deficiency, confirming that suspended solids removal is not the primary challenge in this treatment system. In contrast, the most significant deficiencies are associated with HMs and chloride concentrations, which have been identified as the primary limiting factors for regulatory compliance and environmental impact. These parameters, therefore, constitute the central focus of the comparative performance assessment in this study."

Point 3 — Sludge Characterization:

We acknowledge that comprehensive quantitative sludge characterization was not conducted during the monitoring campaign. Nevertheless, preliminary field observations indicated that lime-based treatment (Configuration 3) produced higher sludge volumes due to calcium-based byproducts (CaCO₃, Ca(OH)₂). This configuration also showed improved settling behavior, increased dry solids content, and enhanced dewaterability, which partially offset disposal costs.

A dedicated paragraph summarizing these observations has been added to the new section "4. Environmental implications and research perspectives," which recommends standardized sludge characterization as future work. This paragraph is marked in red in the revised manuscript (pages 68-69, lines 1662-1674) (see also Response to Reviewer 2, Comment 14). Additionally, a preliminary discussion appears on pages 42-43 (lines 1039-1056) and is marked in red in the revised manuscript.

Comment 2. How is this method novel?

Response:

The innovation lies not in the individual techniques employed (RSM, coagulation, flocculation, precipitation), which are well established, but in reframing the optimization problem conceptually.

Conventional RSM-based studies treat influent composition as a fixed parameter, optimizing treatment conditions for a single, predefined wastewater quality. In contrast, this study introduces influent nickel concentration as an independent variable in the RSM-CCD design, yielding a predictive model of the form [Ni]f = f([Ni]i, pH, [A-PAM]). This model dynamically calculates optimal dosing based on the measured influent load, enabling real-time adaptive process control. The statistical identification of a significant pH–metal loading interaction (F = 5.70, p = 0.044) provides quantitative evidence that optimal parameters shift with influent composition. This variability-responsive framework, validated over 24 weeks at full industrial scale, represents a methodological advance beyond static laboratory optimizations.

Comment 3. How this sentence is connected to the previous lines? how this is problematic in terms of the heavy metal?

Response:

We acknowledge that the transition between the toxicological characterization of Cr(VI) and Ni(II) and the subsequent discussion of chloride-based coagulants was insufficiently explicit. The intended connection is as follows: after establishing the need to remove these heavy metals, the text addresses the persistent challenges of their removal. Specifically, chloride-based coagulants such as FeCl₃, although effective for metal precipitation, introduce secondary chloride contamination that can violate discharge standards.

The paragraph has been restructured into two distinct sections: one establishing the toxicological rationale and another outlining the technical limitations. This revision is presented on pages 7 and 8 (lines 172-183) of the revised manuscript (marked in red). The revised paragraph is as follows:

" Among heavy metal (HM) contaminants, hexavalent chromium (Cr(VI)) and divalent nickel (Ni(II)) are of particular concern because of their extensive use in electroplating and well-documented toxicological effects [7]. Cr(VI) is associated with carcinogenicity and acute ecotoxicity [2], whereas nickel is linked to carcinogenic, allergenic, and chronic ecological effects [8].

Consistent removal of these contaminants by conventional physicochemical treatments remains technically challenging for two primary reasons. First, chloride-based coagulants such as ferric chloride (FeCl₃), commonly used for heavy metal precipitation, introduce additional chloride into treated effluents. This can result in chloride concentrations exceeding discharge standards and limit opportunities for water reuse [2,9]. Second, strong metal–organic complexes formed by plating additives can inhibit standard precipitation mechanisms, allowing residual dissolved metals to persist even when chemical dosing and pH are appropriately controlled [2,10]."

Comment 4. NT 106.002 what is this? This is not clear. can you please add more?

Response:

NT 106.002, issued by the Tunisian Institute of Standardization (INNORPI), establishes the national regulatory framework for wastewater quality in Tunisia, specifically for discharges into receiving water bodies. This standard sets legally b

Attachment Submitted filename: Response to Reviewers.docx

# Peer review: Decision Letter 1

**DOI:** 10.1371/journal.pone.0345965.r003

**Authors:** Sovik Das

8 Jul 2026

Dear Dr. Ibrahimi,

Thank you for submitting your manuscript to PLOS ONE. After careful consideration, we feel that it has merit but does not fully meet PLOS ONE’s publication criteria as it currently stands. Therefore, we invite you to submit a revised version of the manuscript that addresses the points raised during the review process.

Please submit your revised manuscript by Aug 22 2026 11:59PM. If you will need more time than this to complete your revisions, please reply to this message or contact the journal office at plosone@plos.org. When you're ready to submit your revision, log on to https://www.editorialmanager.com/pone/ and select the 'Submissions Needing Revision' folder to locate your manuscript file.

- A letter that responds to each point raised by the academic editor and reviewer(s). You should upload this letter as a separate file labeled 'Response to Reviewers'.
- A marked-up copy of your manuscript that highlights changes made to the original version. You should upload this as a separate file labeled 'Revised Manuscript with Track Changes'.
- An unmarked version of your revised paper without tracked changes. You should upload this as a separate file labeled 'Manuscript'.

If you would like to make changes to your financial disclosure, please include your updated statement in your cover letter. Guidelines for resubmitting your figure files are available below the reviewer comments at the end of this letter.

If applicable, we recommend that you deposit your laboratory protocols in protocols.io to enhance the reproducibility of your results. Protocols.io assigns your protocol its own identifier (DOI) so that it can be cited independently in the future. For instructions see: https://journals.plos.org/plosone/s/submission-guidelines#loc-laboratory-protocols. Additionally, PLOS ONE offers an option for publishing peer-reviewed Lab Protocol articles, which describe protocols hosted on protocols.io. Read more information on sharing protocols at https://plos.org/protocols?utm_medium=editorial-email&utm_source=authorletters&utm_campaign=protocols.

As the corresponding author, your ORCID iD is verified in the submission system and will appear in the published article. PLOS supports the use of ORCID, and we encourage all coauthors to register for an ORCID iD and use it as well. Please encourage your coauthors to verify their ORCID iD within the submission system before final acceptance, as unverified ORCID iDs will not appear in the published article. *Only*  the individual author can complete the verification step; PLOS staff *cannot*  verify ORCID iDs on behalf of authors.

We look forward to receiving your revised manuscript.

Kind regards,

Sovik Das

Academic Editor

PLOS One

Journal Requirements:

If the reviewer comments include a recommendation to cite specific previously published works, please review and evaluate these publications to determine whether they are relevant and should be cited. There is no requirement to cite these works unless the editor has indicated otherwise.

Please review your reference list to ensure that it is complete and correct. If you have cited papers that have been retracted, please include the rationale for doing so in the manuscript text, or remove these references and replace them with relevant current references. Any changes to the reference list should be mentioned in the rebuttal letter that accompanies your revised manuscript. If you need to cite a retracted article, indicate the article’s retracted status in the References list and also include a citation and full reference for the retraction notice.

[Note: HTML markup is below. Please do not edit.]

Reviewers' comments:

Reviewer's Responses to Questions

**Comments to the Author**

Reviewer #2: All comments have been addressed

**********

2. Is the manuscript technically sound, and do the data support the conclusions?

Reviewer #2: Yes

**********

3. Has the statistical analysis been performed appropriately and rigorously?

Reviewer #2: No

**********

4. Have the authors made all data underlying the findings in their manuscript fully available?

The PLOS Data policy

Reviewer #2: Yes

**********

5. Is the manuscript presented in an intelligible fashion and written in standard English?

Reviewer #2: Yes

**********

Reviewer #2: How the speciation was performed? Suggestion is to use Visual Minteq OR appropriate reference for speciation.

**********

what does this mean?). If published, this will include your full peer review and any attached files.

If you choose “no”, your identity will remain anonymous but your review may still be made public.

**Do you want your identity to be public for this peer review?**  For information about this choice, including consent withdrawal, please see our Privacy Policy

Reviewer #2: No

**********

[NOTE: If reviewer comments were submitted as an attachment file, they will be attached to this email and accessible via the submission site. Please log into your account, locate the manuscript record, and check for the action link "View Attachments". If this link does not appear, there are no attachment files.]

To ensure your figures meet our technical requirements, please review our figure guidelines: https://journals.plos.org/plosone/s/figures

You may also use PLOS’s free figure tool, NAAS, to help you prepare publication quality figures: https://journals.plos.org/plosone/s/figures#loc-tools-for-figure-preparation.

NAAS will assess whether your figures meet our technical requirements by comparing each figure against our figure specifications.

# Author comment: Author response to Decision Letter 2

**DOI:** 10.1371/journal.pone.0345965.r004

13 Jul 2026

Response to Reviewers

Manuscript PONE-D-26-12528R1

Title: Integrated optimization of electroplating wastewater treatment: A comparative assessment of physicochemical treatment configurations and process enhancement using response surface methodology

Journal: PLOS ONE

Dear Dr. Sovik Das and Reviewers,

We thank the Editor and Reviewers for the careful re-evaluation of our manuscript and for confirming that all previous comments have been adequately addressed. We are grateful for the opportunity to respond to the remaining point raised in this round of review. Our point-by-point response is provided below, with reviewer comments reproduced in italics and our responses given underneath. Changes made to the manuscript are indicated where relevant.

Journal Requirements (Academic Editor)

Comment A: “If the reviewer comments include a recommendation to cite specific previously published works, please review and evaluate these publications to determine whether they are relevant and should be cited.”

Response: We thank the Editor for this note. Reviewer’s comment did not recommend any specific previously published work to be cited; the comment concerned the methodology used for chemical speciation (addressed in detail under Reviewer #2, Comment 3 below). No additional citation was therefore required in response to this point.

Comment B: “Please review your reference list to ensure that it is complete and correct. If you have cited papers that have been retracted, please include the rationale for doing so in the manuscript text, or remove these references and replace them with relevant current references. Any changes to the reference list should be mentioned in the rebuttal letter that accompanies your revised manuscript. If you need to cite a retracted article, indicate the article’s retracted status in the References list and also include a citation and full reference for the retraction notice.”

Response: We confirm that the full reference list has been carefully reviewed for this revision. All references have been checked for completeness and accuracy (authors, titles, journal names, years, volumes, and DOIs where available), and none of the cited works have been identified as retracted. No changes were made to the reference list in this revision round.

Reviewer #2

Comment 1: “All comments have been addressed.”

Response: We thank the reviewer for this positive assessment and for confirming that all comments raised in the previous round of review have been satisfactorily addressed.

Comment 2: Is the manuscript technically sound, and do the data support the conclusions? — “Yes.”

Response: We thank the reviewer for this favorable evaluation confirming that the manuscript is technically sound and that the data support the conclusions drawn.

Comment 3: Has the statistical analysis been performed appropriately and rigorously? — “No” (no specific methodological concern was described in the accompanying comments).

Response: We thank the reviewer for this evaluation and would welcome any further clarification, as the written comments provided relate exclusively to the chemical speciation point addressed below (Comment 6), with no additional statistical concern specified. We would like to take this opportunity to reaffirm the statistical rigor underlying our RSM model, as detailed in Section 3.2.2 (“Statistical Analysis and Model Validation”) of the manuscript. The experimental design and statistical analysis were performed using Minitab software based on a central composite design (CCD). Model significance was assessed by analysis of variance (ANOVA), yielding a highly significant model F-value of 111.37 (p < 0.0001), together with significant individual and interaction terms (pH, [Ni]i × pH, pH × flocculant dosage, and the quadratic pH term) consistent with the underlying precipitation chemistry (Table 5). Model adequacy was further confirmed through multiple validation metrics (Table 6): a correlation coefficient R² = 0.9917, an adjusted R² of 0.9832, and a predicted R² of 0.9352, whose close mutual agreement (difference < 0.2) rules out overfitting. The adequate precision ratio of 37.08, well above the minimum acceptable threshold of 4, further confirms an excellent signal-to-noise ratio. We trust that these results reaffirm the statistical soundness of the analysis, and we remain fully available to expand on any specific aspect the reviewer may wish to see clarified further in the manuscript.

Comment 4: Have the authors made all data underlying the findings in their manuscript fully available? — “Yes.”

Response: We thank the reviewer for confirming that the underlying data have been made fully available, in accordance with the PLOS Data Availability policy.

Comment 5: Is the manuscript presented in an intelligible fashion and written in standard English? — “Yes.”

Response: We thank the reviewer for confirming that the manuscript is clearly and correctly written in standard English.

Comment 6: “How was the speciation performed? Suggestion is to use Visual Minteq OR appropriate reference for speciation.”

Response: We thank the reviewer for this important observation. We would like to clarify that this point had already been addressed during the previous (major) revision round, in which we added a dedicated subsection (“Experimental Validation of the Quadratic pH Effect and Thermodynamic Correlation”) explicitly treating nickel speciation on the basis of an established thermodynamic reference rather than software-based modeling. Specifically, the residual soluble Ni fraction at each pH was interpreted using the classical Ni(II) hydrolysis/speciation framework of Stumm & Morgan (1996), which provides the solubility product of Ni(OH)₂ (Ksp = 5.5 × 10⁻¹⁶ at 25°C) together with the cumulative stability constants of the successive Ni(II) hydroxo-complexes (log β₁–β₄ = 4.97, 8.55, 11.33 and 13.60, respectively).

In that subsection, the theoretical soluble Ni(II) concentration was calculated as a function of pH from the full speciation expression:

[Ni] = [Ni²⁺] × (1 + β₁[OH⁻] + β₂[OH⁻]² + β₃[OH⁻]³ + β₄[OH⁻]⁴), with [Ni²⁺] = Ksp / [OH⁻]²

This theoretical solubility curve was directly compared with our experimental residual Ni concentrations measured across pH 6.0–12.0 on authentic industrial effluent (Fig. 12 of the manuscript). The comparison confirms that the RSM-derived quadratic pH effect (B² = +3.98, p < 0.0001) is thermodynamically consistent with Ni(OH)₂ speciation behavior, capturing (i) incomplete precipitation below pH 7.5, (ii) a minimum-solubility zone at pH 8.0–9.5 driven by Ni(OH)₂ precipitation together with co-precipitation onto Cr(OH)₃ flocs and A-PAM enmeshment, and (iii) re-dissolution above pH 9.5 due to the formation of soluble anionic hydroxo-complexes [Ni(OH)₃]⁻ and [Ni(OH)₄]²⁻.

It should be clarified that the residual Ni concentration was measured by ICP, a technique that quantifies the total elemental metal content but does not, by its nature, allow the different chemical forms present in solution to be distinguished. It is precisely for this reason that the interpretation of Ni speciation was performed a posteriori, using the thermodynamic framework of Stumm & Morgan (1996), by comparing the ICP-measured total Ni concentrations with the theoretical Ni(OH)₂ solubility curve calculated as a function of pH.

We believe that this thermodynamic speciation analysis, grounded in the well-established reference of Stumm & Morgan (1996) and validated against our own experimental data, satisfactorily addresses the reviewer’s request and reinforces the mechanistic robustness of the RSM model. Visual MINTEQ was not additionally used, as the analytical speciation framework already accounts for the full set of Ni(II) hydroxo-complexes relevant to our effluent matrix.

We thank the Academic Editor and Reviewer #2 once again for their constructive feedback, which has helped strengthen the scientific rigor of our manuscript. We hope that the revised version is now suitable for publication in PLOS ONE.

Sincerely,

Dr. Soumaya Ibrahimi

On behalf of all co-authors

Attachment Submitted filename: Response_to_Reviewers_auresp_2.docx

# Peer review: Decision Letter 2

**DOI:** 10.1371/journal.pone.0345965.r005

**Authors:** Sovik Das

6 Aug 2026

<p>Integrated optimization of electroplating wastewater treatment: A comparative assessment of physicochemical treatment configurations and process enhancement using response surface methodology

PONE-D-26-12528R2

Dear Dr. Ibrahimi,

We’re pleased to inform you that your manuscript has been judged scientifically suitable for publication and will be formally accepted for publication once it meets all outstanding technical requirements.

Within one week, you’ll receive an e-mail detailing the required amendments. When these have been addressed, you’ll receive a formal acceptance letter and your manuscript will be scheduled for publication.

An invoice will be generated when your article is formally accepted. Please note, if your institution has a publishing partnership with PLOS and your article meets the relevant criteria, all or part of your publication costs will be covered. Please make sure your user information is up-to-date by logging into Editorial Manager at Editorial Manager® and clicking the ‘Update My Information' link at the top of the page. For questions related to billing, please contact billing support.

If your institution or institutions have a press office, please notify them about your upcoming paper to help maximize its impact. If they’ll be preparing press materials, please inform our press team as soon as possible -- no later than 48 hours after receiving the formal acceptance. Your manuscript will remain under strict press embargo until 2 pm Eastern Time on the date of publication. For more information, please contact onepress@plos.org.

Kind regards,

Sovik Das

Academic Editor

PLOS One

Additional Editor Comments (optional):

Reviewers' comments:

Reviewer's Responses to Questions

**Comments to the Author**

Reviewer #2: All comments have been addressed

**********

2. Is the manuscript technically sound, and do the data support the conclusions?

Reviewer #2: Yes

**********

3. Has the statistical analysis been performed appropriately and rigorously?

Reviewer #2: Yes

**********

4. Have the authors made all data underlying the findings in their manuscript fully available?

The PLOS Data policy

Reviewer #2: Yes

**********

5. Is the manuscript presented in an intelligible fashion and written in standard English?

Reviewer #2: Yes

**********

Reviewer #2: I appreciate the authors' careful revision of the manuscript and their detailed responses to the reviewers' comments. The concerns raised in the previous review have been satisfactorily addressed, and the manuscript is now suitable for publication. I recommend acceptance.

**********

what does this mean?). If published, this will include your full peer review and any attached files.

If you choose “no”, your identity will remain anonymous but your review may still be made public.

**Do you want your identity to be public for this peer review?**  For information about this choice, including consent withdrawal, please see our Privacy Policy

Reviewer #2: No

**********

# Editor report: Acceptance letter

**DOI:** 10.1371/journal.pone.0345965.r006

**Authors:** Sovik Das

PONE-D-26-12528R2

PLOS One

Dear Dr. Ibrahimi,

I'm pleased to inform you that your manuscript has been deemed suitable for publication in PLOS One. Congratulations! Your manuscript is now being handed over to our production team.

At this stage, our production department will prepare your paper for publication. This includes ensuring the following:

* All references, tables, and figures are properly cited

* All relevant supporting information is included in the manuscript submission,

* There are no issues that prevent the paper from being properly typeset

You will receive further instructions from the production team, including instructions on how to review your proof when it is ready. Please keep in mind that we are working through a large volume of accepted articles, so please give us a few days to review your paper and let you know the next and final steps.

Lastly, if your institution or institutions have a press office, please let them know about your upcoming paper now to help maximize its impact. If they'll be preparing press materials, please inform our press team within the next 48 hours. Your manuscript will remain under strict press embargo until 2 pm Eastern Time on the date of publication. For more information, please contact onepress@plos.org.

You will receive an invoice from PLOS for your publication fee after your manuscript has reached the completed accept phase. If you receive an email requesting payment before acceptance or for any other service, this may be a phishing scheme. Learn how to identify phishing emails and protect your accounts at https://explore.plos.org/phishing.

If we can help with anything else, please email us at customercare@plos.org.

Thank you for submitting your work to PLOS ONE and supporting open access.

Kind regards,

PLOS ONE Editorial Office Staff

on behalf of

Prof Sovik Das

Academic Editor

PLOS One
